# Identification of a novel RNA giant nuclear body in cancer cells

**DOI:** 10.18632/oncotarget.6619

**Published:** 2015-12-15

**Authors:** Hong Zhou, Jiawei Zhang, Ying Gu, Xiaoxian Gan, Yichao Gan, Weiwei Zheng, Byung-Wook Kim, Xiaohua Xu, Xiaoya Lu, Qi Dong, Shu Zheng, Wendong Huang, Rongzhen Xu

**Affiliations:** ^1^ Department of Hematology, Key Laboratory of Cancer Prevention and Intervention, China National Ministry of Education, Second Affiliated Hospital, School of Medicine, Zhejiang University, Hangzhou 310009, China; ^2^ Cancer Institute of Zhejiang University, Hangzhou 310009, China; ^3^ Department of Diabetes Complications and Metabolism, Beckman Research Institute, City of Hope National Medical Center, Duarte, CA 91010, USA; ^4^ Zhejiang Academy of Medical Sciences, Hangzhou 310012, China; ^5^ Hangzhou First People's Hospital, Hangzhou 310006, China

**Keywords:** cancer cell, RNA giant nuclear body, nuclear RNA trafficking

## Abstract

Constitutive synthesis of oncogenic mRNAs is essential for maintaining the uncontrolled growth of cancer cells. However, little is known about how these mRNAs are exported from the nucleus to the cytoplasm. Here, we report the identification of a RNA giant nuclear body (RNA-GNB) that is abundant in cancer cells but rare in normal cells. The RNA-GNB contains a RNA core surrounded by a protein shell. We identify 782 proteins from cancer-associated RNA-GNBs, 40% of which are involved in the nuclear mRNA trafficking. RNA-GNB is required for cell proliferation, and its abundance is positively associated with tumor burden and outcome of therapies. Our findings suggest that the RNA-GNB is a novel nuclear RNA trafficking organelle that may contribute to the nuclear mRNA exporting and proliferation of cancer cells.

## INTRODUCTION

Cancer cells, unlike normal cells that only divide a finite number of times before they enter into a state of growth arrest or die, are able to maintain uncontrolled proliferation [1, 2]. However, few details are known about the mechanism that maintains the uncontrolled proliferation of cancer cells. The cell nucleus, which houses much of the genome and the machinery needed for its replication, maintenance, and expression, is crucial for the survival and proliferation of cells [3, 4]. A prominent feature of the nucleus is its ability to harbor a variety of subnuclear organelles, such as nucleoli, PML nuclear bodies (PML-NBs), Cajal bodies, nuclear speckles, paraspeckles, Sam68 nuclear bodies, Polycomb bodies and other uncharacterized nuclear bodies [5–11]. These nuclear structures have attracted great interest because of their close associations with human diseases, especially cancers. For example, PML-NBs not only contain large amounts of proteins that play important roles in apoptosis, regulation of senescence and tumor suppression but also are associated with other nuclear structures, including Cajal bodies and nucleoli, and share apoptotic regulators with these domains [12]. However, no nuclear bodies have been definitively linked to the uncontrolled proliferation of cancer cells so far.

EIF4E nuclear bodies were initially reported two decade ago [13–17] and thought to be involved in the exporting of nuclear mRNAs associated with cell proliferation [18–22]. However, these nuclear structures are ubiquitously present in all mammalian cells [13–17]. In contrast, studies have shown a positive correlation between increased eIF4E phosphorylation and cancer cell proliferation as well as tumorigenesis [23–26]. Consistently, highly phosphorylated eIF4E (p-eIF4E) was frequently observed in a variety of human cancers [23, 26]. Therefore, we hypothesized that cancer-associated nuclear bodies as detected by p-eIF4E antibody may exist in the nuclei of cancer cells.

## RESULTS

### A novel giant nuclear body is present in a variety of cancer cells

**Figure 1:**
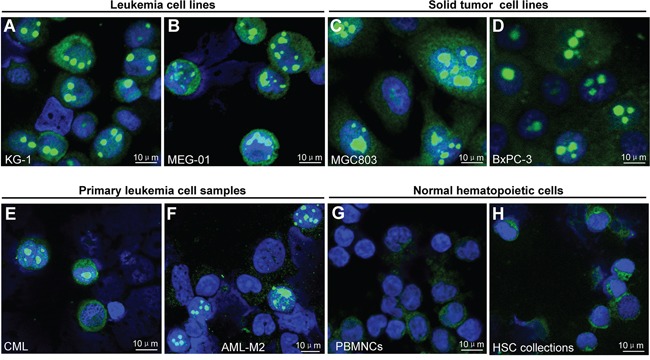
Novel giant nuclear bodies (GNBs) in cancer cells and normal blood cells **A–H.** Immunofluorescent staining images of novel giant nuclear bodies (green nuclear bodies) in the indicated leukemia (A–B) and solid tumor cell lines (C–D) and primary leukemia cell sample (E–F) and normal hematopoietic cell samples (G–H) with p-eIF4E antibody. Cell nuclei (blue) were stained using 4′,6-diamidino-2-phenylindole (DAPI).

To identify the putative cancer-associated nuclear body, we initially stained two human leukemia cell lines KG-1 (acute myeloid leukemia, AML) and MEG-01 (chronic myeloid leukemia, CML) by immunofluorescence staining with antibodies against phosphorylated eIF4E (p-eIF4E) or total eIF4E (t-eIF4E). Unexpectedly, a previously undefined giant nuclear body (GNB) was revealed exclusively in the nuclei of both KG-1 and MEG-01 cells by p-eIF4E antibody (Figure 1A-1B) but not by t-eIF4E antibody (Supplemental Figure 1A). GNBs were highly abundant in both KG-1 cells and MEG-01 cells (Figure 1A-1B). Typically, these GNBs were round, ranging in size from 2.0-5.0 μm, which is much larger than previously described eIF4E nuclear bodies [13–17]. In addition, we also observed several GNBs with irregular shape (Figure 1B), suggesting that GNB might be dynamic. To address whether it is a universal phenomenon in cancer cells, we screened a variety of cancer cell lines, including 18 hematopoietic cancer cell lines and 30 solid tumor cell lines, 35 primary leukemia samples and 7 normal hematopoietic cell samples (Supplementary Table S1). Similar GNBs were abundantly detected in all 48 different cancer cell lines by p-eIF4E antibody (Figure 1A–1D, Supplementary Figure S2A–2B, and Supplementary Table S1), but not by t-eIF4E antibody (Supplementary Figure S1A–1B). Significantly, GNBs were also observed in primary cancer cells from leukemia patients (Figure 1E–1F), indicating that GNBs are generated de novo in cancer cells. In contrast, GNBs were rarely found in normal peripheral blood mononuclear cells (PBMNCs) and hematopoietic stem cell collection (HSC collections) from conventional HSC collection (Figure 1G–1H).

**Figure 2:**
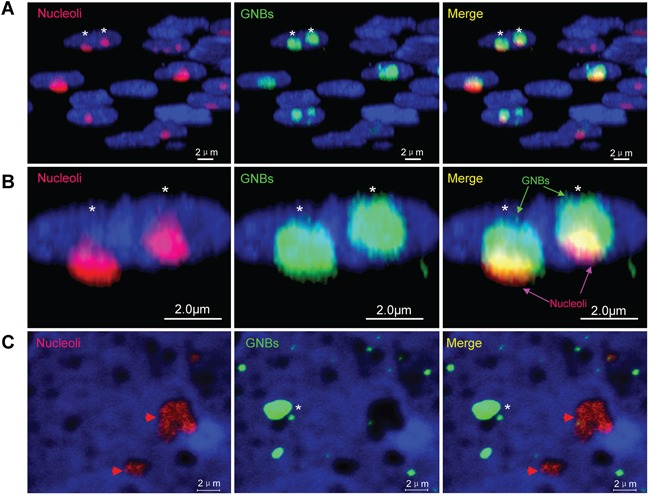
Giant nuclear bodies (GNBs) are different from nucleoli in cancer cells **A.** 3-D images of nuclei (blue), nucleoli (red bodies) and GNBs (green bodies) in cancer cells. GNBs attach on the surface of nucleoli. **B.** Magnified image of 3-D images of nuclei (blue), nucleoli (red bodies) and GNBs (green bodies) in cancer cells. Two GNBs attach on the surface of two nucleoli in the nucleus of cancer cells. **C.** GNBs (green bodies) were removed completely from nucleoli (red nuclear bodies) in nuclei of KG-1 cells after treatment with 0.05% NP40 for 5 min. Cell nuclei (blue) were stained using 4′,6-diamidino-2-phenylindole (DAPI).

To distinguish whether GNB was nucleolus, we investigated the co-localization of GNB and nucleolus in cancer cells. Approximately 60% of nucleoli (red bodies) were co-localized with GNBs (green bodies) in the nuclei of THP-1 leukemia cells (Supplementary Figure S3) as well as KG-1 leukemia cells (Supplementary Figure S4A). However, most GNBs in size were slightly larger than nucleoli in THP-1 cells (Supplementary Figure S3). To reveal the precise co-localization of the GNB and the nucleolus, we conducted a three-dimensional (3D) imaging by confocal microscopy and observed that the nuclear locations of GNBs and the nucleus are different. Almost all of GNBs (green bodies) were co-localized with the nucleoli (red bodies) in the nuclei (blue) of KG-1 leukemia cells (Figure 2A–2B). However, we found that depleting p-eIF4E with CGP57380, a small molecule inhibitor of MNK that exclusively phosphorylates eIF4E [26], greatly reduced the number of GNB whereas neither nucleoli (Supplementary Figure S4B–4C, asterisks) nor eIF4E nuclear bodies (Supplementary Figure S4C, arrowheads) were altered in KG-1 cells. In agreement with these results, GNBs (green bodies) could be removed completely from nucleoli (red bodies) after treating leukemia cells with nonionic detergent NP40 (0.05%) for 5 min (Figure 2C). Similar results were observed in KCL-22M leukemia cells (Supplementary Figure S5A). Together, these results indicate that the GNB is a distinct and undefined nuclear structure.

### GNB is composed of a shell with multiple drumstick-shaped subunits and inner structure

**Figure 3:**
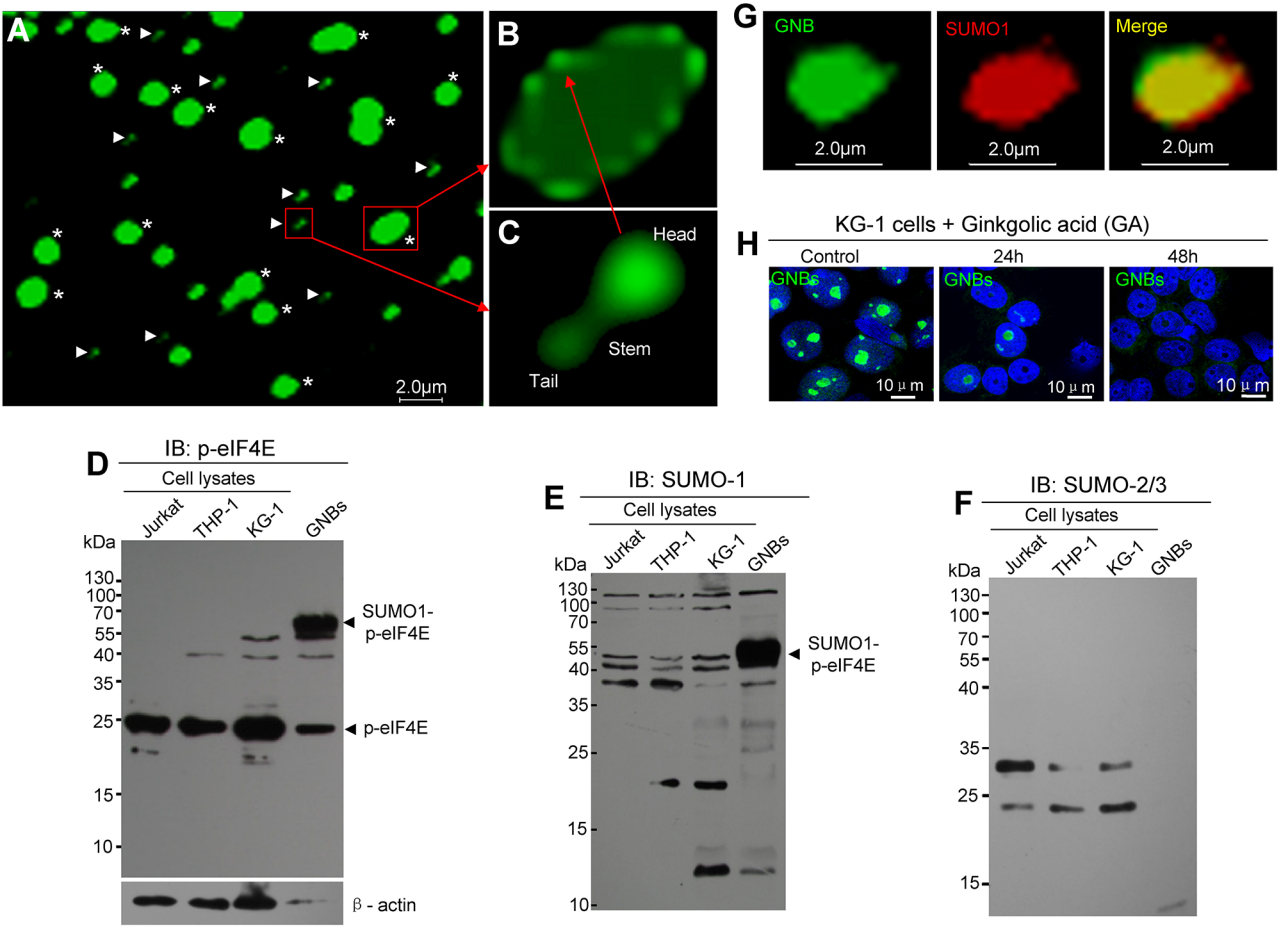
GNB contains a shell composed of drumstick-shaped subunits and inner structure **A.** Immunofluorescent staining images of purified GNBs (asterisks) and the subunits of GNB shells (arrowheads) from KCL-22M leukemia cells. **B.** Structure image of an intact GNB with a shell composed with several subunits. **C.** Structure image of a single subunit of the shell contains a head, a stem and a tail. **D–F.** Profile analyses of p-eIF4E protein (D), SUMO1-modified proteins (E), and SUMO2/3-modified proteins (F) of total cell lysates and purified GNB lysates using Western blotting with antibodies against p-eIF4E, SUMO1 or SUMO2/3. **G.** Two-color confocal images of p-eIF4E (green) and SUMO1(red) in GNB by immunofluorescence staining. GNBs were incubated with antibodies against p-eIF4E and SUMO1 followed by a secondary antibody labeled with FITC (p-eIF4E, green color) and Alexa Fluor 568(SUMO1, red color). A colocalization of p-eIF4E and SUMO1 in the GNB was evident (merge, yellow). **H.** SUMOylation inhibition decreased GNBs in KG-1 leukemia cells. Leukemia cells were treated with GA at 200 μM for different time points and then collected for analyses of GNBs using immunofluorescence staining. Cell nuclei (blue) were stained using DAPI.

To reveal the morphology and structure features of GNB, we isolated and purified GNBs from leukemia cells using dounce homogenization followed by immunopurification with p-eIF4E antibody. We obtained two types of purified particles: large round particles (2.0 – 5.0 μm in diameter) (Figure 3A, asterisks, and Figure 3B) and small drumstick-shaped particles (0.6 μm in length) (Figure 3A, arrows, and Figure 3C). The large round particles were similar to the GNBs observed in cell nuclei (Supplementary Figure S5B), while the small drumstick-shaped particles consisted of a spherical head (0.3 μm in diameter), stem (0.1 μm in length) and spherical tail (0.2 μm in diameter) (Figure 3C). Intriguingly, nearly all large particles contained a shell and inner structure (Figure 3A–3B). Moreover, the shell of large particle was composed of multiple small drumstick-shaped particles (Figure 3A–3C). On the basis of size and structure features of large and small particles, we propose that the large round particle is the GNB observed in the nucleus of leukemia cells, whereas small drumstick-shaped particle could be the subunit of the GNB shell. Similar GNBs were observed in KG-1 leukemia cells (Supplementary Figure S5C). To further confirm that GNB was different from the nucleolus, we detected nucleolin, which is one of the most abundant proteins in nucleoli [27, 28], in purified GNBs. Both immunofluorescence staining and western blot analyses failed to detect nucleolin in most of GNBs (Supplementary Figure S6A) or GNB lysates (Supplementary Figure S6B) from KG-1 leukemia cells, indicating that GNB is not nucleolus indeed.

### The role of SUMOylation in GNB formation

Because GNB is detected by the p-eIF4E antibody, we assume that p-eIF4E is a key component of GNB. To further reveal the properties of GNB at the molecular levels, we compared the potential post-translational modifications of p-eIF4E in purified GNB lysates and total cell lysates by western blotting. Two prominent proteins with molecular masses of ∼49 kDa and ∼25 kDa were identified by p-eIF4E antibody. In GNB lysates, the amount of ∼49 kDa protein was much higher than that of ∼25 kDa protein (Figure 3D). In contrast, the most abundant protein in total cell lysates was the 25 kDa protein whereas 49 kDa protein bands were very faint (Figure 3D). Because mammalian eIF4E can be modified by SUMO molecules, and SUMO1 modification of p-eIF4E induces the translation of a subset of proteins that are essential for cell proliferation and preventing apoptosis (29), we predicted that the 25 kDa protein might be free p-eIF4E whereas the 49 kDa protein might represent SUMOylated p-eIF4E. To test this, we probed western blots of the lysates with antibodies against SUMO1 or SUMO2/3. As expected, the 49 kDa protein, but not the 25 kDa protein, was detected by anti-SUMO1 antibody (Figure 3E) but not by SUMO2/3 antibody (Figure 3F). In addition, we also observed that the 49kDa protein could be detected by ubiquitin antibody (Supplementary Figure S6C). To further confirm these observations, immunofluorescence staining in conjunction with confocal microscopy imaging was conducted. SUMO1 protein was detected in GNBs and a large proportion of co-localization was apparent for p-eIF4E and SUMO1 (Figure 3G). These results indicate that eIF4E in GNBs may be dually modified by SUMO1 and phosphorylation.

To validate whether SUMOylation was required for GNB formation, we treated KG-1 leukemia cells with the SUMOylation inhibitor ginkgolic acid (GA) and then examined GNBs by immunofluorescence staining. The levels of GNB (green bodies) was markedly decreased at 24 h and disappeared at 48h after GA treatment (Figure 3H). Similar results were observed in THP-1 and KCL-22M leukemia cells (Supplementary Figure S7A–7B). To determine whether SUMOylation was essential for the growth of cancer cells, we next examined the effect of the SUMOylation inhibitor ginkgolic acid (GA) on the proliferation of leukemia cells. KG-1 leukemia cells were treated with GA for 72 h and then collected for analyses of cell viability and GNB levels. We observed that GA decreased the levels of GNB with a concomitant inhibition of leukemia cell proliferation (Supplementary Figure S7C). Our results suggest that the SUMOylation by SUMO1 may be important for GNB stability.

### GNB is a highly complex nuclear protein structure with a RNA core

**Figure 4:**
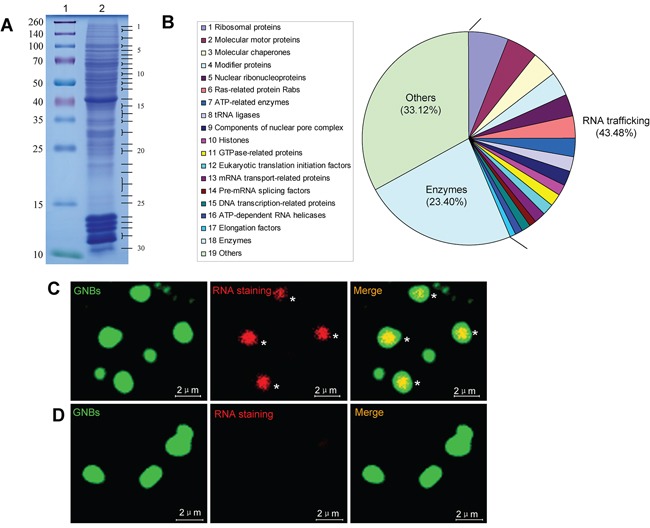
GNB is a highly complex nuclear protein structure with a RNA core **A.** Analysis of GNB proteins separated by SDS-PAGE. GNB proteins were separated by SDS-PAGE on a 12% polyacrylamide gel and stained with Coomassie blue (lane 2). Molecular masses of known proteins separated in the same gel are indicated on the left of the image (lane 1). **B.** Functional classes of 782 different proteins identified in proteomic analyses of purified GNBs from leukemia cells. **C.** RNA staining of GNBs with acridine orange (AO). All intact GNBs contained a RNA core (red) surrounded by a shell of GNBs. **D.** RNase treatment eliminated RNAs within GNBs.

To identify the protein components of GNB, we performed a proteomic analysis of purified GNBs from KG-1 leukemia cells. GNB proteins were separated by one-dimensional SDS-PAGE and stained with Coomassie Blue (Figure 4A). This gel was sequentially cut into 30 fragments for proteomic analysis.

A total of 782 different proteins were identified in the 30 fragments on the basis of at least five different peptides (Figure 4B, Supplementary Table S2, and Supplementary Table S3), 340 (43.48%) of these proteins corresponded to nuclear RNA trafficking and could be further grouped into 17 classes: (1) 47(6.01%) ribosomal proteins; (2) 37 (4.73%) molecular motor proteins (e.g., myosin, actin, and tubulin); (3) 31(3.96%) molecular chaperones (e.g., heat shock proteins and T-complex proteins); (4) 29 (3.71%) modifier proteins (e.g., ubiquitin-related enzymes, SUMO1, cullin and calcyclin-binding protein); (5) 26 (3.32%) nuclear ribonucleoproteins, including hnRNPs (e.g., hnRNPA2B1, hnRNPA1, hnRNPR, and hnRNPU) and small nuclear ribonucleoproteins; (6) 25(3.2%) Ras-related protein Rabs involved in protein transport; (7) 22(2.81%) ATP-related enzymes, including ATP synthases and ATPases; (8) 18 (2.3%) tRNA ligases; (9) 16 (2.05%) components of nuclear pore complex (NPC) (e.g., nuclear pore membrane glycoprotein and Nucleoporins); (10) 15(1.92%) histones; (11) 14(1.79%) GTPase-related proteins; (12) 14 (1.79%) eukaryotic translation initiation factors (e.g., eIF4A1, eIF4E, and eIF3D); (13) 13 (1.66%) mRNA transport-related proteins [e.g., CRM1, RanGTP, Importins, exportins and LRPPRC (LRP130)]; (14) 9 (1.15%) pre-mRNA splicing factors, including pre-mRNA-processing factors and splicing factors; (15) 9 (1.15%) DNA transcription-related proteins; (16) 9(1.15%) ATP-dependent RNA helicases (e.g., DDX3, DDX39B, eIF4A1, and DHX30); and (17) 6 (0.77%) elongation factors [e.g., elongation factor Tu (TUFM), EEF2, GFM1 and EEF1D]. Interestingly, 183(23.40%) various types of enzymes in addition to 61 enzymes involved in RNA trafficking and 259 (33.12%) other proteins involved in other different biological processes were also identified (Figure 4B, Supplementary Table S3, and Supplementary Table S4). To verify these observations, a panel of randomly selected proteins, such as myosin-9, LRP130, CRM-1, DDX39B, eIF4A1, tubulin, β-actin, hnRNPA1 and histone 2A, were validated by western blotting analysis. All validated proteins were detected in GNBs (Supplementary Figure S8), suggesting that most of proteins identified by MS are present in GNBs.

The identification of large amounts of proteins involved in RNA trafficking suggests that GNB may contain RNAs. To test this, we next performed RNA staining of purified GNBs from KG-1 cells using acridine orange (AO). Indeed, intact GNBs were strongly positive for RNA staining (Figure 4C, asterisks). RNAs were located in the center and surrounded by the shell of GNB (Figure 4C, asterisks). To confirm these observations, purified GNBs were treated with RNase at 20 μg/ml for 30 min and then performed RNA staining with AO. As expected, RNase treatment dramatically reduced RNA levels of GNBs, indicating that GNBs contained RNAs indeed (Figure 4D). In addition, we also performed DNA staining of purified GNBs with 4′,6-diamidino-2-phenylindole (DAPI) and failed to detect DNA in the GNBs (Supplementary Figure S9).

### RNA-GNB levels are positively correlated with tumor burden and clinical outcome

**Figure 5:**
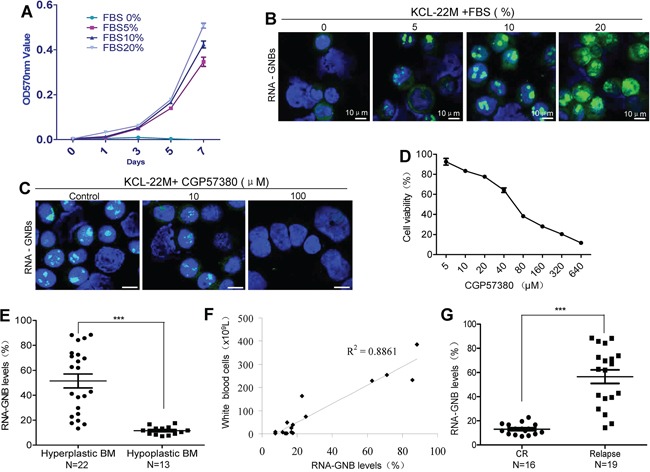
GNB abundance is positively correlated with cell proliferation, tumor burden and outcome of therapy in human leukemia **A–B.** A positive correlation exists between cell proliferation (A) and GNB levels (B). Kcl-22M cells were treated with the mitogen FBS at various concentrations for indicated times and then collected for analyses of cell proliferation by MTT and GNBs by immunofluorescence staining. **C–D.** Depletion of GNBs with the small molecule inhibitor CGP57380 inhibited proliferation of leukemia cells with a dose-dependent manner. Kcl-22M cells were treated with the indicated concentrations of CGP57380 for 72 h and then analyzed for GNBs by immunofluorescent staining(C) and Cell viability by MTT (D). **E.** RNA-GNB abundance was positively associated with the malignant proliferative potential of the bone marrow (BM) in patients with leukemia. ***P < 0.001. **F.** A positive correlation between RNA-GNB levels and peripheral white blood cell count were observed in leukemia patients (R2=0.89). Note: Normal blood cell count is below 1 × 10^9^L. **G.** RNA-GNB abundance was associated with response to chemotherapies. ***P < 0.001.

Because RNA-GNB is predominantly present in proliferative cells, we next assessed the correlation between RNA-GNB level and cell proliferation using mitogen fetal bovine serum (FBS), which could potently increase levels of SUMO1-phospho eIF4E [29]. As expected, we found that FBS promoted cell proliferation in a dose-dependent manner. In parallel, RNA-GNB levels were concomitantly elevated in Kcl-22M cells (Figure 5A–5B). Conversely, FBS starvation induced depletion of RNA-GNBs with a concomitant cell growth arrest in Kcl-22M cells (Figure 5A–5B). To verify this, we next analyzed the effect of reducing RNA-GNBs on the proliferation of leukemia cells. Kcl-22M leukemia cells were treated with CGP57380 for 72 h and then collected to analyze cell viability and RNA-GNB levels. We found that there was a positively correlation between decreased levels of RNA-GNB and inhibition of leukemia cell proliferation (Figure 5C–5D). In addition, we also compared the levels of RNA-GNBs and proliferating nuclear antigen Ki-67 in leukemia cells using immunofluorescence staining. Our results showed that approximately 80% of RNA-GNB-positive cells also expressed Ki-67 antigen (Supplementary Figure S10).

To determine whether RNA-GNB abundance was associated with the hyperproliferative phenotype in human cancer, we analyzed the RNA-GNB levels in relation with the bone marrow cellularity in various leukemia. RNA-GNB was detected by immunofluorescence staining. Results were expressed as positive percentage of cells with abundant RNA-GNBs, and the mean values in hypercellular leukemia (hyperplastic BM) and nonhypercellular leukemia (hypoplastic BM) cases were 51.48±5.59 and 11.30±0.79, respectively (*P* < 0.001). These data indicate that RNA-GNB levels in leukemia patients with hyperplastic bone marrow are much higher than those with hypoplastic bone marrow. These results suggest that the levels of RNA-GNBs positively correlated with the malignant proliferative potential of the bone marrow in leukemia patients (Figure 5E). Consistent with this, we found that RNA-GNB level was positively correlated with the burden of leukemia cells in peripheral blood of leukemia patients (Figure 5F, R2=0.89). In addition, we also found that leukemia patients with highly abundant RNA-GNBs (56.55±5.62) exhibited poorer outcome of chemotherapies whereas patients with low abundant RNA-GNBs (13.05±1.14) displayed better outcome of chemotherapies (Figure 5G). These data indicate that RNA-GNB abundance is not only positively correlated with the proliferative potential of cancer cells and tumor burden but is also an indicator of poorer outcome to chemotherapies in leukemia.

## DISCUSSION

In this study, we have identified a novel RNA giant nuclear body (RNA-GNB) that is associated with cancer cell proliferation. RNA-GNB is a giant nuclear structure with a RNA core surrounded by a protein shell. It is predominantly present in cancer cells, including a variety of cancer cell lines and primary cancer cells from leukemia patients. In addition, we also demonstrate that RNA-GNB abundance is positively associated with tumor burden and outcome of therapy in human leukemia.

Our results reveal that the RNA-GNB might be a novel nuclear organelle involved in nuclear mRNA export. RNA staining shows that RNA-GNB contains a large RNA core. Proteomics indicate that nearly half of RNA-GNB proteins are involved in nuclear RNA trafficking. Consistently, previous studies showed that many proteins identified in RNA-GNBs, such as actin, EF1α, hnRNP A/B, and tRNA synthetases, are components of the retrovirus RNA trafficking granule [30]. In addition, there is evidence that SUMOylation of proteins is involved in RNA trafficking. For instance, SUMOylated La protein affects mRNA trafficking in axons [31] and SUMOylated hnRNPA2B1 controls the sorting of miRNAs into exosomes [32]. These results indicate that RNA-GNB may be involved in mRNA trafficking from the nucleus to the cytoplasm.

Notably, our study has shown that the abundance of RNA-GNB is positively correlated with cell proliferation. Increased RNA-GNB number promotes cell proliferation whereas decreased RNA-GNB number inhibits cell proliferation. Finally, we find that RNA-GNB is present in more than one half of leukemia patients. Most importantly, RNA-GNB abundance is positively associated with the proliferative potential of cancer cells, tumor burden and poorer outcome of therapy in leukemia patients. These findings strongly suggest that RNA-GNB may play important roles in cancer cells.

In summary, our studies have identified RNA-GNB as a potential nuclear mRNA trafficking organelle. Further studies will be required to characterize the components of proteins and mRNAs in RNA-GNB and reveal their functions. This will help elucidate how RNA-GNBs form and regulate the uncontrolled proliferation of cancer cells.

## MATERIALS AND METHODS

### Cell lines and culture

In all, 18 various human hematopoietic malignant cell lines and 30 different solid tumor cell lines were used in this study and their names and subtypes are listed in Supplementary Table S1. Hematopoietic malignant cells were cultured in RPMI-1640 supplemented with 10% fetal calf serum (FCS) at 37°C in a 95% air, 5% CO_2_ humidified incubator. Solid tumor cells were cultured in DMEM supplemented with 10% fetal calf serum (FCS).

### Normal human blood samples and human leukemia cell samples

Normal blood cells and primary leukemia cell samples were isolated from healthy volunteers or leukemia patients with their informed consent in accordance with the Declaration of Helsinki. All experiments were approved by the ethics committee of Second Affiliated Hospital, School of Medicine, Zhejiang University.

### Immunofluorescence staining

Cells were fixed with freshly prepared 3.7% paraformaldehyde in PBS (pH 7.2) for 20 min at room temperature on slides. Cells were then blocked and permeabilized with PBS containing 10% FBS and 0.1% Tween-20 for 30 min at room temperature. Staining of cells with primary antibodies was performed overnight at 4°C in PBS containing 10% FBS, and then with a FITC- or rhodamine-conjugated secondary antibodies for 1 h at room temperature. After three washes with PBS, the slides were mounted in Vectashield with DAPI (4′, 6′-diamidino-2-phenylindole; Vector Laboratories, Burlingame, CA) and sealed. Fluorescence was observed with a Zeiss Confocal Laser Scanning Microscope.

### Immunopurification of GNBs from leukemia cells

GNBs were purified from leukemia cells as following protocol. 2 × 10^8^ cells were harvested by centrifugation at 2,000 rpm for 5 minutes at 4°C. After washed three times with PBS, cells were resuspended in PBS containing protease inhibitors and transferred to a 7 mL Dounce tissue homogenizer for dounce homogenization (typically 20 strokes of the tight pestle). The homogenized suspension was collected for removing intact cells and undisrupted nuclei by centrifugation at 1,000 rpm for 5 minutes at 4°C. The supernatant was transferred to a fresh tube and centrifuged at 6,000 rpm for 5 minutes at 4°C. The pellet (containing GNBs) was resuspended in 1ml of PBS buffer containing protease inhibitors and pretreated with 5 μg of normal rabbit IgG for 1hours followed by 50 μl protein A/G beads for 30min at 4°C. After centrifugation at 1,000 rpm for 5 minutes at 4°C, the supernatants were transferred to a fresh tube and incubated with 2 μg of anti-p-eIF4E antibody overnight at 4°C and then incubated with 50 μl of protein A/G beads for 2 hours at 4°C. After washed 6 times with PBS by centrifugation at 1,000 rpm for 5 minutes at 4°C, 0.5 ml of elution buffer (0.1 M glycine-HCl, pH 2.8) was added to elute GNBs from protein A/G beads for 10min at room temperature and then 1/10th volume of 1M Tris-HCl (pH 8.8) was added to neutralize the buffer. The quality of purified GNBs was examined by immunofluorescence staining under confocal laser scanning microscope.

### Western blot analysis

Cells were washed twice with 1× phosphate-buffered saline (PBS, pH 7.2), and total cellular protein was extracted with RIPA buffer. Protein samples were subjected to SDS-PAGE (12% polyacrylamide gels), and then transferred to nitrocellulose membranes (Whatman, Pittsburgh, PA), blocked with 5% nonfat milk in TBS-Tween 20 (TBS-T) and incubated with primary antibodies overnight at 4°C. After three washes with TBS-T, membranes were probed with a horseradish peroxidase–conjugated secondary antibody (Cell Signaling, Beverly, MA) for 1 h at room temperature, and signals were detected by chemiluminescence (Super Signal West Pico; Thermo, Waltham, MA).

### Antibodies

The antibodies used for immunofluorescence staining were as follows: p-eIF4E from Abcam(Cambridge, MA), eIF4E, nucleolin, SUMO-1 and Ki-67 were from Cell Signaling Technology (Beverly, MA).

The antibodies used for western blot analysis were as follows: p-eIF4E, eIF4E, nucleolin, myosin-9, LRP-130, CRM-1, DDX39B, eIF4A1, Tubulin, β-actin, hnRNPA1, SUMO1, SUMO2/3 and Histone-2A antibodies. All antibodies except p-eIF4E antibody (Abcam, Cambridge, MA) were purchased from Cell Signaling Technology (Beverly, MA).

### Separation of GNBs from nucleoli on the cell nuclei

Cells were collected by centrifugation at 2500 rpm for 5 minutes at 4°C. After removing the supernatant, the cell pellet was lysated with 0.5ml of buffer A(10 mM Tris PH7.5,10 mM NaCl, 3 mM MgCl_2_, 1 mM PMSF, 0.05%NP40) for 5min and then centrifuged at 700 rpm, 5min at 4°C. The supernatant was carefully removed and the nuclei were washed with 1.5ml of Buffer A without NP40 by centrifugation at 700 rpm, 5min at 4°C. The separation effect of GNBs from nucleoli in cell nuclei was examined with immunofluorescence staining under confocal laser scanning microscope.

### RNA staining of GNBs with acridine orange (AO)

Purified GNBs were washed in PBS (pH7.2) and then fixed in 3.7% paraformaldehyde (PFA) in PBS for 20 min at room temperature. After washing once with PBS, GNBs were incubated with AO in distilled water solution (10 μg/ml) for 1h. After washing 3 times with PBS, GNBs were observed for RNA staining under confocal laser scanning microscope.

### Proteomics analysis of GNBs

GNB protein isolation: GNB proteins were separated by one-dimensional SDS-PAGE and stained with Coomassie Blue. This gel was sequentially cut into 30 fragments for proteomic analysis.

In-gel Digestion: The gel slices were cut into small cubes of 1 mm^3^ that were washed three times with 500 μl ddH2O. The gel pieces were dehydrated by the addition of 500 ul acetonitrile and dried in a speed-vacuum concentrator (Thermo Scientific, San Jose, CA). Disulfide bonds were cleaved by incubating the samples with 200 μl of 10 mM DTT in 25 mM ammonium bicarbonate for 1 h at 56°C. Alkylation of cysteines was performed by incubating the samples with 200 μl of 55 mM iodoacetamide in 25 mM ammonium bicarbonate for 45 min at room temperature in darkness. The gel pieces were dehydrated again and supernatant was discarded after each step. Gel pieces were covered with trypsin solution (10 ng/μl in 50 mM ammonium bicarbonate). After a 30-min incubation of ice, the remaining trypsin solution was removed, and 25 μl of 50 mM ammonium bicarbonate was added. Proteolysis was performed overnight at 37°C and stopped by adjusting the samples to 5% formic acid. The peptides in gel was extracted once by 200 μl 0.1% formic acid in 50% acetonitrile and twice by 200 μl 100% acetonitrile. All the supernatant was combined and vacuum-dried.

LC-ESI-MS/MS Analysis by Q Exactive: The peptides were resuspended in buffer A (2% ACN, 0.1% FA) and centrifuged at 20000g for 2min. The supernatant was transferred into sample tube and loaded onto an Acclaim PepMap 100 C18 trap column (Dionex, 75 um × 2 cm) by EASY nLC1000 nanoUPLC (Thermo Scientific) and the peptide was eluted onto an Acclaim PepMap RSLC C18 analytical column (Dionex, 50 um × 15 cm). A 34min gradient was run at 300 nl/min, starting from 5 to 30% B (80% ACN, 0.1% FA), followed by 2 min linear gradient to 40% B, then 2 min to 80% B, and maintenance at 80% B for 4 min. The peptides were subjected to NSI source followed by tandem mass spectrometry (MS/MS) in Q Exactive (Thermo Scientific) coupled online to the UPLC. Intact peptides were detected in the Orbitrap at a resolution of 70000. Peptides were selected for MS/MS using 25% NCE with 4% stepped NCE; ion fragments were detected in the Orbitrap at a resolution of 17500. A data-dependent procedure that alternated between one MS scan followed by 15 MS/MS scans was applied for the top15 precursor ions above a threshold ion count of 4E4 in the MS survey scan with 2.5 s dynamic exclusion. The electrospray voltage applied was 1.8 kV. Automatic gain control (AGC) was used to prevent overfilling of the ion trap; 2E5 ions were accumulated for generation of MS/MS spectra. For MS scans, the m/z scan range was 350 to 1600 Da.

Database Search: The instrument data files (.raw) were converted into .mgf files by Proteome Discoverer (ver. 1.3.0.339, Thermo). Peptide and protein identifications were performed using the Mascot search engine (ver. 2.3.0, Matrix Science). Tandem MS spectra were searched against swissprot human database. Database searching was restricted to tryptic peptides. Carbamidomethyl (C) was selected as fixed, acetyl (Protein N-ter), oxidation (M) as variable modifications, 2 missed cleavage allowed and precursor error tolerance at 10 ppm, fragment deviation at 0.02 Da.

### Statistical analysis

All statistical analyses were performed with Prism GraphPad. Results are expressed as means ± SEM. Differences were evaluated by *t*-test analysis of variance and *P* values less than 0.05 were considered statistically significant.

## SUPPLEMENTARY FIGURES AND TABLES








