# Supplementary material for: Identification of a novel RNA giant nuclear body in cancer cells
Source: Oncotarget. 2015 Dec 15;7(4):4724–34. doi: 10.18632/oncotarget.6619 (PMC4826238; doi:10.18632/oncotarget.6619)
Supplement: Supplementary file 1 [file oncotarget-07-4724-s001.pdf]

SUPPLEMENTARY FIGURES AND TABLES

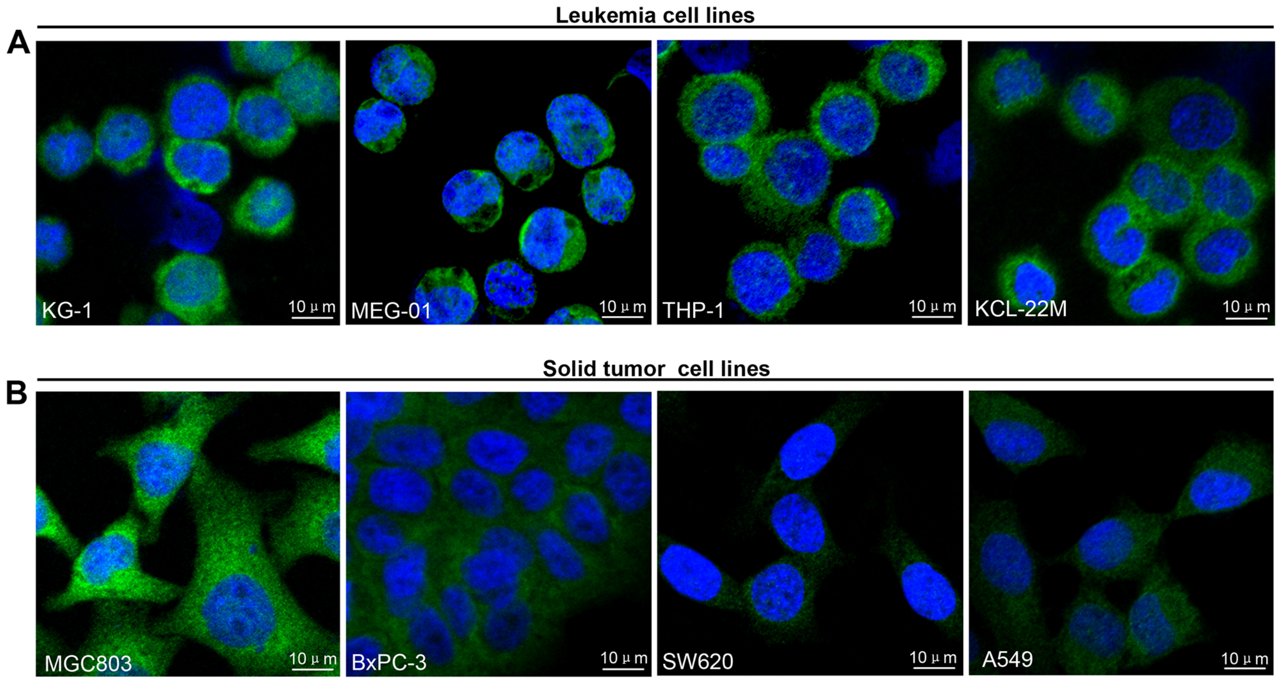

Supplementary Figure S1: Giant nuclear bodies are not detected by t-eIF4E antibody in cancer cells. A. Confocal microscopy images of GNB in leukemia cell lines KG-1, MEG-01, THP-1 and KCL-22M. **B.** Confocal microscopy images of GNB in solid tumor cell lines MGC803, BxPC3, SW620 and A549. No GNBs were detected in both leukemia and solid tumor cell lines using immunofluorescence staining with t-eIF4E antibody. Cell nuclei (blue) were stained with 4′,6-diamidino-2-phenylindole (DAPI).

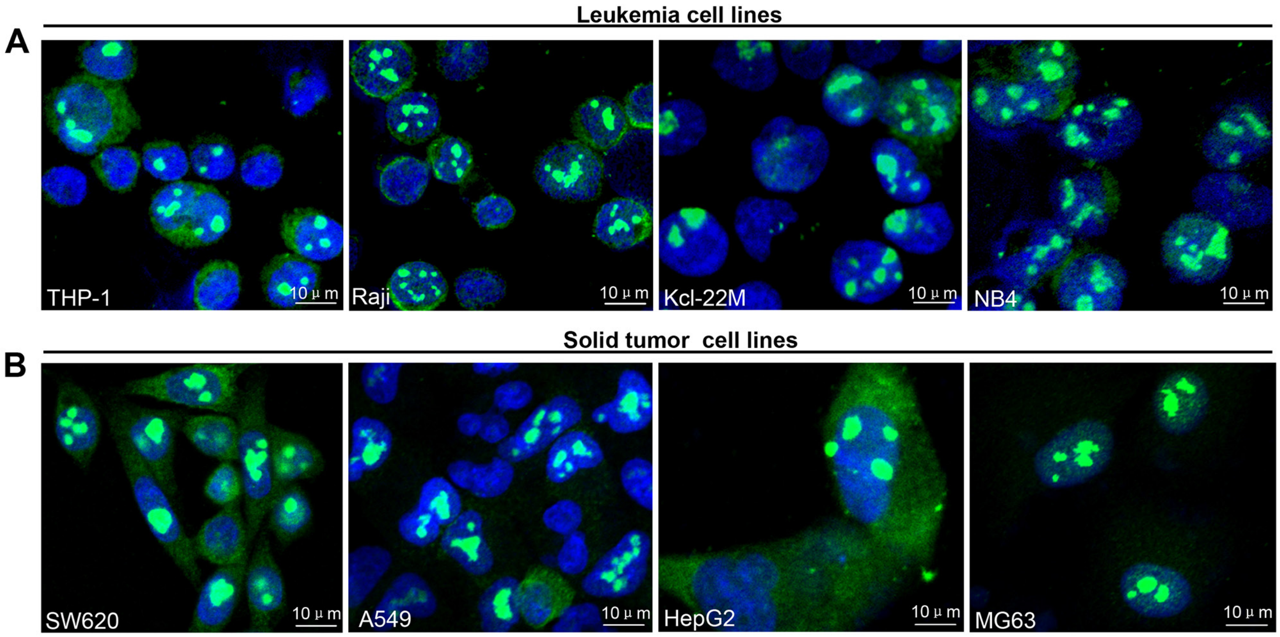

Supplementary Figure S2: GNBs are abundantly present in various leukemia and solid tumor cell lines. A–B. Immunofluorescent staining images of giant nuclear bodies (green nuclear bodies) in leukemia cell lines (THP-1, Raji, Kcl-22M and NB4) (A) and solid tumor cell lines (SW620, A549, HepG2 and MG63) (B) with p-eIF4E antibody. Cell nuclei (blue) were stained with DAPI.

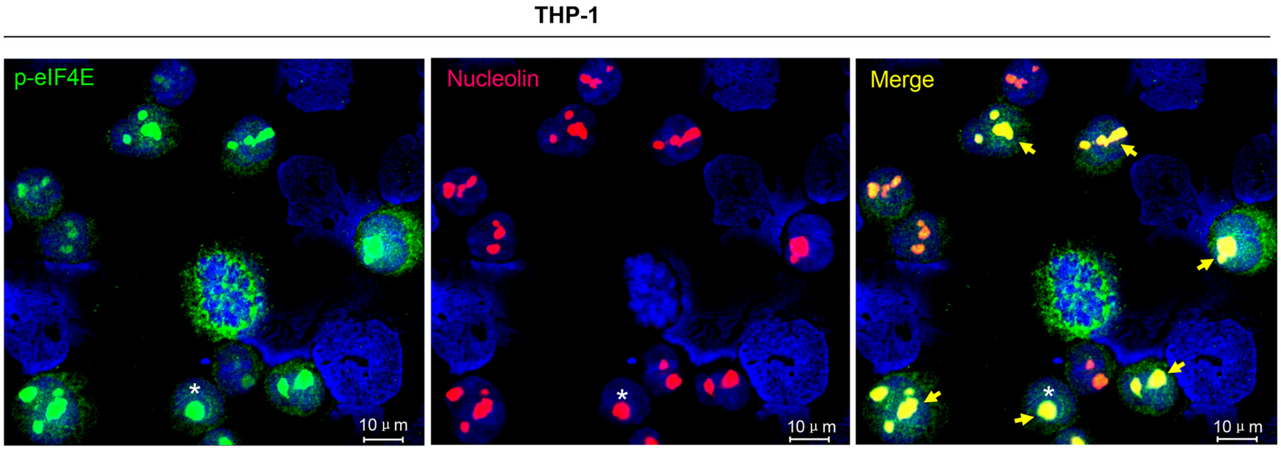

Supplementary Figure S3: Colocalization staining of the GNBs with nucleoli. Colocalization images of GNBs (green nuclear bodies) with nucleoli (red nuclear bodies) in THP-1 cell nuclei. Most GNBs colocalized with nucleoli (yellow arrows). Cell nuclei (blue) were stained with DAPI.

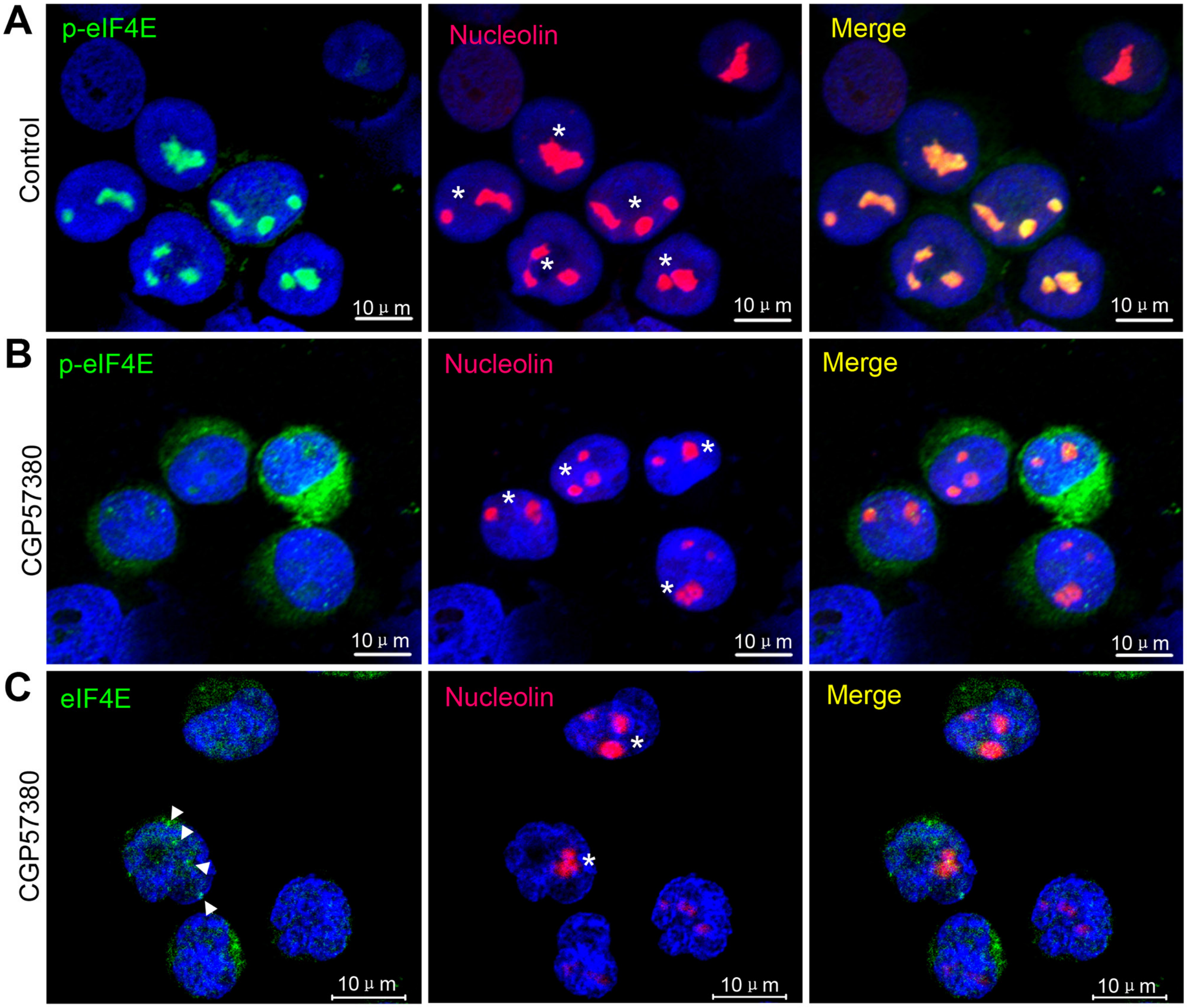

Supplementary Figure S4: CGP57380 depletes GNBs without affecting eIF4E nuclear bodies and nucleoli. A. Control: most GNBs were colocalized with nucleoli in KG-1 leukemia cells. **B.** Treatment with CGP57380 depleted GNBs (green) but did not affect nucleoli (red) in KG-1 cells. C. Treatment with CGP57380 did not affect eIF4E nuclear bodies (green dot, arrowheads) in KG-1 cells. Cell nuclei (blue) were stained with DAPI.

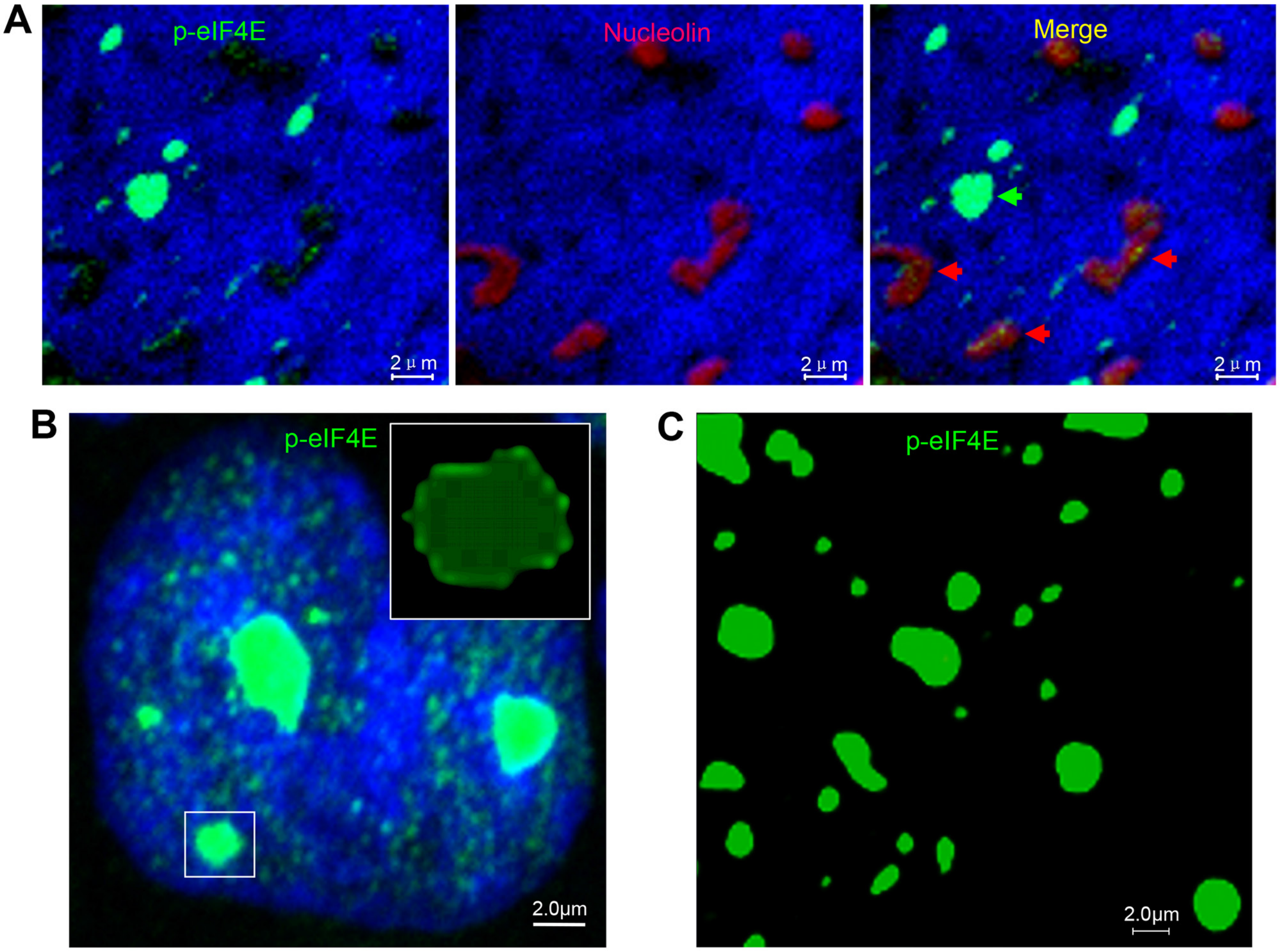

Supplementary Figure S5: Images of GNBs in the nucleus and purified GNBs. A. GNBs (green arrows) were removed completely from nucleoli (red arrows) in nuclei of KG-1 cells after treatment with 0.05% NP40 for 5 min. **B.** Confocal microscopy images of GNBs in leukemia cells. C. Images of purified GNBs from KG-1 leukemia cells. GNBs were detected with antibody against p-eIF4E and nucleoli were detected with nucleolin antibody. Cell nuclei (blue) were stained using DAPI.

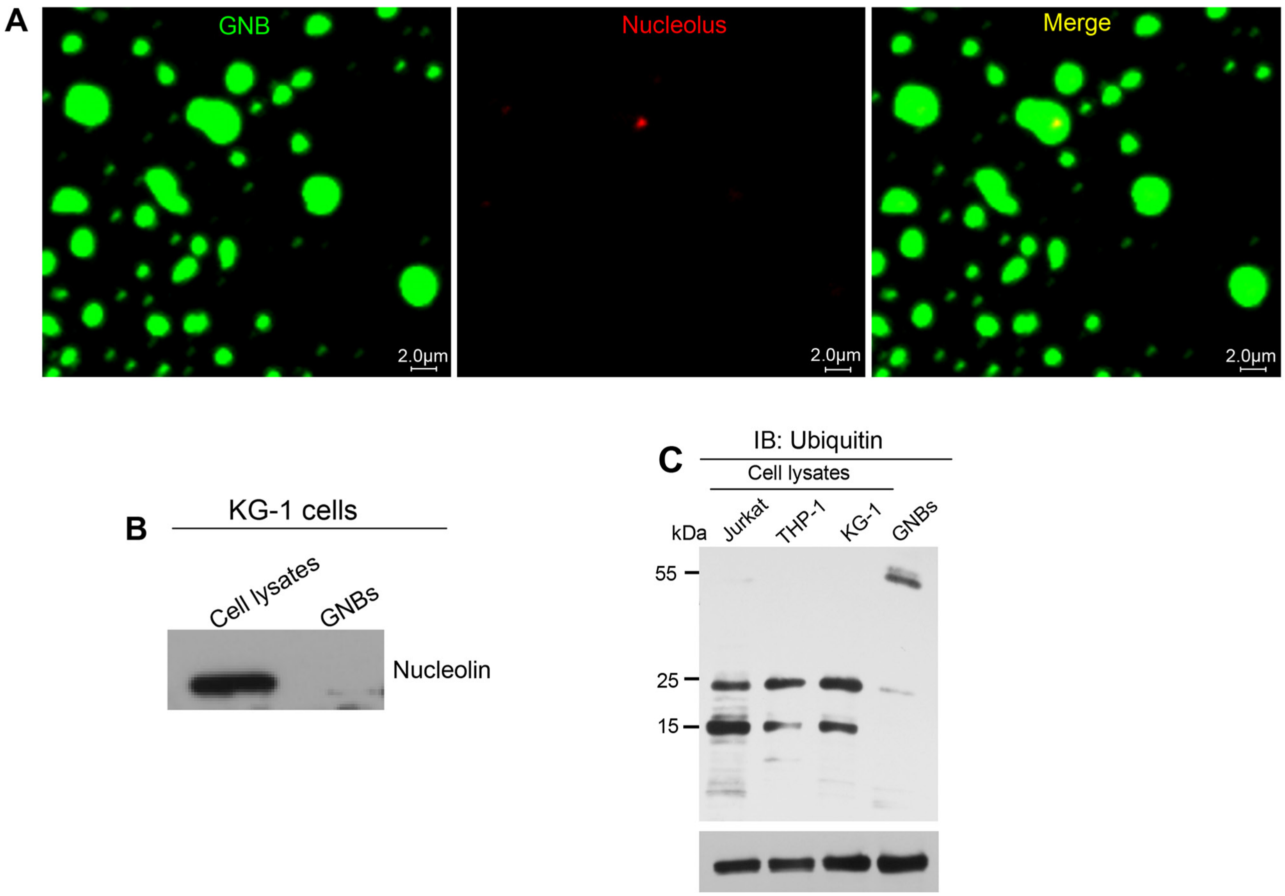

Supplementary Figure S6: GNBs are different from nucleoli in leukemia cells. A. Images of purified GNBs (Green) and nucleoli (Red) from KG-1 leukemia cells. **B.** Western blot analysis of nucleoli of GNBs from KG-1 cells. The GNBs (green) and nucleoli (red) were revealed by immunofluorescence staining with p-eIF4E antibody or nuleolin antibody. Cell nuclei (blue) were stained using DAPI. **C.** Analyses of ubiquitin-modified proteins of total cell lysates and purified GNB lysates using Western blotting with antibodies against ubiquitin.

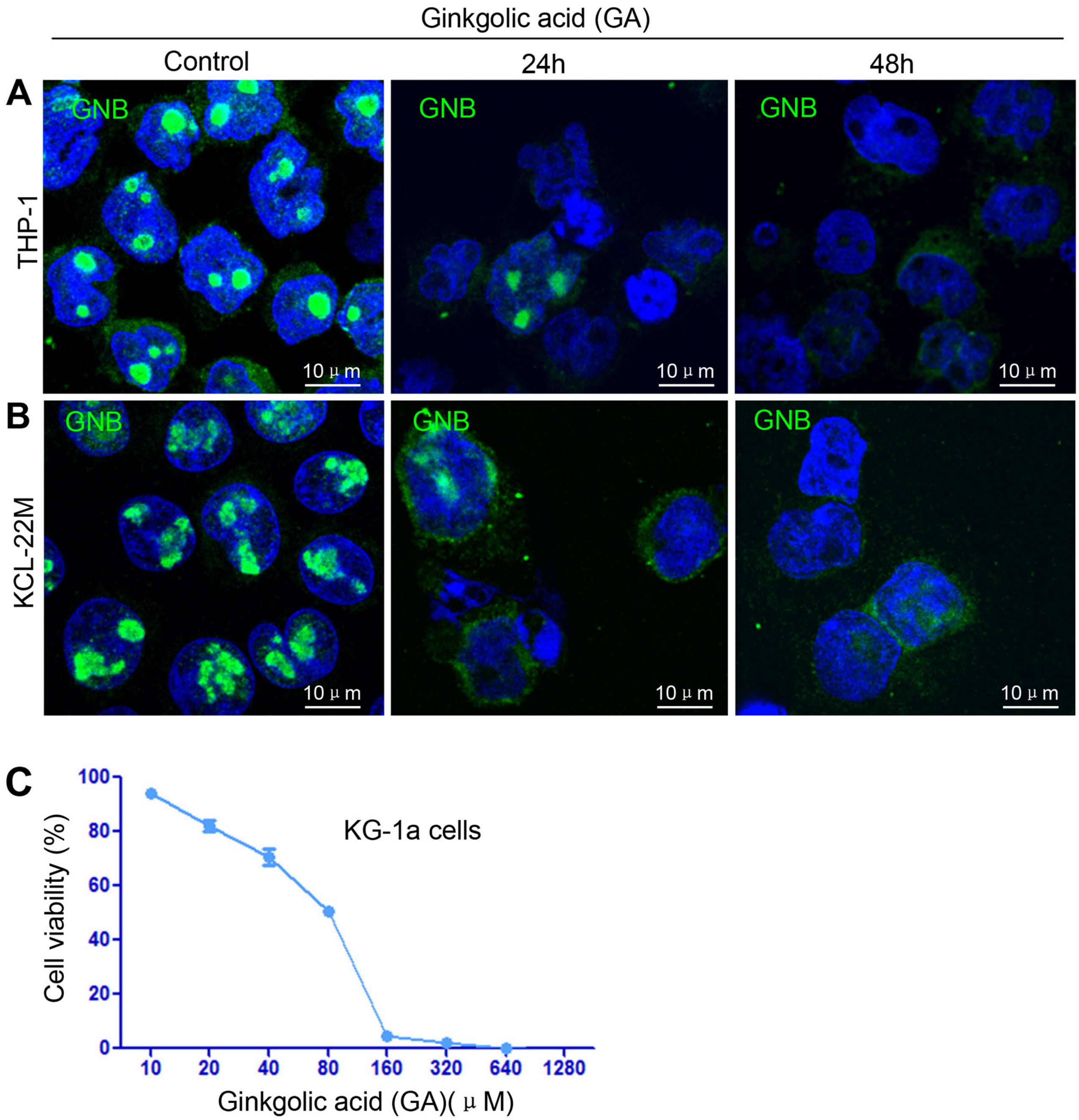

Supplementary Figure S7: SUMOylation is essential for the formation of GNBs and cell viability. A–B. Confocal images of GNBs in THP-1 and KCL-22M leukemia cells. Leukemia cells were treated with the SUMOylation inhibitor GA (200 μM) for 24 or 48 h and then immunofluorescently stained for GNBs with p-eIF4E antibody. Cell nuclei (blue) were stained with DAPI. **C.** De-sumoylation decreased cell viability with the small molecule inhibitor GA with a dose-dependent manner. KG-1a cells were treated with the indicated concentrations of GA for 48 h and then analyzed for Cell viability by MTT.

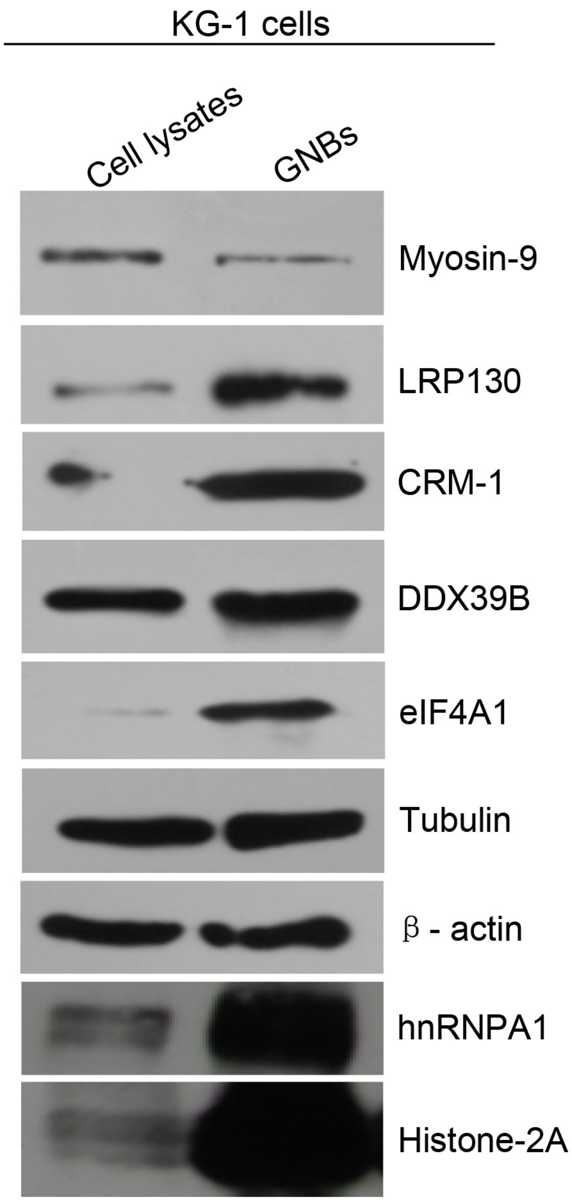

Supplementary Figure S8: Western blot analyses of a panel of representative proteins identified in GNBs. Proteins were extracted from leukemia cells and GNBs with RIPA buffer for Western blotting analyses using antibody against Myosin-9, LRP130, CRM-1, DDX39B, eIF4A1, Tubulin, β- actin, hnRNPA1 or Histone-2A.

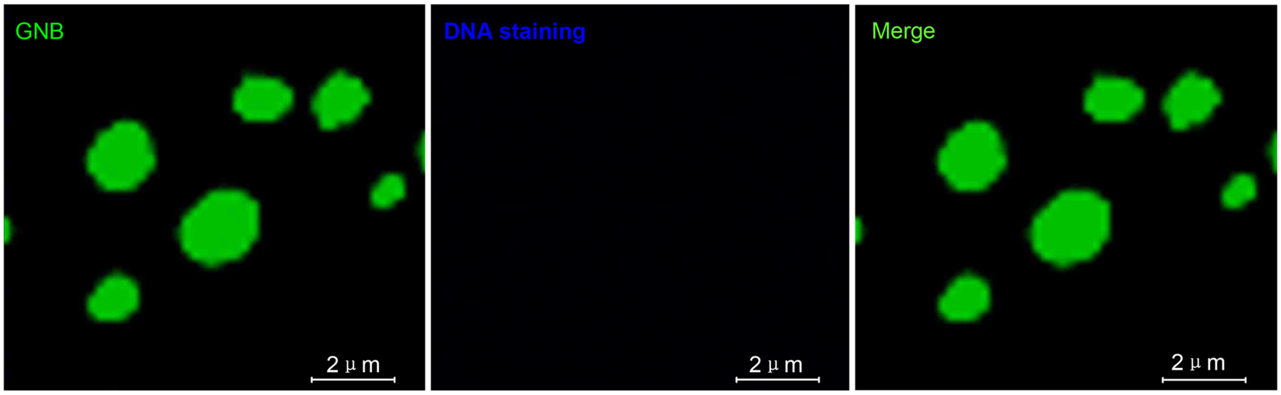

Supplementary Figure S9: DNA staining images of purified GNBs. Purified GNBs from KG-1 leukemia cells were incubated with DAPI at 1μg/ml for 10 min and then examined for DNA staining under Confocal Laser Scanning Microscope.

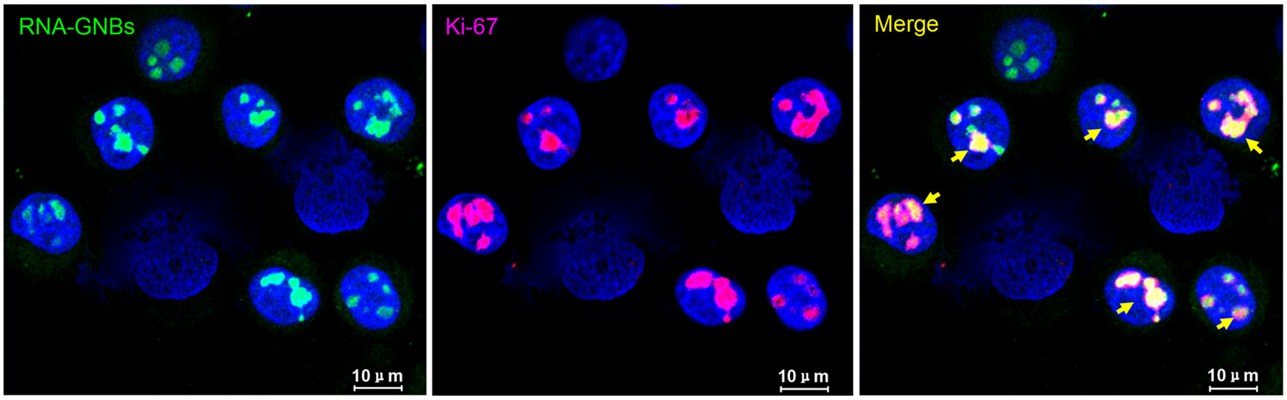

Supplementary Figure S10: Double immunofluorescence staining of GNBs and Ki-67 antigen. Synchronous analysis of the expression levels of GNBs (green nuclear bodies) and proliferating nuclear antigen Ki-67 (red) in KCL-22M leukemia cells using immunofluorescence staining.

Supplementary Table S1: Cancer cell lines, primary leukemia and normal hematopoietic cell samples for screening of the novel giant nuclear bodies

Note: +++, strong positive; ++,median positive; + weak positive; -, negative

Supplementary Table S2: Raw data of 782 different proteins identified in the 30 fragments

Supplementary Table S3: Raw data of function grouping of 782 proteins in GNBs

Supplementary Table S4: Functional classes of 782 different proteins in GNBs

No	Protein classes	proteins	%	Note
1	Ribosomal proteins	47	6.01	RNA trafficking(43.48%)
2	Molecular motor proteins	37	4.73	
3	Molecular chaperones	31	3.96	
4	Modifier proteins	29	3.71	
5	Nuclear ribonucleoproteins	26	3.32	
6	Ras-related protein Rabs	25	3.2	
7	ATP-related enzymes	22	2.81	
8	tRNA ligases	18	2.3	
9	Components of nuclear pore complex	16	2.05	
10	Histones	15	1.92	
11	GTPase-related proteins	14	1.79	
12	Eukaryotic translation initiation factors	14	1.79	
13	mRNA transport-related proteins	13	1.66	
14	Pre-mRNA splicing factors	9	1.15	
15	DNA transcription-related proteins	9	1.15	
16	ATP-dependent RNA helicases	9	1.15	
17	Elongation factors	6	0.77	
18	Enzymes	183	23.4	
19	Others	259	33.12	
Total		782	100	
