# Supplementary material for: Identification of a novel RNA giant nuclear body in cancer cells
Source: Oncotarget. 2015 Dec 15;7(4):4724–34. doi: 10.18632/oncotarget.6619 (PMC4826238; doi:10.18632/oncotarget.6619)
Supplement: Supplementary file 2 [file oncotarget-07-4724-s002.doc]

**Table S1**

Cancer cell lines, primary leukemia and normal hematopoietic cell samples for screening of the novel giant nuclear bodies

| Cell lines | | Giant nuclear bodies |
| --- | --- | --- |
| Chronic myeloid leukemia (CML) | Kcl-22 | +++ |
|  | Kcl-22M | +++ |
|  | K562 | +++ |
|  | K562/adr | +++ |
|  | KU812 | +++ |
|  | MEG-01 | +++ |
| Acute myeloid leukemia (AML) | KG-1 | +++ |
|  | THP-1 | +++ |
|  | NB4 | +++ |
|  | Kasumi-1 | +++ |
|  | U937 | +++ |
| Acute lymphoblastic leukemia (ALL) | Molt-4 | +++ |
|  | Jurkat | +++ |
| Lymphoma | Raji | +++ |
|  | Jeko-1 | +++ |
|  | Pfeffier | +++ |
| Multiple myeloma (MM) | RPMI 8226 | +++ |
|  | KM3 | +++ |
| Lung cancer | A549 | +++ |
|  | H1299 | +++ |
|  | H1975 | +++ |
|  | HCC827 | +++ |
| Colorectal cancer | RKO | +++ |
|  | SW480 | +++ |
|  | SW620 | +++ |
|  | HT29 | +++ |
|  | HCT-116 | +++ |
|  | HCT-8 | +++ |
|  | CaCo2 | +++ |
| Gastric cancer | MGC803 | +++ |
|  | SGC 7901 | +++ |
| Liver cancer | HepG2 | +++ |
|  | Huh7 | +++ |
|  | SK-Hep-1 | +++ |
| Pancreatic cancer | BxPC-3 | +++ |
|  | MIA PaCa-2 | +++ |
|  | Panc-1 | +++ |
| Breast cancer | MCF-7 | +++ |
|  | Bcap-37 | +++ |
|  | MB231 | +++ |
| Cervical cancer | Hela | +++ |
| Prostate cancer | PC-3 | +++ |
| Renal cancer | 786-0 | +++ |
|  | ACHN | +++ |
| Fibrosarcoma | HT1080 | +++ |
| chondrosarcoma | SW1353 | +++ |
| Osteosracoma | MG-63 | +++ |
|  | HOS | +++ |
| Primary leukemia cell samples | No1: CML-BC | ++ |
|  | No2: CML | ++ |
|  | No3:AML-M2 | ++ |
|  | No4:AML-M5 | ++ |
|  | No5:AML-M5 | ++ |
|  | No6:AML-M5 | ++ |
|  | No7:AML-M4 | ++ |
|  | No8:CML-BC | ++ |
|  | No9:AML-M0 | ++ |
|  | No10:AML-M2 | ++ |
|  | No11:AML-M2 | ++ |
|  | No12:AML-M2 | ++ |
|  | No13:AML-M5 | ++ |
|  | No14:AML-M5 | ++ |
|  | No15:AML-M5 | ++ |
|  | No16:CML | ++ |
|  | No17:AML-M4 | ++ |
|  | No18:AML-M4 | ++ |
|  | No19:AML | + |
|  | No20:AML-M3 | + |
|  | No21:AML-M4 | + |
|  | No22:CML-BC | + |
|  | No23:AML-M4 | + |
|  | No24:CML | + |
|  | No25:AML-M6 | + |
|  | No26:CML | + |
|  | No27:AML-M3 | + |
|  | No28:AML-M4 | + |
|  | No29:AML-M5b | + |
|  | No30:AML-M2 | + |
|  | No31:ALL-B | + |
|  | No32:ALL | + |
|  | No33:ALL | + |
|  | No34:ALL | + |
|  | No35:AML-M4 | + |
| Normal hematopoietic cell samples | No1:HSCs | - |
|  | No2:HSCs | - |
|  | No3:Cord blood | - |
|  | No4-7:Peripheral blood | - |

Note: +++, strong positive; ++,median positive; + weak positive; -, negative
