# Supplementary material for: Identification of a novel RNA giant nuclear body in cancer cells
Source: Oncotarget. 2015 Dec 15;7(4):4724–34. doi: 10.18632/oncotarget.6619 (PMC4826238; doi:10.18632/oncotarget.6619)
Supplement: Supplementary file 4 [file oncotarget-07-4724-s004.doc]

**Table S3 Raw data of function grouping of 782 proteins in GNBs**

| **Accession number** | **Protein name** | | **Score** | **MW** |
| --- | --- | --- | --- | --- |
| **(1) Ribosomal proteins** | | | |  |
| sp|P82650|RT22_HUMAN | | 28S ribosomal protein S22, mitochondrial OS=Homo sapiens GN=MRPS22 PE=1 SV=1 | 1164 | 9220 |
| sp|Q9Y3D9|RT23_HUMAN | | 28S ribosomal protein S23, mitochondrial OS=Homo sapiens GN=MRPS23 PE=1 SV=2 | 932 | 12303 |
| sp|Q9NYK5|RM39_HUMAN | | 39S ribosomal protein L39, mitochondrial OS=Homo sapiens GN=MRPL39 PE=1 SV=3 | 354 | 12947 |
| sp|Q9P015|RM15_HUMAN | | 39S ribosomal protein L15, mitochondrial OS=Homo sapiens GN=MRPL15 PE=1 SV=1 | 943 | 13478 |
| sp|Q9BYD3|RM04_HUMAN | | 39S ribosomal protein L4, mitochondrial OS=Homo sapiens GN=MRPL4 PE=1 SV=1 | 874 | 13791 |
| sp|Q9Y3B7|RM11_HUMAN | | 39S ribosomal protein L11, mitochondrial OS=Homo sapiens GN=MRPL11 PE=1 SV=1 | 469 | 14835 |
| sp|Q96A35|RM24_HUMAN | | 39S ribosomal protein L24, mitochondrial OS=Homo sapiens GN=MRPL24 PE=1 SV=1 | 263 | 14905 |
| sp|Q9BYD1|RM13_HUMAN | | 39S ribosomal protein L13, mitochondrial OS=Homo sapiens GN=MRPL13 PE=1 SV=1 | 724 | 14944 |
| sp|Q9BYC9|RM20_HUMAN | | 39S ribosomal protein L20, mitochondrial OS=Homo sapiens GN=MRPL20 PE=1 SV=1 | 621 | 15413 |
| sp|P63220|RS21_HUMAN | | 40S ribosomal protein S21 OS=Homo sapiens GN=RPS21 PE=1 SV=1 | 943 | 15597 |
| sp|P62244|RS15A_HUMAN | | 40S ribosomal protein S15a OS=Homo sapiens GN=RPS15A PE=1 SV=2 | 1661 | 15788 |
| sp|P60866|RS20_HUMAN | | 40S ribosomal protein S20 OS=Homo sapiens GN=RPS20 PE=1 SV=1 | 853 | 15795 |
| sp|P25398|RS12_HUMAN | | 40S ribosomal protein S12 OS=Homo sapiens GN=RPS12 PE=1 SV=3 | 1013 | 17212 |
| sp|P62851|RS25_HUMAN | | 40S ribosomal protein S25 OS=Homo sapiens GN=RPS25 PE=1 SV=1 | 582 | 17246 |
| sp|P62269|RS18_HUMAN | | 40S ribosomal protein S18 OS=Homo sapiens GN=RPS18 PE=1 SV=3 | 1576 | 17603 |
| sp|P62277|RS13_HUMAN | | 40S ribosomal protein S13 OS=Homo sapiens GN=RPS13 PE=1 SV=2 | 1198 | 17708 |
| sp|P0CW22|RS17L_HUMAN | | 40S ribosomal protein S17-like OS=Homo sapiens GN=RPS17L PE=1 SV=1 | 770 | 17979 |
| sp|P62847|RS24_HUMAN | | 40S ribosomal protein S24 OS=Homo sapiens GN=RPS24 PE=1 SV=1 | 704 | 18296 |
| sp|P46783|RS10_HUMAN | | 40S ribosomal protein S10 OS=Homo sapiens GN=RPS10 PE=1 SV=1 | 776 | 18590 |
| sp|P62280|RS11_HUMAN | | 40S ribosomal protein S11 OS=Homo sapiens GN=RPS11 PE=1 SV=3 | 499 | 18886 |
| sp|P46781|RS9_HUMAN | | 40S ribosomal protein S9 OS=Homo sapiens GN=RPS9 PE=1 SV=3 | 420 | 20468 |
| sp|P62081|RS7_HUMAN | | 40S ribosomal protein S7 OS=Homo sapiens GN=RPS7 PE=1 SV=1 | 832 | 20727 |
| sp|P46782|RS5_HUMAN | | 40S ribosomal protein S5 OS=Homo sapiens GN=RPS5 PE=1 SV=4 | 698 | 20736 |
| sp|P23396|RS3_HUMAN | | 40S ribosomal protein S3 OS=Homo sapiens GN=RPS3 PE=1 SV=2 | 1415 | 21611 |
| sp|P62701|RS4X_HUMAN | | 40S ribosomal protein S4, X isoform OS=Homo sapiens GN=RPS4X PE=1 SV=2 | 831 | 21814 |
| sp|P61247|RS3A_HUMAN | | 40S ribosomal protein S3a OS=Homo sapiens GN=RPS3A PE=1 SV=2 | 1305 | 21964 |
| sp|P15880|RS2_HUMAN | | 40S ribosomal protein S2 OS=Homo sapiens GN=RPS2 PE=1 SV=2 | 818 | 22113 |
| sp|Q9Y3U8|RL36_HUMAN | | 60S ribosomal protein L36 OS=Homo sapiens GN=RPL36 PE=1 SV=3 | 557 | 22635 |
| sp|P62888|RL30_HUMAN | | 60S ribosomal protein L30 OS=Homo sapiens GN=RPL30 PE=1 SV=2 | 1178 | 23033 |
| sp|P35268|RL22_HUMAN | | 60S ribosomal protein L22 OS=Homo sapiens GN=RPL22 PE=1 SV=2 | 503 | 24304 |
| sp|P61353|RL27_HUMAN | | 60S ribosomal protein L27 OS=Homo sapiens GN=RPL27 PE=1 SV=2 | 648 | 24987 |
| sp|Q9UNX3|RL26L_HUMAN | | 60S ribosomal protein L26-like 1 OS=Homo sapiens GN=RPL26L1 PE=1 SV=1 | 386 | 25013 |
| sp|P46779|RL28_HUMAN | | 60S ribosomal protein L28 OS=Homo sapiens GN=RPL28 PE=1 SV=3 | 291 | 26842 |
| sp|P30050|RL12_HUMAN | | 60S ribosomal protein L12 OS=Homo sapiens GN=RPL12 PE=1 SV=1 | 1414 | 29264 |
| sp|P62913|RL11_HUMAN | | 60S ribosomal protein L11 OS=Homo sapiens GN=RPL11 PE=1 SV=2 | 1167 | 29807 |
| sp|P18621|RL17_HUMAN | | 60S ribosomal protein L17 OS=Homo sapiens GN=RPL17 PE=1 SV=3 | 301 | 30148 |
| sp|P32969|RL9_HUMAN | | 60S ribosomal protein L9 OS=Homo sapiens GN=RPL9 PE=1 SV=1 | 588 | 30154 |
| sp|P62906|RL10A_HUMAN | | 60S ribosomal protein L10a OS=Homo sapiens GN=RPL10A PE=1 SV=2 | 990 | 31590 |
| sp|P26373|RL13_HUMAN | | 60S ribosomal protein L13 OS=Homo sapiens GN=RPL13 PE=1 SV=4 | 304 | 32765 |
| sp|P18124|RL7_HUMAN | | 60S ribosomal protein L7 OS=Homo sapiens GN=RPL7 PE=1 SV=1 | 1254 | 33570 |
| sp|P62424|RL7A_HUMAN | | 60S ribosomal protein L7a OS=Homo sapiens GN=RPL7A PE=1 SV=2 | 560 | 34423 |
| sp|Q02878|RL6_HUMAN | | 60S ribosomal protein L6 OS=Homo sapiens GN=RPL6 PE=1 SV=3 | 422 | 34569 |
| sp|P46777|RL5_HUMAN | | 60S ribosomal protein L5 OS=Homo sapiens GN=RPL5 PE=1 SV=3 | 1108 | 34954 |
| sp|P05388|RLA0_HUMAN | | 60S acidic ribosomal protein P0 OS=Homo sapiens GN=RPLP0 PE=1 SV=1 | 1496 | 39200 |
| sp|P39023|RL3_HUMAN | | 60S ribosomal protein L3 OS=Homo sapiens GN=RPL3 PE=1 SV=2 | 490 | 41425 |
| sp|P36578|RL4_HUMAN | | 60S ribosomal protein L4 OS=Homo sapiens GN=RPL4 PE=1 SV=5 | 948 | 46365 |
| sp|P62979|RS27A_HUMAN | | Ubiquitin-40S ribosomal protein S27a OS=Homo sapiens GN=RPS27A PE=1 SV=2 | 1526 | 47953 |
| **(2) Molecular motor proteins** | | | | |
| sp|P63261|ACTG_HUMAN | Actin, cytoplasmic 2 OS=Homo sapiens GN=ACTG1 PE=1 SV=1 | | 35331 | 16367 |
| sp|P60709|ACTB_HUMAN | Actin, cytoplasmic 1 OS=Homo sapiens GN=ACTB PE=1 SV=1 | | 35232 | 19768 |
| sp|P68032|ACTC_HUMAN | Actin, alpha cardiac muscle 1 OS=Homo sapiens GN=ACTC1 PE=1 SV=1 | | 10909 | 20761 |
| sp|Q562R1|ACTBL_HUMAN | Beta-actin-like protein 2 OS=Homo sapiens GN=ACTBL2 PE=1 SV=2 | | 7360 | 31616 |
| sp|O15511|ARPC5_HUMAN | Actin-related protein 2/3 complex subunit 5 OS=Homo sapiens GN=ARPC5 PE=1 SV=3 | | 945 | 33073 |
| sp|O15145|ARPC3_HUMAN | Actin-related protein 2/3 complex subunit 3 OS=Homo sapiens GN=ARPC3 PE=1 SV=3 | | 1281 | 42052 |
| sp|P61158|ARP3_HUMAN | Actin-related protein 3 OS=Homo sapiens GN=ACTR3 PE=1 SV=3 | | 1869 | 42108 |
| sp|P59998|ARPC4_HUMAN | Actin-related protein 2/3 complex subunit 4 OS=Homo sapiens GN=ARPC4 PE=1 SV=3 | | 834 | 42318 |
| sp|O60264|SMCA5_HUMAN | SWI/SNF-related matrix-associated actin-dependent regulator of chromatin subfamily A member 5 OS=Homo sapiens GN=SMARCA5 PE=1 SV=1 | | 390 | 42334 |
| sp|P52907|CAZA1_HUMAN | F-actin-capping protein subunit alpha-1 OS=Homo sapiens GN=CAPZA1 PE=1 SV=3 | | 1228 | 47797 |
| sp|P47756|CAPZB_HUMAN | F-actin-capping protein subunit beta OS=Homo sapiens GN=CAPZB PE=1 SV=4 | | 1256 | 103563 |
| sp|O43707|ACTN4_HUMAN | Alpha-actinin-4 OS=Homo sapiens GN=ACTN4 PE=1 SV=2 | | 15975 | 105245 |
| sp|P12814|ACTN1_HUMAN | Alpha-actinin-1 OS=Homo sapiens GN=ACTN1 PE=1 SV=2 | | 13650 | 122513 |
| sp|P07437|TBB5_HUMAN | Tubulin beta chain OS=Homo sapiens GN=TUBB PE=1 SV=2 | | 8309 | 50095 |
| sp|P68371|TBB4B_HUMAN | Tubulin beta-4B chain OS=Homo sapiens GN=TUBB4B PE=1 SV=1 | | 7327 | 50255 |
| sp|Q13509|TBB3_HUMAN | Tubulin beta-3 chain OS=Homo sapiens GN=TUBB3 PE=1 SV=2 | | 5157 | 50281 |
| sp|P68363|TBA1B_HUMAN | Tubulin alpha-1B chain OS=Homo sapiens GN=TUBA1B PE=1 SV=1 | | 4895 | 50548 |
| sp|Q9BQE3|TBA1C_HUMAN | Tubulin alpha-1C chain OS=Homo sapiens GN=TUBA1C PE=1 SV=1 | | 4078 | 50634 |
| sp|Q9BUF5|TBB6_HUMAN | Tubulin beta-6 chain OS=Homo sapiens GN=TUBB6 PE=1 SV=1 | | 3517 | 50804 |
| sp|P68366|TBA4A_HUMAN | Tubulin alpha-4A chain OS=Homo sapiens GN=TUBA4A PE=1 SV=1 | | 3148 | 50856 |
| sp|Q14166|TTL12_HUMAN | Tubulin--tyrosine ligase-like protein 12 OS=Homo sapiens GN=TTLL12 PE=1 SV=2 | | 545 | 75154 |
| sp|P35579|MYH9_HUMAN | Myosin-9 OS=Homo sapiens GN=MYH9 PE=1 SV=4 | | 25292 | 19824 |
| sp|Q92614|MY18A_HUMAN | Unconventional myosin-XVIIIa OS=Homo sapiens GN=MYO18A PE=1 SV=3 | | 471 | 117395 |
| sp|B0I1T2|MYO1G_HUMAN | Unconventional myosin-Ig OS=Homo sapiens GN=MYO1G PE=1 SV=2 | | 3058 | 122461 |
| sp|B0I1T2|MYO1G_HUMAN | Unconventional myosin-Ig OS=Homo sapiens GN=MYO1G PE=1 SV=2 | | 1641 | 227646 |
| sp|O00159|MYO1C_HUMAN | Unconventional myosin-Ic OS=Homo sapiens GN=MYO1C PE=1 SV=4 | | 387 | 234168 |
| sp|O14950|ML12B_HUMAN | Myosin regulatory light chain 12B OS=Homo sapiens GN=MYL12B PE=1 SV=2 | | 406 | 244846 |
| sp|P06753|TPM3_HUMAN | Tropomyosin alpha-3 chain OS=Homo sapiens GN=TPM3 PE=1 SV=2 | | 5203 | 28619 |
| sp|P67936|TPM4_HUMAN | Tropomyosin alpha-4 chain OS=Homo sapiens GN=TPM4 PE=1 SV=3 | | 4008 | 32987 |
| sp|P08670|VIME_HUMAN | Vimentin OS=Homo sapiens GN=VIM PE=1 SV=4 | | 5865 | 53676 |
| sp|P50570|DYN2_HUMAN | Dynamin-2 OS=Homo sapiens GN=DNM2 PE=1 SV=2 | | 443 | 98345 |
| sp|P23528|COF1_HUMAN | Cofilin-1 OS=Homo sapiens GN=CFL1 PE=1 SV=3 | | 651 | 18719 |
| sp|Q9Y281|COF2_HUMAN | Cofilin-2 OS=Homo sapiens GN=CFL2 PE=1 SV=1 | | 1240 | 18839 |
| sp|Q9ULV4|COR1C_HUMAN | Coronin-1C OS=Homo sapiens GN=CORO1C PE=1 SV=1 | | 966 | 51678 |
| sp|P57737|CORO7_HUMAN | Coronin-7 OS=Homo sapiens GN=CORO7 PE=1 SV=2 | | 732 | 53899 |
| sp|P31146|COR1A_HUMAN | Coronin-1A OS=Homo sapiens GN=CORO1A PE=1 SV=4 | | 1067 | 101626 |
| sp|P16949|STMN1_HUMAN | Stathmin OS=Homo sapiens GN=STMN1 PE=1 SV=3 | | 1805 | 17292 |
| **(3) Molecular chaperones** | | | | |
| sp|P61604|CH10_HUMAN | 10 kDa heat shock protein, mitochondrial OS=Homo sapiens GN=HSPE1 PE=1 SV=2 | | 4410 | 10925 |
| sp|P04792|HSPB1_HUMAN | Heat shock protein beta-1 OS=Homo sapiens GN=HSPB1 PE=1 SV=2 | | 513 | 22826 |
| sp|P08238|HS90B_HUMAN | Heat shock protein HSP 90-beta OS=Homo sapiens GN=HSP90AB1 PE=1 SV=4 | | 31278 | 41477 |
| sp|P07900|HS90A_HUMAN | Heat shock protein HSP 90-alpha OS=Homo sapiens GN=HSP90AA1 PE=1 SV=5 | | 28355 | 44953 |
| sp|P34932|HSP74_HUMAN | Heat shock 70 kDa protein 4 OS=Homo sapiens GN=HSPA4 PE=1 SV=4 | | 1214 | 61187 |
| sp|Q92598|HS105_HUMAN | Heat shock protein 105 kDa OS=Homo sapiens GN=HSPH1 PE=1 SV=1 | | 1064 | 71082 |
| sp|P10809|CH60_HUMAN | 60 kDa heat shock protein, mitochondrial OS=Homo sapiens GN=HSPD1 PE=1 SV=2 | | 5770 | 83554 |
| sp|P11142|HSP7C_HUMAN | Heat shock cognate 71 kDa protein OS=Homo sapiens GN=HSPA8 PE=1 SV=1 | | 1926 | 85006 |
| sp|O95757|HS74L_HUMAN | Heat shock 70 kDa protein 4L OS=Homo sapiens GN=HSPA4L PE=1 SV=3 | | 313 | 89684 |
| sp|Q16543|CDC37_HUMAN | Hsp90 co-chaperone Cdc37 OS=Homo sapiens GN=CDC37 PE=1 SV=1 | | 442 | 95127 |
| sp|P50502|F10A1_HUMAN | Hsc70-interacting protein OS=Homo sapiens GN=ST13 PE=1 SV=2 | | 513 | 95479 |
| sp|O75534|CSDE1_HUMAN | Cold shock domain-containing protein E1 OS=Homo sapiens GN=CSDE1 PE=1 SV=2 | | 941 | 97716 |
| sp|P17987|TCPA_HUMAN | T-complex protein 1 subunit alpha OS=Homo sapiens GN=TCP1 PE=1 SV=1 | | 2168 | 57794 |
| sp|P40227|TCPZ_HUMAN | T-complex protein 1 subunit zeta OS=Homo sapiens GN=CCT6A PE=1 SV=3 | | 1693 | 58401 |
| sp|Q99832|TCPH_HUMAN | T-complex protein 1 subunit eta OS=Homo sapiens GN=CCT7 PE=1 SV=2 | | 2285 | 58444 |
| sp|P17987|TCPA_HUMAN | T-complex protein 1 subunit alpha OS=Homo sapiens GN=TCP1 PE=1 SV=1 | | 1632 | 59842 |
| sp|P50991|TCPD_HUMAN | T-complex protein 1 subunit delta OS=Homo sapiens GN=CCT4 PE=1 SV=4 | | 1478 | 60089 |
| sp|P48643|TCPE_HUMAN | T-complex protein 1 subunit epsilon OS=Homo sapiens GN=CCT5 PE=1 SV=1 | | 1276 | 60153 |
| sp|P78371|TCPB_HUMAN | T-complex protein 1 subunit beta OS=Homo sapiens GN=CCT2 PE=1 SV=4 | | 1546 | 60819 |
| sp|P49368|TCPG_HUMAN | T-complex protein 1 subunit gamma OS=Homo sapiens GN=CCT3 PE=1 SV=4 | | 3055 | 61066 |
| sp|O95816|BAG2_HUMAN | BAG family molecular chaperone regulator 2 OS=Homo sapiens GN=BAG2 PE=1 SV=1 | | 677 | 23928 |
| sp|P38646|GRP75_HUMAN | Stress-70 protein, mitochondrial OS=Homo sapiens GN=HSPA9 PE=1 SV=2 | | 7228 | 73920 |
| sp|P31689|DNJA1_HUMAN | DnaJ homolog subfamily A member 1 OS=Homo sapiens GN=DNAJA1 PE=1 SV=2 | | 905 | 30062 |
| sp|Q13217|DNJC3_HUMAN | DnaJ homolog subfamily C member 3 OS=Homo sapiens GN=DNAJC3 PE=1 SV=1 | | 1060 | 40774 |
| sp|Q8WXX5|DNJC9_HUMAN | DnaJ homolog subfamily C member 9 OS=Homo sapiens GN=DNAJC9 PE=1 SV=1 | | 476 | 45581 |
| sp|Q9UBS4|DJB11_HUMAN | DnaJ homolog subfamily B member 11 OS=Homo sapiens GN=DNAJB11 PE=1 SV=1 | | 670 | 57203 |
| sp|Q9NVH1|DJC11_HUMAN | DnaJ homolog subfamily C member 11 OS=Homo sapiens GN=DNAJC11 PE=1 SV=2 | | 948 | 58000 |
| sp|Q99615|DNJC7_HUMAN | DnaJ homolog subfamily C member 7 OS=Homo sapiens GN=DNAJC7 PE=1 SV=2 | | 447 | 63524 |
| sp|P27824|CALX_HUMAN | Calnexin OS=Homo sapiens GN=CANX PE=1 SV=2 | | 1536 | 67982 |
| sp|Q9Y5B9|SP16H_HUMAN | FACT complex subunit SPT16 OS=Homo sapiens GN=SUPT16H PE=1 SV=1 | | 1875 | 81367 |
| sp|Q08945|SSRP1_HUMAN | FACT complex subunit SSRP1 OS=Homo sapiens GN=SSRP1 PE=1 SV=1 | | 2247 | 120409 |
| **(4) Modifier proteins** | | | |  |
| sp|P22314|UBA1_HUMAN | Ubiquitin-like modifier-activating enzyme 1 OS=Homo sapiens GN=UBA1 PE=1 SV=3 | | 357 | 118858 |
| sp|Q9NT62|ATG3_HUMAN | Ubiquitin-like-conjugating enzyme ATG3 OS=Homo sapiens GN=ATG3 PE=1 SV=1 | | 336 | 36298 |
| sp|Q5T447|HECD3_HUMAN | E3 ubiquitin-protein ligase HECTD3 OS=Homo sapiens GN=HECTD3 PE=1 SV=1 | | 151 | 98135 |
| sp|P54578|UBP14_HUMAN | Ubiquitin carboxyl-terminal hydrolase 14 OS=Homo sapiens GN=USP14 PE=1 SV=3 | | 380 | 56489 |
| sp|Q15819|UB2V2_HUMAN | Ubiquitin-conjugating enzyme E2 variant 2 OS=Homo sapiens GN=UBE2V2 PE=1 SV=4 | | 552 | 16409 |
| sp|Q13404|UB2V1_HUMAN | Ubiquitin-conjugating enzyme E2 variant 1 OS=Homo sapiens GN=UBE2V1 PE=1 SV=2 | | 545 | 16598 |
| sp|Q9Y5K5|UCHL5_HUMAN | Ubiquitin carboxyl-terminal hydrolase isozyme L5 OS=Homo sapiens GN=UCHL5 PE=1 SV=3 | | 253 | 37868 |
| sp|Q13619|CUL4A_HUMAN | Cullin-4A OS=Homo sapiens GN=CUL4A PE=1 SV=3 | | 572 | 88138 |
| sp|Q9HB71|CYBP_HUMAN | Calcyclin-binding protein OS=Homo sapiens GN=CACYBP PE=1 SV=2 | | 470 | 26308 |
| sp|Q9UL46|PSME2_HUMAN | Proteasome activator complex subunit 2 OS=Homo sapiens GN=PSME2 PE=1 SV=4 | | 739 | 27555 |
| sp|Q06323|PSME1_HUMAN | Proteasome activator complex subunit 1 OS=Homo sapiens GN=PSME1 PE=1 SV=1 | | 656 | 28876 |
| sp|P25786|PSA1_HUMAN | Proteasome subunit alpha type-1 OS=Homo sapiens GN=PSMA1 PE=1 SV=1 | | 1454 | 29822 |
| sp|P25789|PSA4_HUMAN | Proteasome subunit alpha type-4 OS=Homo sapiens GN=PSMA4 PE=1 SV=1 | | 437 | 29750 |
| sp|P25788|PSA3_HUMAN | Proteasome subunit alpha type-3 OS=Homo sapiens GN=PSMA3 PE=1 SV=2 | | 280 | 28643 |
| sp|Q15008|PSMD6_HUMAN | 26S proteasome non-ATPase regulatory subunit 6 OS=Homo sapiens GN=PSMD6 PE=1 SV=1 | | 894 | 45787 |
| sp|O00231|PSD11_HUMAN | 26S proteasome non-ATPase regulatory subunit 11 OS=Homo sapiens GN=PSMD11 PE=1 SV=3 | | 677 | 47719 |
| sp|P51665|PSMD7_HUMAN | 26S proteasome non-ATPase regulatory subunit 7 OS=Homo sapiens GN=PSMD7 PE=1 SV=2 | | 856 | 37060 |
| sp|Q99460|PSMD1_HUMAN | 26S proteasome non-ATPase regulatory subunit 1 OS=Homo sapiens GN=PSMD1 PE=1 SV=2 | | 664 | 106795 |
| sp|Q13200|PSMD2_HUMAN | 26S proteasome non-ATPase regulatory subunit 2 OS=Homo sapiens GN=PSMD2 PE=1 SV=3 | | 2572 | 100877 |
| sp|P17980|PRS6A_HUMAN | 26S protease regulatory subunit 6A OS=Homo sapiens GN=PSMC3 PE=1 SV=3 | | 399 | 49458 |
| sp|P43686|PRS6B_HUMAN | 26S protease regulatory subunit 6B OS=Homo sapiens GN=PSMC4 PE=1 SV=2 | | 391 | 47451 |
| sp|Q15418|KS6A1_HUMAN | Ribosomal protein S6 kinase alpha-1 OS=Homo sapiens GN=RPS6KA1 PE=1 SV=2 | | 502 | 83070 |
| sp|Q969S9|RRF2M_HUMAN | Ribosome-releasing factor 2, mitochondrial OS=Homo sapiens GN=GFM2 PE=1 SV=1 | | 671 | 87401 |
| sp|Q8NI36|WDR36_HUMAN | WD repeat-containing protein 36 OS=Homo sapiens GN=WDR36 PE=1 SV=1 | | 273 | 106282 |
| sp|O75083|WDR1_HUMAN | WD repeat-containing protein 1 OS=Homo sapiens GN=WDR1 PE=1 SV=4 | | 385 | 66836 |
| sp|Q8IWA0|WDR75_HUMAN | WD repeat-containing protein 75 OS=Homo sapiens GN=WDR75 PE=1 SV=1 | | 382 | 95921 |
| sp|P83916|CBX1_HUMAN | Chromobox protein homolog 1 OS=Homo sapiens GN=CBX1 PE=1 SV=1 | | 299 | 21519 |
| sp|Q13185|CBX3_HUMAN | Chromobox protein homolog 3 OS=Homo sapiens GN=CBX3 PE=1 SV=4 | | 724 | 20969 |
| sp|P63165|SUMO1_HUMAN | Small ubiquitin-related modifier 1 OS=Homo sapiens GN=SUMO1 PE=1 SV=1 | | 222 | 11607 |
| **(5) Nuclear ribonucleoproteins** | | | | |
| sp|P31943|HNRH1_HUMAN | Heterogeneous nuclear ribonucleoprotein H OS=Homo sapiens GN=HNRNPH1 PE=1 SV=4 | | 2085 | 36316 |
| sp|Q99729|ROAA_HUMAN | Heterogeneous nuclear ribonucleoprotein A/B OS=Homo sapiens GN=HNRNPAB PE=1 SV=2 | | 470 | 36960 |
| sp|P31942|HNRH3_HUMAN | Heterogeneous nuclear ribonucleoprotein H3 OS=Homo sapiens GN=HNRNPH3 PE=1 SV=2 | | 441 | 37464 |
| sp|Q99729|ROAA_HUMAN | Heterogeneous nuclear ribonucleoprotein A/B OS=Homo sapiens GN=HNRNPAB PE=1 SV=2 | | 545 | 38837 |
| sp|P22626|ROA2_HUMAN | Heterogeneous nuclear ribonucleoproteins A2/B1 OS=Homo sapiens GN=HNRNPA2B1 PE=1 SV=2 | | 893 | 39799 |
| sp|P22626|ROA2_HUMAN | Heterogeneous nuclear ribonucleoproteins A2/B1 OS=Homo sapiens GN=HNRNPA2B1 PE=1 SV=2 | | 1193 | 49484 |
| sp|P09651|ROA1_HUMAN | Heterogeneous nuclear ribonucleoprotein A1 OS=Homo sapiens GN=HNRNPA1 PE=1 SV=5 | | 747 | 49517 |
| sp|O60506|HNRPQ_HUMAN | Heterogeneous nuclear ribonucleoprotein Q OS=Homo sapiens GN=SYNCRIP PE=1 SV=2 | | 807 | 64720 |
| sp|O43390|HNRPR_HUMAN | Heterogeneous nuclear ribonucleoprotein R OS=Homo sapiens GN=HNRNPR PE=1 SV=1 | | 689 | 69788 |
| sp|Q00839|HNRPU_HUMAN | Heterogeneous nuclear ribonucleoprotein U OS=Homo sapiens GN=HNRNPU PE=1 SV=6 | | 600 | 71184 |
| sp|Q00839|HNRPU_HUMAN | Heterogeneous nuclear ribonucleoprotein U OS=Homo sapiens GN=HNRNPU PE=1 SV=6 | | 532 | 85622 |
| sp|Q1KMD3|HNRL2_HUMAN | Heterogeneous nuclear ribonucleoprotein U-like protein 2 OS=Homo sapiens GN=HNRNPUL2 PE=1 SV=1 | | 330 | 91269 |
| sp|O75643|U520_HUMAN | U5 small nuclear ribonucleoprotein 200 kDa helicase OS=Homo sapiens GN=SNRNP200 PE=1 SV=2 | | 360 | 246006 |
| sp|Q15029|U5S1_HUMAN | 116 kDa U5 small nuclear ribonucleoprotein component OS=Homo sapiens GN=EFTUD2 PE=1 SV=1 | | 736 | 110336 |
| sp|P09661|RU2A_HUMAN | U2 small nuclear ribonucleoprotein A~ OS=Homo sapiens GN=SNRPA1 PE=1 SV=2 | | 859 | 28512 |
| sp|P14678|RSMB_HUMAN | Small nuclear ribonucleoprotein-associated proteins B and B~ OS=Homo sapiens GN=SNRPB PE=1 SV=2 | | 595 | 24765 |
| sp|P63162|RSMN_HUMAN | Small nuclear ribonucleoprotein-associated protein N OS=Homo sapiens GN=SNRPN PE=1 SV=1 | | 449 | 24769 |
| sp|P62316|SMD2_HUMAN | Small nuclear ribonucleoprotein Sm D2 OS=Homo sapiens GN=SNRPD2 PE=1 SV=1 | | 685 | 13632 |
| sp|Q9Y5J1|UTP18_HUMAN | U3 small nucleolar RNA-associated protein 18 homolog OS=Homo sapiens GN=UTP18 PE=1 SV=3 | | 317 | 62421 |
| sp|Q8TED0|UTP15_HUMAN | U3 small nucleolar RNA-associated protein 15 homolog OS=Homo sapiens GN=UTP15 PE=1 SV=3 | | 329 | 58721 |
| sp|O43818|U3IP2_HUMAN | U3 small nucleolar RNA-interacting protein 2 OS=Homo sapiens GN=RRP9 PE=1 SV=1 | | 292 | 52436 |
| sp|Q15366|PCBP2_HUMAN | Poly(rC)-binding protein 2 OS=Homo sapiens GN=PCBP2 PE=1 SV=1 | | 654 | 38955 |
| sp|Q15365|PCBP1_HUMAN | Poly(rC)-binding protein 1 OS=Homo sapiens GN=PCBP1 PE=1 SV=2 | | 533 | 37987 |
| sp|Q15366|PCBP2_HUMAN | Poly(rC)-binding protein 2 OS=Homo sapiens GN=PCBP2 PE=1 SV=1 | | 595 | 38955 |
| sp|Q13310|PABP4_HUMAN | Polyadenylate-binding protein 4 OS=Homo sapiens GN=PABPC4 PE=1 SV=1 | | 1887 | 71080 |
| sp|P11940|PABP1_HUMAN | Polyadenylate-binding protein 1 OS=Homo sapiens GN=PABPC1 PE=1 SV=2 | | 1093 | 70854 |

| **Accession number** | **Protein name** | **Score** | **MW** |
| --- | --- | --- | --- |
| **(6) Ras-related protein Rab** | | | |
| sp|Q9H0U4|RAB1B_HUMAN | Ras-related protein Rab-1B OS=Homo sapiens GN=RAB1B PE=1 SV=1 | 2016 | 21040 |
| sp|P51148|RAB5C_HUMAN | Ras-related protein Rab-5C OS=Homo sapiens GN=RAB5C PE=1 SV=2 | 1787 | 21316 |
| sp|P51149|RAB7A_HUMAN | Ras-related protein Rab-7a OS=Homo sapiens GN=RAB7A PE=1 SV=1 | 1713 | 22328 |
| sp|P61026|RAB10_HUMAN | Ras-related protein Rab-10 OS=Homo sapiens GN=RAB10 PE=1 SV=1 | 987 | 22755 |
| sp|Q15907|RB11B_HUMAN | Ras-related protein Rab-11B OS=Homo sapiens GN=RAB11B PE=1 SV=4 | 931 | 22891 |
| sp|Q92930|RAB8B_HUMAN | Ras-related protein Rab-8B OS=Homo sapiens GN=RAB8B PE=1 SV=2 | 862 | 22988 |
| sp|P20339|RAB5A_HUMAN | Ras-related protein Rab-5A OS=Homo sapiens GN=RAB5A PE=1 SV=2 | 664 | 23296 |
| sp|P20340|RAB6A_HUMAN | Ras-related protein Rab-6A OS=Homo sapiens GN=RAB6A PE=1 SV=3 | 617 | 23395 |
| sp|Q15286|RAB35_HUMAN | Ras-related protein Rab-35 OS=Homo sapiens GN=RAB35 PE=1 SV=1 | 617 | 23508 |
| sp|P51153|RAB13_HUMAN | Ras-related protein Rab-13 OS=Homo sapiens GN=RAB13 PE=1 SV=1 | 429 | 23637 |
| sp|Q969Q5|RAB24_HUMAN | Ras-related protein Rab-24 OS=Homo sapiens GN=RAB24 PE=1 SV=1 | 298 | 23692 |
| sp|P10301|RRAS_HUMAN | Ras-related protein R-Ras OS=Homo sapiens GN=RRAS PE=1 SV=1 | 296 | 23696 |
| sp|P61019|RAB2A_HUMAN | Ras-related protein Rab-2A OS=Homo sapiens GN=RAB2A PE=1 SV=1 | 822 | 23702 |
| sp|Q9H0U4|RAB1B_HUMAN | Ras-related protein Rab-1B OS=Homo sapiens GN=RAB1B PE=1 SV=1 | 558 | 23723 |
| sp|P62820|RAB1A_HUMAN | Ras-related protein Rab-1A OS=Homo sapiens GN=RAB1A PE=1 SV=3 | 428 | 23740 |
| sp|P51148|RAB5C_HUMAN | Ras-related protein Rab-5C OS=Homo sapiens GN=RAB5C PE=1 SV=2 | 266 | 23760 |
| sp|P61224|RAP1B_HUMAN | Ras-related protein Rap-1b OS=Homo sapiens GN=RAP1B PE=1 SV=1 | 516 | 23824 |
| sp|P62834|RAP1A_HUMAN | Ras-related protein Rap-1A OS=Homo sapiens GN=RAP1A PE=1 SV=1 | 384 | 23872 |
| sp|P11233|RALA_HUMAN | Ras-related protein Ral-A OS=Homo sapiens GN=RALA PE=1 SV=1 | 827 | 23920 |
| sp|P51159|RB27A_HUMAN | Ras-related protein Rab-27A OS=Homo sapiens GN=RAB27A PE=1 SV=3 | 621 | 24110 |
| sp|P11234|RALB_HUMAN | Ras-related protein Ral-B OS=Homo sapiens GN=RALB PE=1 SV=1 | 569 | 24492 |
| sp|P61020|RAB5B_HUMAN | Ras-related protein Rab-5B OS=Homo sapiens GN=RAB5B PE=1 SV=1 | 379 | 24588 |
| sp|P20339|RAB5A_HUMAN | Ras-related protein Rab-5A OS=Homo sapiens GN=RAB5A PE=1 SV=2 | 368 | 25137 |
| sp|P63000|RAC1_HUMAN | Ras-related C3 botulinum toxin substrate 1 OS=Homo sapiens GN=RAC1 PE=1 SV=1 | 1024 | 21835 |
| sp|P15153|RAC2_HUMAN | Ras-related C3 botulinum toxin substrate 2 OS=Homo sapiens GN=RAC2 PE=1 SV=1 | 889 | 21814 |
| **(7) ATP-related enzymes** | | | |
| ATP synthase | |  |  |
| sp|O75947|ATP5H_HUMAN | ATP synthase subunit d, mitochondrial OS=Homo sapiens GN=ATP5H PE=1 SV=3 | 395 | 18537 |
| sp|O75964|ATP5L_HUMAN | ATP synthase subunit g, mitochondrial OS=Homo sapiens GN=ATP5L PE=1 SV=3 | 1426 | 11421 |
| sp|P48047|ATPO_HUMAN | ATP synthase subunit O, mitochondrial OS=Homo sapiens GN=ATP5O PE=1 SV=1 | 2790 | 23377 |
| sp|P06576|ATPB_HUMAN | ATP synthase subunit beta, mitochondrial OS=Homo sapiens GN=ATP5B PE=1 SV=3 | 1085 | 56525 |
| sp|P36542|ATPG_HUMAN | ATP synthase subunit gamma, mitochondrial OS=Homo sapiens GN=ATP5C1 PE=1 SV=1 | 1600 | 33032 |
| sp|P25705|ATPA_HUMAN | ATP synthase subunit alpha, mitochondrial OS=Homo sapiens GN=ATP5A1 PE=1 SV=1 | 414 | 59828 |
| sp|P18859|ATP5J_HUMAN | ATP synthase-coupling factor 6, mitochondrial OS=Homo sapiens GN=ATP5J PE=1 SV=1 | 603 | 12580 |
| ATPase |  |  |  |
| sp|P21281|VATB2_HUMAN | V-type proton ATPase subunit B, brain isoform OS=Homo sapiens GN=ATP6V1B2 PE=1 SV=3 | 1770 | 56807 |
| sp|P36543|VATE1_HUMAN | V-type proton ATPase subunit E 1 OS=Homo sapiens GN=ATP6V1E1 PE=1 SV=1 | 449 | 26186 |
| sp|P38606|VATA_HUMAN | V-type proton ATPase catalytic subunit A OS=Homo sapiens GN=ATP6V1A PE=1 SV=2 | 884 | 68660 |
| sp|Q9NTK5|OLA1_HUMAN | Obg-like ATPase 1 OS=Homo sapiens GN=OLA1 PE=1 SV=2 | 347 | 44943 |
| sp|P55072|TERA_HUMAN | Transitional endoplasmic reticulum ATPase OS=Homo sapiens GN=VCP PE=1 SV=4 | 2123 | 89950 |
| sp|P05023|AT1A1_HUMAN | Sodium/potassium-transporting ATPase subunit alpha-1 OS=Homo sapiens GN=ATP1A1 PE=1 SV=1 | 1100 | 114135 |
| sp|P54709|AT1B3_HUMAN | Sodium/potassium-transporting ATPase subunit beta-3 OS=Homo sapiens GN=ATP1B3 PE=1 SV=1 | 563 | 31834 |
| sp|P16615|AT2A2_HUMAN | Sarcoplasmic/endoplasmic reticulum calcium ATPase 2 OS=Homo sapiens GN=ATP2A2 PE=1 SV=1 | 720 | 116336 |
| sp|Q93084|AT2A3_HUMAN | Sarcoplasmic/endoplasmic reticulum calcium ATPase 3 OS=Homo sapiens GN=ATP2A3 PE=1 SV=2 | 968 | 115444 |
| sp|P22392|NDKB_HUMAN | Nucleoside diphosphate kinase B OS=Homo sapiens GN=NME2 PE=1 SV=1 | 995 | 17401 |
| sp|O60361|NDK8_HUMAN | Putative nucleoside diphosphate kinase OS=Homo sapiens GN=NME2P1 PE=5 SV=1 | 1813 | 15690 |
| sp|Q13232|NDK3_HUMAN | Nucleoside diphosphate kinase 3 OS=Homo sapiens GN=NME3 PE=1 SV=2 | 383 | 19231 |
| sp|P14618|KPYM_HUMAN | Pyruvate kinase PKM OS=Homo sapiens GN=PKM PE=1 SV=4 | 6740 | 58470 |
| sp|Q9Y230|RUVB2_HUMAN | RuvB-like 2 OS=Homo sapiens GN=RUVBL2 PE=1 SV=3 | 2224 | 51296 |
| sp|Q9Y265|RUVB1_HUMAN | RuvB-like 1 OS=Homo sapiens GN=RUVBL1 PE=1 SV=1 | 1848 | 50538 |
| **(8) tRNA ligase** | |  |  |
| sp|P07814|SYEP_HUMAN | Bifunctional glutamate/proline--tRNA ligase OS=Homo sapiens GN=EPRS PE=1 SV=5 | 1346 | 53474 |
| sp|P49588|SYAC_HUMAN | Alanine--tRNA ligase, cytoplasmic OS=Homo sapiens GN=AARS PE=1 SV=2 | 1641 | 59448 |
| sp|P54577|SYYC_HUMAN | Tyrosine--tRNA ligase, cytoplasmic OS=Homo sapiens GN=YARS PE=1 SV=4 | 577 | 63758 |
| sp|P56192|SYMC_HUMAN | Methionine--tRNA ligase, cytoplasmic OS=Homo sapiens GN=MARS PE=1 SV=2 | 708 | 66701 |
| sp|Q5JTZ9|SYAM_HUMAN | Alanine--tRNA ligase, mitochondrial OS=Homo sapiens GN=AARS2 PE=1 SV=1 | 302 | 68461 |
| sp|P47897|SYQ_HUMAN | Glutamine--tRNA ligase OS=Homo sapiens GN=QARS PE=1 SV=1 | 232 | 68461 |
| sp|Q9NSE4|SYIM_HUMAN | Isoleucine--tRNA ligase, mitochondrial OS=Homo sapiens GN=IARS2 PE=1 SV=2 | 3834 | 81841 |
| sp|P47897|SYQ_HUMAN | Glutamine--tRNA ligase OS=Homo sapiens GN=QARS PE=1 SV=1 | 739 | 84294 |
| sp|P26640|SYVC_HUMAN | Valine--tRNA ligase OS=Homo sapiens GN=VARS PE=1 SV=4 | 1223 | 88655 |
| sp|P26639|SYTC_HUMAN | Threonine--tRNA ligase, cytoplasmic OS=Homo sapiens GN=TARS PE=1 SV=3 | 1988 | 88655 |
| sp|P41252|SYIC_HUMAN | Isoleucine--tRNA ligase, cytoplasmic OS=Homo sapiens GN=IARS PE=1 SV=2 | 493 | 102249 |
| sp|Q5JTZ9|SYAM_HUMAN | Alanine--tRNA ligase, mitochondrial OS=Homo sapiens GN=AARS2 PE=1 SV=1 | 1935 | 107484 |
| sp|Q9NSD9|SYFB_HUMAN | Phenylalanine--tRNA ligase beta subunit OS=Homo sapiens GN=FARSB PE=1 SV=3 | 269 | 108299 |
| sp|Q15046|SYK_HUMAN | Lysine--tRNA ligase OS=Homo sapiens GN=KARS PE=1 SV=3 | 952 | 108299 |
| sp|O43776|SYNC_HUMAN | Asparagine--tRNA ligase, cytoplasmic OS=Homo sapiens GN=NARS PE=1 SV=1 | 537 | 114688 |
| sp|Q9BW92|SYTM_HUMAN | Threonine--tRNA ligase, mitochondrial OS=Homo sapiens GN=TARS2 PE=1 SV=1 | 1676 | 141642 |
| sp|Q15046|SYK_HUMAN | Lysine--tRNA ligase OS=Homo sapiens GN=KARS PE=1 SV=3 | 387 | 145718 |
| sp|P23381|SYWC_HUMAN | Tryptophan--tRNA ligase, cytoplasmic OS=Homo sapiens GN=WARS PE=1 SV=2 | 947 | 172080 |
| **(9) Components of nuclear pore complex** | | | |
| sp|Q8TEM1|PO210_HUMAN | Nuclear pore membrane glycoprotein 210 OS=Homo sapiens GN=NUP210 PE=1 SV=3 | 6344 | 37140 |
| sp|Q92621|NU205_HUMAN | Nuclear pore complex protein Nup205 OS=Homo sapiens GN=NUP205 PE=1 SV=3 | 876 | 42581 |
| sp|Q8TEM1|PO210_HUMAN | Nuclear pore membrane glycoprotein 210 OS=Homo sapiens GN=NUP210 PE=1 SV=3 | 1104 | 55515 |
| sp|O75694|NU155_HUMAN | Nuclear pore complex protein Nup155 OS=Homo sapiens GN=NUP155 PE=1 SV=1 | 543 | 80356 |
| sp|Q5SRE5|NU188_HUMAN | Nucleoporin NUP188 homolog OS=Homo sapiens GN=NUP188 PE=1 SV=1 | 365 | 84629 |
| sp|Q8WUM0|NU133_HUMAN | Nuclear pore complex protein Nup133 OS=Homo sapiens GN=NUP133 PE=1 SV=2 | 1288 | 93943 |
| sp|O75694|NU155_HUMAN | Nuclear pore complex protein Nup155 OS=Homo sapiens GN=NUP155 PE=1 SV=1 | 1075 | 107048 |
| sp|P57740|NU107_HUMAN | Nuclear pore complex protein Nup107 OS=Homo sapiens GN=NUP107 PE=1 SV=1 | 944 | 129924 |
| sp|P57740|NU107_HUMAN | Nuclear pore complex protein Nup107 OS=Homo sapiens GN=NUP107 PE=1 SV=1 | 492 | 156697 |
| sp|Q99567|NUP88_HUMAN | Nuclear pore complex protein Nup88 OS=Homo sapiens GN=NUP88 PE=1 SV=2 | 856 | 164355 |
| sp|Q8NFH3|NUP43_HUMAN | Nucleoporin Nup43 OS=Homo sapiens GN=NUP43 PE=1 SV=1 | 431 | 198369 |
| sp|Q8NFH4|NUP37_HUMAN | Nucleoporin Nup37 OS=Homo sapiens GN=NUP37 PE=1 SV=1 | 648 | 205895 |
| sp|Q7Z3B4|NUP54_HUMAN | Nucleoporin p54 OS=Homo sapiens GN=NUP54 PE=1 SV=2 | 994 | 230171 |
| sp|P55209|NP1L1_HUMAN | Nucleosome assembly protein 1-like 1 OS=Homo sapiens GN=NAP1L1 PE=1 SV=1 | 439 | 45631 |
| sp|P20700|LMNB1_HUMAN | Lamin-B1 OS=Homo sapiens GN=LMNB1 PE=1 SV=2 | 7113 | 66653 |
| sp|Q03252|LMNB2_HUMAN | Lamin-B2 OS=Homo sapiens GN=LMNB2 PE=1 SV=3 | 2959 | 67762 |
| **(10) Histones** | |  |  |
| sp|P62805|H4_HUMAN | Histone H4 OS=Homo sapiens GN=HIST1H4A PE=1 SV=2 | 9124 | 11360 |
| sp|Q5QNW6|H2B2F_HUMAN | Histone H2B type 2-F OS=Homo sapiens GN=HIST2H2BF PE=1 SV=3 | 6527 | 13912 |
| sp|O60814|H2B1K_HUMAN | Histone H2B type 1-K OS=Homo sapiens GN=HIST1H2BK PE=1 SV=3 | 6122 | 13882 |
| sp|P0C0S8|H2A1_HUMAN | Histone H2A type 1 OS=Homo sapiens GN=HIST1H2AG PE=1 SV=2 | 3994 | 14083 |
| sp|Q6FI13|H2A2A_HUMAN | Histone H2A type 2-A OS=Homo sapiens GN=HIST2H2AA3 PE=1 SV=3 | 3972 | 14087 |
| sp|Q7L7L0|H2A3_HUMAN | Histone H2A type 3 OS=Homo sapiens GN=HIST3H2A PE=1 SV=3 | 3034 | 14113 |
| sp|P0C0S5|H2AZ_HUMAN | Histone H2A.Z OS=Homo sapiens GN=H2AFZ PE=1 SV=2 | 1808 | 13545 |
| sp|P16104|H2AX_HUMAN | Histone H2AX OS=Homo sapiens GN=H2AFX PE=1 SV=2 | 6548 | 15135 |
| sp|Q8IUE6|H2A2B_HUMAN | Histone H2A type 2-B OS=Homo sapiens GN=HIST2H2AB PE=1 SV=3 | 3256 | 13987 |
| sp|Q71DI3|H32_HUMAN | Histone H3.2 OS=Homo sapiens GN=HIST2H3A PE=1 SV=3 | 3417 | 15436 |
| sp|Q96QV6|H2A1A_HUMAN | Histone H2A type 1-A OS=Homo sapiens GN=HIST1H2AA PE=1 SV=3 | 1284 | 14225 |
| sp|P10412|H14_HUMAN | Histone H1.4 OS=Homo sapiens GN=HIST1H1E PE=1 SV=2 | 3052 | 21852 |
| sp|P16402|H13_HUMAN | Histone H1.3 OS=Homo sapiens GN=HIST1H1D PE=1 SV=2 | 3016 | 22336 |
| sp|P16401|H15_HUMAN | Histone H1.5 OS=Homo sapiens GN=HIST1H1B PE=1 SV=3 | 2006 | 22566 |
| sp|O75367|H2AY_HUMAN | Core histone macro-H2A.1 OS=Homo sapiens GN=H2AFY PE=1 SV=4 | 3819 | 39764 |
| **(11) GTPase-related protein** | | | |
| sp|Q86VI3|IQGA3_HUMAN | Ras GTPase-activating-like protein IQGAP3 OS=Homo sapiens GN=IQGAP3 PE=1 SV=2 | 240 | 185268 |
| sp|Q13576|IQGA2_HUMAN | Ras GTPase-activating-like protein IQGAP2 OS=Homo sapiens GN=IQGAP2 PE=1 SV=4 | 227 | 181036 |
| sp|P46940|IQGA1_HUMAN | Ras GTPase-activating-like protein IQGAP1 OS=Homo sapiens GN=IQGAP1 PE=1 SV=1 | 5167 | 189761 |
| sp|Q14644|RASA3_HUMAN | Ras GTPase-activating protein 3 OS=Homo sapiens GN=RASA3 PE=1 SV=3 | 200 | 96836 |
| sp|C9J798|RAS4B_HUMAN | Putative Ras GTPase-activating protein 4B OS=Homo sapiens GN=RASA4B PE=5 SV=2 | 198 | 91376 |
| sp|Q07960|RHG01_HUMAN | Rho GTPase-activating protein 1 OS=Homo sapiens GN=ARHGAP1 PE=1 SV=1 | 334 | 50461 |
| sp|P46060|RAGP1_HUMAN | Ran GTPase-activating protein 1 OS=Homo sapiens GN=RANGAP1 PE=1 SV=1 | 705 | 63958 |
| sp|P01111|RASN_HUMAN | GTPase NRas OS=Homo sapiens GN=NRAS PE=1 SV=1 | 550 | 21501 |
| sp|P62826|RAN_HUMAN | GTP-binding nuclear protein Ran OS=Homo sapiens GN=RAN PE=1 SV=3 | 489 | 24579 |
| sp|P43487|RANG_HUMAN | Ran-specific GTPase-activating protein OS=Homo sapiens GN=RANBP1 PE=1 SV=1 | 459 | 23467 |
| sp|Q9NVA2|SEP11_HUMAN | Septin-11 OS=Homo sapiens GN=SEPT11 PE=1 SV=3 | 661 | 49652 |
| sp|Q15019|SEPT2_HUMAN | Septin-2 OS=Homo sapiens GN=SEPT2 PE=1 SV=1 | 477 | 41689 |
| sp|Q14141|SEPT6_HUMAN | Septin-6 OS=Homo sapiens GN=SEPT6 PE=1 SV=4 | 396 | 50084 |
| sp|Q9UHD8|SEPT9_HUMAN | Septin-9 OS=Homo sapiens GN=SEPT9 PE=1 SV=2 | 263 | 65646 |
| **(12) Eukaryotic translation initiation factors** | | | |
| sp|O15371|EIF3D_HUMAN | Eukaryotic translation initiation factor 3 subunit D OS=Homo sapiens GN=EIF3D PE=1 SV=1 | 502 | 17049 |
| sp|Q9BY44|EIF2A_HUMAN | Eukaryotic translation initiation factor 2A OS=Homo sapiens GN=EIF2A PE=1 SV=3 | 379 | 36374 |
| sp|Q9Y262|EIF3L_HUMAN | Eukaryotic translation initiation factor 3 subunit L OS=Homo sapiens GN=EIF3L PE=1 SV=1 | 529 | 36878 |
| sp|B5ME19|EIFCL_HUMAN | Eukaryotic translation initiation factor 3 subunit C-like protein OS=Homo sapiens GN=EIF3CL PE=2 SV=1 | 298 | 46353 |
| sp|P60842|IF4A1_HUMAN | Eukaryotic initiation factor 4A-I OS=Homo sapiens GN=EIF4A1 PE=1 SV=1 | 1914 | 46601 |
| sp|P38919|IF4A3_HUMAN | Eukaryotic initiation factor 4A-III OS=Homo sapiens GN=EIF4A3 PE=1 SV=4 | 1570 | 47126 |
| sp|P41091|IF2G_HUMAN | Eukaryotic translation initiation factor 2 subunit 3 OS=Homo sapiens GN=EIF2S3 PE=1 SV=3 | 931 | 51647 |
| sp|Q14240|IF4A2_HUMAN | Eukaryotic initiation factor 4A-II OS=Homo sapiens GN=EIF4A2 PE=1 SV=2 | 911 | 64560 |
| sp|P60842|IF4A1_HUMAN | Eukaryotic initiation factor 4A-I OS=Homo sapiens GN=EIF4A1 PE=1 SV=1 | 924 | 65519 |
| sp|Q13347|EIF3I_HUMAN | Eukaryotic translation initiation factor 3 subunit I OS=Homo sapiens GN=EIF3I PE=1 SV=1 | 542 | 66912 |
| sp|P05198|IF2A_HUMAN | Eukaryotic translation initiation factor 2 subunit 1 OS=Homo sapiens GN=EIF2S1 PE=1 SV=3 | 1458 | 102810 |
| sp|P63241|IF5A1_HUMAN | Eukaryotic translation initiation factor 5A-1 OS=Homo sapiens GN=EIF5A PE=1 SV=2 | 658 | 106091 |
| sp|P06730|IF4E_HUMAN | Eukaryotic translation initiation factor 4E OS=Homo sapiens GN=EIF4E PE=1 SV=2 | 159 | 25310 |
| sp|P46199|IF2M_HUMAN | Translation initiation factor IF-2, mitochondrial OS=Homo sapiens GN=MTIF2 PE=1 SV=2 | 410 | 81837 |
| **(13) mRNA transport-related proteins** | | | |
| sp|P42704|LPPRC_HUMAN | Leucine-rich PPR motif-containing protein, mitochondrial OS=Homo sapiens GN=LRPPRC PE=1 SV=3 | 8092 | 159003 |
| sp|Q5JTH9|RRP12_HUMAN | RRP12-like protein OS=Homo sapiens GN=RRP12 PE=1 SV=2 | 284 | 145037 |
| sp|Q8TDW0|LRC8C_HUMAN | Leucine-rich repeat-containing protein 8C OS=Homo sapiens GN=LRRC8C PE=1 SV=2 | 494 | 93359 |
| sp|Q96AG4|LRC59_HUMAN | Leucine-rich repeat-containing protein 59 OS=Homo sapiens GN=LRRC59 PE=1 SV=1 | 454 | 35308 |
| sp|O00410|IPO5_HUMAN | Importin-5 OS=Homo sapiens GN=IPO5 PE=1 SV=4 | 183 | 125032 |
| sp|Q14974|IMB1_HUMAN | Importin subunit beta-1 OS=Homo sapiens GN=KPNB1 PE=1 SV=2 | 4317 | 98420 |
| sp|P52292|IMA1_HUMAN | Importin subunit alpha-1 OS=Homo sapiens GN=KPNA2 PE=1 SV=1 | 1885 | 58168 |
| sp|O14980|XPO1_HUMAN | Exportin-1 OS=Homo sapiens GN=XPO1 PE=1 SV=1 | 333 | 124447 |
| sp|P55060|XPO2_HUMAN | Exportin-2 OS=Homo sapiens GN=CSE1L PE=1 SV=3 | 530 | 111145 |
| sp|Q92973|TNPO1_HUMAN | Transportin-1 OS=Homo sapiens GN=TNPO1 PE=1 SV=2 | 1033 | 103771 |
| sp|Q9Y5L0|TNPO3_HUMAN | Transportin-3 OS=Homo sapiens GN=TNPO3 PE=1 SV=3 | 378 | 105961 |
| sp|P39687|AN32A_HUMAN | Acidic leucine-rich nuclear phosphoprotein 32 family member A OS=Homo sapiens GN=ANP32A PE=1 SV=1 | 465 | 28682 |
| sp|Q92688|AN32B_HUMAN | Acidic leucine-rich nuclear phosphoprotein 32 family member B OS=Homo sapiens GN=ANP32B PE=1 SV=1 | 301 | 28941 |
| **(14) Pre-mRNA splicing factors** | | | |
| Pre-mRNA-processing factors | | | |
| sp|O94906|PRP6_HUMAN | Pre-mRNA-processing factor 6 OS=Homo sapiens GN=PRPF6 PE=1 SV=1 | 300 | 107656 |
| sp|O75400|PR40A_HUMAN | Pre-mRNA-processing factor 40 homolog A OS=Homo sapiens GN=PRPF40A PE=1 SV=2 | 284 | 109022 |
| sp|Q9UMS4|PRP19_HUMAN | Pre-mRNA-processing factor 19 OS=Homo sapiens GN=PRPF19 PE=1 SV=1 | 837 | 55603 |
| Splicing factors | |  |  |
| sp|Q07955|SRSF1_HUMAN | Serine/arginine-rich splicing factor 1 OS=Homo sapiens GN=SRSF1 PE=1 SV=2 | 527 | 27842 |
| sp|P84103|SRSF3_HUMAN | Serine/arginine-rich splicing factor 3 OS=Homo sapiens GN=SRSF3 PE=1 SV=1 | 722 | 19546 |
| sp|Q13242|SRSF9_HUMAN | Serine/arginine-rich splicing factor 9 OS=Homo sapiens GN=SRSF9 PE=1 SV=1 | 572 | 25640 |
| sp|Q12874|SF3A3_HUMAN | Splicing factor 3A subunit 3 OS=Homo sapiens GN=SF3A3 PE=1 SV=1 | 542 | 59154 |
| sp|Q15459|SF3A1_HUMAN | Splicing factor 3A subunit 1 OS=Homo sapiens GN=SF3A1 PE=1 SV=1 | 825 | 88888 |
| sp|Q15393|SF3B3_HUMAN | Splicing factor 3B subunit 3 OS=Homo sapiens GN=SF3B3 PE=1 SV=4 | 1825 | 136575 |
| **(15) DNA transcription-related proteins** | | | |
| Prohibitin |  |  |  |
| sp|Q99623|PHB2_HUMAN | Prohibitin-2 OS=Homo sapiens GN=PHB2 PE=1 SV=2 | 2632 | 33276 |
| sp|P35232|PHB_HUMAN | Prohibitin OS=Homo sapiens GN=PHB PE=1 SV=1 | 3845 | 29843 |
| DNA-directed RNA polymerase II | | | |
| sp|P24928|RPB1_HUMAN | DNA-directed RNA polymerase II subunit RPB1 OS=Homo sapiens GN=POLR2A PE=1 SV=2 | 867 | 218408 |
| sp|P30876|RPB2_HUMAN | DNA-directed RNA polymerase II subunit RPB2 OS=Homo sapiens GN=POLR2B PE=1 SV=1 | 371 | 135236 |
| sp|O00411|RPOM_HUMAN | DNA-directed RNA polymerase, mitochondrial OS=Homo sapiens GN=POLRMT PE=1 SV=2 | 125 | 140300 |
| sp|O15160|RPAC1_HUMAN | DNA-directed RNA polymerases I and III subunit RPAC1 OS=Homo sapiens GN=POLR1C PE=1 SV=1 | 345 | 39453 |
| sp|P53999|TCP4_HUMAN | Activated RNA polymerase II transcriptional coactivator p15 OS=Homo sapiens GN=SUB1 PE=1 SV=3 | 620 | 14386 |
| sp|Q92900|RENT1_HUMAN | Regulator of nonsense transcripts 1 OS=Homo sapiens GN=UPF1 PE=1 SV=2 | 478 | 125578 |
| sp|P17480|UBF1_HUMAN | Nucleolar transcription factor 1 OS=Homo sapiens GN=UBTF PE=1 SV=1 | 380 | 89692 |
| **(16) ATP-dependent RNA helicase** | | | |
| sp|Q08211|DHX9_HUMAN | ATP-dependent RNA helicase A OS=Homo sapiens GN=DHX9 PE=1 SV=4 | 246 | 142181 |
| sp|Q9NY93|DDX56_HUMAN | Probable ATP-dependent RNA helicase DDX56 OS=Homo sapiens GN=DDX56 PE=1 SV=1 | 353 | 62007 |
| sp|Q9NUL7|DDX28_HUMAN | Probable ATP-dependent RNA helicase DDX28 OS=Homo sapiens GN=DDX28 PE=1 SV=2 | 322 | 59773 |
| sp|Q8IYB8|SUV3_HUMAN | ATP-dependent RNA helicase SUPV3L1, mitochondrial OS=Homo sapiens GN=SUPV3L1 PE=1 SV=1 | 1369 | 88791 |
| sp|O00571|DDX3X_HUMAN | ATP-dependent RNA helicase DDX3X OS=Homo sapiens GN=DDX3X PE=1 SV=3 | 607 | 73597 |
| sp|O15523|DDX3Y_HUMAN | ATP-dependent RNA helicase DDX3Y OS=Homo sapiens GN=DDX3Y PE=1 SV=2 | 486 | 73564 |
| sp|O43143|DHX15_HUMAN | Putative pre-mRNA-splicing factor ATP-dependent RNA helicase DHX15 OS=Homo sapiens GN=DHX15 PE=1 SV=2 | 1050 | 91673 |
| sp|Q7L2E3|DHX30_HUMAN | Putative ATP-dependent RNA helicase DHX30 OS=Homo sapiens GN=DHX30 PE=1 SV=1 | 533 | 134938 |
| sp|Q13838|DX39B_HUMAN | Spliceosome RNA helicase DDX39B OS=Homo sapiens GN=DDX39B PE=1 SV=1 | 433 | 49416 |
| **(17) Elongation factors** | | |  |
| sp|P13639|EF2_HUMAN | Elongation factor 2 OS=Homo sapiens GN=EEF2 PE=1 SV=4 | 5154 | 96246 |
| sp|Q96RP9|EFGM_HUMAN | Elongation factor G, mitochondrial OS=Homo sapiens GN=GFM1 PE=1 SV=2 | 2290 | 84103 |
| sp|Q5VTE0|EF1A3_HUMAN | Putative elongation factor 1-alpha-like 3 OS=Homo sapiens GN=EEF1A1P5 PE=5 SV=1 | 575 | 50495 |
| sp|P49411|EFTU_HUMAN | Elongation factor Tu, mitochondrial OS=Homo sapiens GN=TUFM PE=1 SV=2 | 3626 | 49852 |
| sp|P29692|EF1D_HUMAN | Elongation factor 1-delta OS=Homo sapiens GN=EEF1D PE=1 SV=5 | 1063 | 31217 |
| sp|P24534|EF1B_HUMAN | Elongation factor 1-beta OS=Homo sapiens GN=EEF1B2 PE=1 SV=3 | 349 | 24919 |

| **Accession number** | **Protein name** | **Score** | | **MW** |
| --- | --- | --- | --- | --- |
| **(18) Enzymes** | |  | |  |
| Kinase |  |  | |  |
| sp|O94804|STK10_HUMAN | Serine/threonine-protein kinase 10 OS=Homo sapiens GN=STK10 PE=1 SV=1 | 799 | | 1 12749 |
| sp|Q13177|PAK2_HUMAN | Serine/threonine-protein kinase PAK 2 OS=Homo sapiens GN=PAK2 PE=1 SV=3 | 651 | | 58291 |
| sp|Q13043|STK4_HUMAN | Serine/threonine-protein kinase 4 OS=Homo sapiens GN=STK4 PE=1 SV=2 | 228 | | 55823 |
| sp|Q9P289|MST4_HUMAN | Serine/threonine-protein kinase MST4 OS=Homo sapiens GN=MST4 PE=1 SV=2 | 548 | | 46785 |
| sp|Q9UHD2|TBK1_HUMAN | Serine/threonine-protein kinase TBK1 OS=Homo sapiens GN=TBK1 PE=1 SV=1 | 295 | | 84216 |
| sp|P07948|LYN_HUMAN | Tyrosine-protein kinase Lyn OS=Homo sapiens GN=LYN PE=1 SV=3 | 2553 | | 58993 |
| sp|P08631|HCK_HUMAN | Tyrosine-protein kinase HCK OS=Homo sapiens GN=HCK PE=1 SV=5 | 343 | | 60075 |
| sp|Q06187|BTK_HUMAN | Tyrosine-protein kinase BTK OS=Homo sapiens GN=BTK PE=1 SV=3 | 706 | | 76917 |
| sp|P05771|KPCB_HUMAN | Protein kinase C beta type OS=Homo sapiens GN=PRKCB PE=1 SV=4 | 553 | | 77960 |
| sp|P17252|KPCA_HUMAN | Protein kinase C alpha type OS=Homo sapiens GN=PRKCA PE=1 SV=4 | 293 | | 77841 |
| sp|Q05655|KPCD_HUMAN | Protein kinase C delta type OS=Homo sapiens GN=PRKCD PE=1 SV=2 | 413 | | 78652 |
| sp|P30085|KCY_HUMAN | UMP-CMP kinase OS=Homo sapiens GN=CMPK1 PE=1 SV=3 | 760 | | 22436 |
| sp|P23919|KTHY_HUMAN | Thymidylate kinase OS=Homo sapiens GN=DTYMK PE=1 SV=4 | 772 | | 23976 |
| sp|Q15126|PMVK_HUMAN | Phosphomevalonate kinase OS=Homo sapiens GN=PMVK PE=1 SV=3 | 358 | | 22152 |
| sp|P15531|NDKA_HUMAN | Nucleoside diphosphate kinase A OS=Homo sapiens GN=NME1 PE=1 SV=1 | 956 | | 17309 |
| sp|P00558|PGK1_HUMAN | Phosphoglycerate kinase 1 OS=Homo sapiens GN=PGK1 PE=1 SV=3 | 3517 | | 44985 |
| sp|P43250|GRK6_HUMAN | G protein-coupled receptor kinase 6 OS=Homo sapiens GN=GRK6 PE=1 SV=2 | 307 | | 67203 |
| sp|Q02750|MP2K1_HUMAN | Dual specificity mitogen-activated protein kinase kinase 1 OS=Homo sapiens GN=MAP2K1 PE=1 SV=2 | 199 | | 43753 |
| sp|Q01813|K6PP_HUMAN | 6-phosphofructokinase type C OS=Homo sapiens GN=PFKP PE=1 SV=2 | 2812 | | 86454 |
| sp|P42356|PI4KA_HUMAN | Phosphatidylinositol 4-kinase alpha OS=Homo sapiens GN=PI4KA PE=1 SV=3 | 452 | | 233622 |
| sp|Q53R41|FAKD1_HUMAN | FAST kinase domain-containing protein 1 OS=Homo sapiens GN=FASTKD1 PE=1 SV=1 | 499 | | 98374 |
| sp|P28482|MK01_HUMAN | Mitogen-activated protein kinase 1 OS=Homo sapiens GN=MAPK1 PE=1 SV=3 | 355 | | 41762 |
| sp|P25098|ARBK1_HUMAN | Beta-adrenergic receptor kinase 1 OS=Homo sapiens GN=ADRBK1 PE=1 SV=2 | 1039 | | 80321 |
| sp|P13861|KAP2_HUMAN | cAMP-dependent protein kinase type II-alpha regulatory subunit OS=Homo sapiens GN=PRKAR2A PE=1 SV=2 | 417 | | 45832 |
| sp|P54819|KAD2_HUMAN | Adenylate kinase 2, mitochondrial OS=Homo sapiens GN=AK2 PE=1 SV=2 | 873 | | 26689 |
| NADH dehydrogenase | | | |  |
| sp|O43678|NDUA2_HUMAN | NADH dehydrogenase [ubiquinone] 1 alpha subcomplex subunit 2 OS=Homo sapiens GN=NDUFA2 PE=1 SV=3 | | 538 | 11029 |
| sp|Q9P032|NDUF4_HUMAN | NADH dehydrogenase [ubiquinone] 1 alpha subcomplex assembly factor 4 OS=Homo sapiens GN=NDUFAF4 PE=1 SV=1 | | 753 | 20311 |
| sp|O96000|NDUBA_HUMAN | NADH dehydrogenase [ubiquinone] 1 beta subcomplex subunit 10 OS=Homo sapiens GN=NDUFB10 PE=1 SV=3 | | 558 | 20548 |
| sp|P51970|NDUA8_HUMAN | NADH dehydrogenase [ubiquinone] 1 alpha subcomplex subunit 8 OS=Homo sapiens GN=NDUFA8 PE=1 SV=3 | | 753 | 21048 |
| sp|O95169|NDUB8_HUMAN | NADH dehydrogenase [ubiquinone] 1 beta subcomplex subunit 8, mitochondrial OS=Homo sapiens GN=NDUFB8 PE=1 SV=1 | | 413 | 21865 |
| sp|Q9Y6M9|NDUB9_HUMAN | NADH dehydrogenase [ubiquinone] 1 beta subcomplex subunit 9 OS=Homo sapiens GN=NDUFB9 PE=1 SV=3 | | 1171 | 22045 |
| sp|P49821|NDUV1_HUMAN | NADH dehydrogenase [ubiquinone] flavoprotein 1, mitochondrial OS=Homo sapiens GN=NDUFV1 PE=1 SV=4 | | 984 | 41067 |
| sp|O95299|NDUAA_HUMAN | NADH dehydrogenase [ubiquinone] 1 alpha subcomplex subunit 10, mitochondrial OS=Homo sapiens GN=NDUFA10 PE=1 SV=1 | | 852 | 51469 |
| sp|P40926|MDHM_HUMAN | Malate dehydrogenase, mitochondrial OS=Homo sapiens GN=MDH2 PE=1 SV=3 | | 6040 | 35937 |
| sp|P08559|ODPA_HUMAN | Pyruvate dehydrogenase E1 component subunit alpha, somatic form, mitochondrial OS=Homo sapiens GN=PDHA1 PE=1 SV=3 | | 391 | 43952 |
| sp|P11177|ODPB_HUMAN | Pyruvate dehydrogenase E1 component subunit beta, mitochondrial OS=Homo sapiens GN=PDHB PE=1 SV=3 | | 413 | 39550 |
| sp|P09622|DLDH_HUMAN | Dihydrolipoyl dehydrogenase, mitochondrial OS=Homo sapiens GN=DLD PE=1 SV=2 | | 3626 | 54713 |
| sp|Q3SY69|AL1L2_HUMAN | Mitochondrial 10-formyltetrahydrofolate dehydrogenase OS=Homo sapiens GN=ALDH1L2 PE=1 SV=2 | | 708 | 102365 |
| sp|Q8NBQ5|DHB11_HUMAN | Estradiol 17-beta-dehydrogenase 11 OS=Homo sapiens GN=HSD17B11 PE=1 SV=3 | | 340 | 33257 |
| sp|P30038|AL4A1_HUMAN | Delta-1-pyrroline-5-carboxylate dehydrogenase, mitochondrial OS=Homo sapiens GN=ALDH4A1 PE=1 SV=3 | | 498 | 62137 |
| sp|P12268|IMDH2_HUMAN | Inosine-5~-monophosphate dehydrogenase 2 OS=Homo sapiens GN=IMPDH2 PE=1 SV=2 | | 3154 | 56226 |
| sp|Q6YN16|HSDL2_HUMAN | Hydroxysteroid dehydrogenase-like protein 2 OS=Homo sapiens GN=HSDL2 PE=1 SV=1 | | 631 | 45651 |
| sp|Q02127|PYRD_HUMAN | Dihydroorotate dehydrogenase (quinone), mitochondrial OS=Homo sapiens GN=DHODH PE=1 SV=3 | | 442 | 42841 |
| sp|P00338|LDHA_HUMAN | L-lactate dehydrogenase A chain OS=Homo sapiens GN=LDHA PE=1 SV=2 | | 2406 | 36950 |
| sp|P07195|LDHB_HUMAN | L-lactate dehydrogenase B chain OS=Homo sapiens GN=LDHB PE=1 SV=2 | | 2358 | 36900 |
| sp|P50213|IDH3A_HUMAN | Isocitrate dehydrogenase [NAD] subunit alpha, mitochondrial OS=Homo sapiens GN=IDH3A PE=1 SV=1 | | 369 | 40022 |
| sp|Q02218|ODO1_HUMAN | 2-oxoglutarate dehydrogenase, mitochondrial OS=Homo sapiens GN=OGDH PE=1 SV=3 | | 2322 | 117059 |
| sp|Q96HY7|DHTK1_HUMAN | Probable 2-oxoglutarate dehydrogenase E1 component DHKTD1, mitochondrial OS=Homo sapiens GN=DHTKD1 PE=1 SV=2 | | 206 | 103752 |
| sp|P36957|ODO2_HUMAN | Dihydrolipoyllysine-residue succinyltransferase component of 2-oxoglutarate dehydrogenase complex, mitochondrial OS=Homo sapiens GN=DLST PE=1 SV=4 | | 2141 | 49067 |
| sp|P10515|ODP2_HUMAN | Dihydrolipoyllysine-residue acetyltransferase component of pyruvate dehydrogenase complex, mitochondrial OS=Homo sapiens GN=DLAT PE=1 SV=3 | | 220 | 69466 |
| sp|P11413|G6PD_HUMAN | Glucose-6-phosphate 1-dehydrogenase OS=Homo sapiens GN=G6PD PE=1 SV=4 | | 1315 | 59675 |
| sp|Q9BTZ2|DHRS4_HUMAN | Dehydrogenase/reductase SDR family member 4 OS=Homo sapiens GN=DHRS4 PE=1 SV=3 | | 421 | 29803 |
| sp|P14550|AK1A1_HUMAN | Alcohol dehydrogenase [NADP(+)] OS=Homo sapiens GN=AKR1A1 PE=1 SV=3 | | 250 | 36892 |
| sp|P05091|ALDH2_HUMAN | Aldehyde dehydrogenase, mitochondrial OS=Homo sapiens GN=ALDH2 PE=1 SV=2 | | 1576 | 56859 |
| sp|Q8IZ83|A16A1_HUMAN | Aldehyde dehydrogenase family 16 member A1 OS=Homo sapiens GN=ALDH16A1 PE=1 SV=2 | | 587 | 86100 |
| sp|O43175|SERA_HUMAN | D-3-phosphoglycerate dehydrogenase OS=Homo sapiens GN=PHGDH PE=1 SV=4 | | 1354 | 57356 |
| sp|P04406|G3P_HUMAN | Glyceraldehyde-3-phosphate dehydrogenase OS=Homo sapiens GN=GAPDH PE=1 SV=3 | | 4191 | 36201 |
| sp|P31040|DHSA_HUMAN | Succinate dehydrogenase [ubiquinone] flavoprotein subunit, mitochondrial OS=Homo sapiens GN=SDHA PE=1 SV=2 | | 261 | 73672 |
| phosphatase | | |  |  |
| sp|P62136|PP1A_HUMAN | Serine/threonine-protein phosphatase PP1-alpha catalytic subunit OS=Homo sapiens GN=PPP1CA PE=1 SV=1 | | 1197 | 38229 |
| sp|P36873|PP1G_HUMAN | Serine/threonine-protein phosphatase PP1-gamma catalytic subunit OS=Homo sapiens GN=PPP1CC PE=1 SV=1 | | 1036 | 37701 |
| sp|Q96HS1|PGAM5_HUMAN | Serine/threonine-protein phosphatase PGAM5, mitochondrial OS=Homo sapiens GN=PGAM5 PE=1 SV=2 | | 923 | 32213 |
| sp|P62140|PP1B_HUMAN | Serine/threonine-protein phosphatase PP1-beta catalytic subunit OS=Homo sapiens GN=PPP1CB PE=1 SV=3 | | 703 | 37961 |
| sp|Q92835|SHIP1_HUMAN | Phosphatidylinositol 3,4,5-trisphosphate 5-phosphatase 1 OS=Homo sapiens GN=INPP5D PE=1 SV=2 | | 242 | 134121 |
| sp|P10586|PTPRF_HUMAN | Receptor-type tyrosine-protein phosphatase F OS=Homo sapiens GN=PTPRF PE=1 SV=2 | | 723 | 213942 |
| sp|P08575|PTPRC_HUMAN | Receptor-type tyrosine-protein phosphatase C OS=Homo sapiens GN=PTPRC PE=1 SV=2 | | 487 | 148644 |
| sp|Q15181|IPYR_HUMAN | Inorganic pyrophosphatase OS=Homo sapiens GN=PPA1 PE=1 SV=2 | | 649 | 33095 |
| sp|Q8WUK0|PTPM1_HUMAN | Phosphatidylglycerophosphatase and protein-tyrosine phosphatase 1 OS=Homo sapiens GN=PTPMT1 PE=1 SV=1 | | 525 | 23000 |
| isomerase |  | |  |  |
| Protein disulfide-isomerase | | | |  |
| sp|P30101|PDIA3_HUMAN | Protein disulfide-isomerase A3 OS=Homo sapiens GN=PDIA3 PE=1 SV=4 | | 7672 | 57146 |
| sp|Q96JJ7|TMX3_HUMAN | Protein disulfide-isomerase TMX3 OS=Homo sapiens GN=TMX3 PE=1 SV=2 | | 798 | 52181 |
| sp|P07237|PDIA1_HUMAN | Protein disulfide-isomerase OS=Homo sapiens GN=P4HB PE=1 SV=3 | | 4993 | 57480 |
| sp|Q15084|PDIA6_HUMAN | Protein disulfide-isomerase A6 OS=Homo sapiens GN=PDIA6 PE=1 SV=1 | | 3028 | 48490 |
| sp|P13667|PDIA4_HUMAN | Protein disulfide-isomerase A4 OS=Homo sapiens GN=PDIA4 PE=1 SV=2 | | 5186 | 73229 |
| sp|P11387|TOP1_HUMAN | DNA topoisomerase 1 OS=Homo sapiens GN=TOP1 PE=1 SV=2 | | 448 | 91125 |
| sp|P11388|TOP2A_HUMAN | DNA topoisomerase 2-alpha OS=Homo sapiens GN=TOP2A PE=1 SV=3 | | 238 | 175017 |
| sp|O43447|PPIH_HUMAN | Peptidyl-prolyl cis-trans isomerase H OS=Homo sapiens GN=PPIH PE=1 SV=1 | | 700 | 19481 |
| sp|Q02790|FKBP4_HUMAN | Peptidyl-prolyl cis-trans isomerase FKBP4 OS=Homo sapiens GN=FKBP4 PE=1 SV=3 | | 459 | 52057 |
| Aminopeptidase | | |  |  |
| sp|P15144|AMPN_HUMAN | Aminopeptidase N OS=Homo sapiens GN=ANPEP PE=1 SV=4 | | 180 | 109870 |
| sp|P55786|PSA_HUMAN | Puromycin-sensitive aminopeptidase OS=Homo sapiens GN=NPEPPS PE=1 SV=2 | | 1197 | 103895 |
| sp|Q9NZ08|ERAP1_HUMAN | Endoplasmic reticulum aminopeptidase 1 OS=Homo sapiens GN=ERAP1 PE=1 SV=3 | | 1134 | 107736 |
| sp|P28838|AMPL_HUMAN | Cytosol aminopeptidase OS=Homo sapiens GN=LAP3 PE=1 SV=3 | | 363 | 56530 |
| sp|Q9NQH7|XPP3_HUMAN | Probable Xaa-Pro aminopeptidase 3 OS=Homo sapiens GN=XPNPEP3 PE=1 SV=1 | | 269 | 57624 |
| phosphorylase | | |  |  |
| sp|P06737|PYGL_HUMAN | Glycogen phosphorylase, liver form OS=Homo sapiens GN=PYGL PE=1 SV=4 | | 598 | 97486 |
| sp|P11216|PYGB_HUMAN | Glycogen phosphorylase, brain form OS=Homo sapiens GN=PYGB PE=1 SV=5 | | 484 | 97319 |
| sp|P19971|TYPH_HUMAN | Thymidine phosphorylase OS=Homo sapiens GN=TYMP PE=1 SV=2 | | 1382 | 50323 |
| sp|P00491|PNPH_HUMAN | Purine nucleoside phosphorylase OS=Homo sapiens GN=PNP PE=1 SV=2 | | 384 | 32325 |
| aldolase |  | |  |  |
| sp|P04075|ALDOA_HUMAN | Fructose-bisphosphate aldolase A OS=Homo sapiens GN=ALDOA PE=1 SV=2 | | 3799 | 39851 |
| sp|P09972|ALDOC_HUMAN | Fructose-bisphosphate aldolase C OS=Homo sapiens GN=ALDOC PE=1 SV=2 | | 1140 | 39830 |
| sp|P37837|TALDO_HUMAN | Transaldolase OS=Homo sapiens GN=TALDO1 PE=1 SV=2 | | 1239 | 37688 |
| synthase |  | |  |  |
| sp|P11586|C1TC_HUMAN | C-1-tetrahydrofolate synthase, cytoplasmic OS=Homo sapiens GN=MTHFD1 PE=1 SV=3 | | 2370 | 102180 |
| sp|P53396|ACLY_HUMAN | ATP-citrate synthase OS=Homo sapiens GN=ACLY PE=1 SV=3 | | 1424 | 121674 |
| sp|O00116|ADAS_HUMAN | Alkyldihydroxyacetonephosphate synthase, peroxisomal OS=Homo sapiens GN=AGPS PE=1 SV=1 | | 309 | 73664 |
| sp|P19623|SPEE_HUMAN | Spermidine synthase OS=Homo sapiens GN=SRM PE=1 SV=1 | | 300 | 34373 |
| sp|P54886|P5CS_HUMAN | Delta-1-pyrroline-5-carboxylate synthase OS=Homo sapiens GN=ALDH18A1 PE=1 SV=2 | | 453 | 87989 |
| sp|Q6UB35|C1TM_HUMAN | Monofunctional C1-tetrahydrofolate synthase, mitochondrial OS=Homo sapiens GN=MTHFD1L PE=1 SV=1 | | 2439 | 106636 |
| sp|Q13057|COASY_HUMAN | Bifunctional coenzyme A synthase OS=Homo sapiens GN=COASY PE=1 SV=4 | | 326 | 62632 |
| sp|P17812|PYRG1_HUMAN | CTP synthase 1 OS=Homo sapiens GN=CTPS1 PE=1 SV=2 | | 733 | 67332 |
| others |  | |  |  |
| sp|Q13423|NNTM_HUMAN | NAD(P) transhydrogenase, mitochondrial OS=Homo sapiens GN=NNT PE=1 SV=3 | | 304 | 114564 |
| sp|Q7KZF4|SND1_HUMAN | Staphylococcal nuclease domain-containing protein 1 OS=Homo sapiens GN=SND1 PE=1 SV=1 | | 945 | 102618 |
| sp|P04040|CATA_HUMAN | Catalase OS=Homo sapiens GN=CAT PE=1 SV=3 | | 3944 | 59947 |
| sp|P07203|GPX1_HUMAN | Glutathione peroxidase 1 OS=Homo sapiens GN=GPX1 PE=1 SV=4 | | 1084 | 22360 |
| sp|Q460N5|PAR14_HUMAN | Poly [ADP-ribose] polymerase 14 OS=Homo sapiens GN=PARP14 PE=1 SV=3 | | 93 | 204725 |
| sp|P09874|PARP1_HUMAN | Poly [ADP-ribose] polymerase 1 OS=Homo sapiens GN=PARP1 PE=1 SV=4 | | 730 | 113811 |
| sp|O60568|PLOD3_HUMAN | Procollagen-lysine,2-oxoglutarate 5-dioxygenase 3 OS=Homo sapiens GN=PLOD3 PE=1 SV=1 | | 415 | 85302 |
| sp|Q02809|PLOD1_HUMAN | Procollagen-lysine,2-oxoglutarate 5-dioxygenase 1 OS=Homo sapiens GN=PLOD1 PE=1 SV=2 | | 3442 | 84068 |
| sp|Q9C0B5|ZDHC5_HUMAN | Palmitoyltransferase ZDHHC5 OS=Homo sapiens GN=ZDHHC5 PE=1 SV=2 | | 528 | 78237 |
| sp|O15269|SPTC1_HUMAN | Serine palmitoyltransferase 1 OS=Homo sapiens GN=SPTLC1 PE=1 SV=1 | | 1452 | 53281 |
| sp|P50416|CPT1A_HUMAN | Carnitine O-palmitoyltransferase 1, liver isoform OS=Homo sapiens GN=CPT1A PE=1 SV=2 | | 2726 | 88995 |
| sp|P36776|LONM_HUMAN | Lon protease homolog, mitochondrial OS=Homo sapiens GN=LONP1 PE=1 SV=2 | | 5974 | 106936 |
| sp|Q14697|GANAB_HUMAN | Neutral alpha-glucosidase AB OS=Homo sapiens GN=GANAB PE=1 SV=3 | | 358 | 107263 |
| sp|Q13724|MOGS_HUMAN | Mannosyl-oligosaccharide glucosidase OS=Homo sapiens GN=MOGS PE=1 SV=5 | | 3044 | 92032 |
| sp|P13674|P4HA1_HUMAN | Prolyl 4-hydroxylase subunit alpha-1 OS=Homo sapiens GN=P4HA1 PE=1 SV=2 | | 1993 | 61296 |
| sp|Q32P28|P3H1_HUMAN | Prolyl 3-hydroxylase 1 OS=Homo sapiens GN=LEPRE1 PE=1 SV=2 | | 799 | 84196 |
| sp|Q99798|ACON_HUMAN | Aconitate hydratase, mitochondrial OS=Homo sapiens GN=ACO2 PE=1 SV=2 | | 5337 | 86113 |
| sp|Q3ZCQ8|TIM50_HUMAN | Mitochondrial import inner membrane translocase subunit TIM50 OS=Homo sapiens GN=TIMM50 PE=1 SV=2 | | 901 | 39850 |
| sp|P24752|THIL_HUMAN | Acetyl-CoA acetyltransferase, mitochondrial OS=Homo sapiens GN=ACAT1 PE=1 SV=1 | | 263 | 45456 |
| sp|Q9BWD1|THIC_HUMAN | Acetyl-CoA acetyltransferase, cytosolic OS=Homo sapiens GN=ACAT2 PE=1 SV=2 | | 571 | 41838 |
| sp|Q9H0A0|NAT10_HUMAN | N-acetyltransferase 10 OS=Homo sapiens GN=NAT10 PE=1 SV=2 | | 426 | 116569 |
| sp|O60341|KDM1A_HUMAN | Lysine-specific histone demethylase 1A OS=Homo sapiens GN=KDM1A PE=1 SV=2 | | 308 | 93358 |
| sp|P23921|RIR1_HUMAN | Ribonucleoside-diphosphate reductase large subunit OS=Homo sapiens GN=RRM1 PE=1 SV=1 | | 660 | 90925 |
| sp|P31350|RIR2_HUMAN | Ribonucleoside-diphosphate reductase subunit M2 OS=Homo sapiens GN=RRM2 PE=1 SV=1 | | 219 | 45134 |
| sp|P16435|NCPR_HUMAN | NADPH--cytochrome P450 reductase OS=Homo sapiens GN=POR PE=1 SV=2 | | 935 | 77097 |
| sp|P00387|NB5R3_HUMAN | NADH-cytochrome b5 reductase 3 OS=Homo sapiens GN=CYB5R3 PE=1 SV=3 | | 1143 | 34441 |
| sp|Q9UBQ7|GRHPR_HUMAN | Glyoxylate reductase/hydroxypyruvate reductase OS=Homo sapiens GN=GRHPR PE=1 SV=1 | | 345 | 36045 |
| sp|Q96C36|P5CR2_HUMAN | Pyrroline-5-carboxylate reductase 2 OS=Homo sapiens GN=PYCR2 PE=1 SV=1 | | 2138 | 33958 |
| sp|P28331|NDUS1_HUMAN | NADH-ubiquinone oxidoreductase 75 kDa subunit, mitochondrial OS=Homo sapiens GN=NDUFS1 PE=1 SV=3 | | 818 | 80443 |
| sp|Q01518|CAP1_HUMAN | Adenylyl cyclase-associated protein 1 OS=Homo sapiens GN=CAP1 PE=1 SV=5 | | 10709 | 52325 |
| sp|Q9Y2P8|RCL1_HUMAN | RNA 3~-terminal phosphate cyclase-like protein OS=Homo sapiens GN=RCL1 PE=1 SV=3 | | 536 | 41273 |
| sp|P55809|SCOT1_HUMAN | Succinyl-CoA:3-ketoacid coenzyme A transferase 1, mitochondrial OS=Homo sapiens GN=OXCT1 PE=1 SV=1 | | 1880 | 56578 |
| sp|P23526|SAHH_HUMAN | Adenosylhomocysteinase OS=Homo sapiens GN=AHCY PE=1 SV=4 | | 328 | 48255 |
| sp|Q16555|DPYL2_HUMAN | Dihydropyrimidinase-related protein 2 OS=Homo sapiens GN=DPYSL2 PE=1 SV=1 | | 935 | 62711 |
| sp|Q9H488|OFUT1_HUMAN | GDP-fucose protein O-fucosyltransferase 1 OS=Homo sapiens GN=POFUT1 PE=1 SV=1 | | 211 | 44383 |
| sp|Q8NF37|PCAT1_HUMAN | Lysophosphatidylcholine acyltransferase 1 OS=Homo sapiens GN=LPCAT1 PE=1 SV=2 | | 203 | 59741 |
| sp|O95870|ABHGA_HUMAN | Abhydrolase domain-containing protein 16A OS=Homo sapiens GN=ABHD16A PE=1 SV=3 | | 311 | 63830 |
| sp|Q8TCS8|PNPT1_HUMAN | Polyribonucleotide nucleotidyltransferase 1, mitochondrial OS=Homo sapiens GN=PNPT1 PE=1 SV=2 | | 4511 | 86524 |
| sp|P51659|DHB4_HUMAN | Peroxisomal multifunctional enzyme type 2 OS=Homo sapiens GN=HSD17B4 PE=1 SV=3 | | 3618 | 80092 |
| sp|P22087|FBRL_HUMAN | rRNA 2~-O-methyltransferase fibrillarin OS=Homo sapiens GN=FBL PE=1 SV=2 | | 2281 | 33877 |
| sp|O60488|ACSL4_HUMAN | Long-chain-fatty-acid--CoA ligase 4 OS=Homo sapiens GN=ACSL4 PE=1 SV=2 | | 1607 | 80220 |
| sp|P50897|PPT1_HUMAN | Palmitoyl-protein thioesterase 1 OS=Homo sapiens GN=PPT1 PE=1 SV=1 | | 982 | 34627 |
| sp|P22033|MUTA_HUMAN | Methylmalonyl-CoA mutase, mitochondrial OS=Homo sapiens GN=MUT PE=1 SV=4 | | 715 | 83538 |
| sp|Q06210|GFPT1_HUMAN | Glutamine--fructose-6-phosphate aminotransferase [isomerizing] 1 OS=Homo sapiens GN=GFPT1 PE=1 SV=3 | | 709 | 79555 |
| sp|Q8NBJ5|GT251_HUMAN | Procollagen galactosyltransferase 1 OS=Homo sapiens GN=COLGALT1 PE=1 SV=1 | | 1545 | 71933 |
| sp|P33121|ACSL1_HUMAN | Long-chain-fatty-acid--CoA ligase 1 OS=Homo sapiens GN=ACSL1 PE=1 SV=1 | | 1319 | 78919 |
| sp|Q9NXE4|NSMA3_HUMAN | Sphingomyelin phosphodiesterase 4 OS=Homo sapiens GN=SMPD4 PE=1 SV=2 | | 1639 | 94033 |
| sp|P34897|GLYM_HUMAN | Serine hydroxymethyltransferase, mitochondrial OS=Homo sapiens GN=SHMT2 PE=1 SV=3 | | 365 | 56414 |
| sp|P26572|MGAT1_HUMAN | Alpha-1,3-mannosyl-glycoprotein 2-beta-N-acetylglucosaminyltransferase OS=Homo sapiens GN=MGAT1 PE=2 SV=2 | | 436 | 51132 |
| sp|P14735|IDE_HUMAN | Insulin-degrading enzyme OS=Homo sapiens GN=IDE PE=1 SV=4 | | 515 | 118692 |
| sp|Q9UHG3|PCYOX_HUMAN | Prenylcysteine oxidase 1 OS=Homo sapiens GN=PCYOX1 PE=1 SV=3 | | 1232 | 57003 |
| sp|Q9Y3I0|RTCB_HUMAN | tRNA-splicing ligase RtcB homolog OS=Homo sapiens GN=RTCB PE=1 SV=1 | | 399 | 55688 |
| sp|Q6PIU2|NCEH1_HUMAN | Neutral cholesterol ester hydrolase 1 OS=Homo sapiens GN=NCEH1 PE=1 SV=3 | | 529 | 46064 |
| sp|Q86TX2|ACOT1_HUMAN | Acyl-coenzyme A thioesterase 1 OS=Homo sapiens GN=ACOT1 PE=1 SV=1 | | 398 | 46647 |
| sp|Q9BQ52|RNZ2_HUMAN | Zinc phosphodiesterase ELAC protein 2 OS=Homo sapiens GN=ELAC2 PE=1 SV=2 | | 422 | 93415 |
| sp|Q96I99|SUCB2_HUMAN | Succinyl-CoA ligase [GDP-forming] subunit beta, mitochondrial OS=Homo sapiens GN=SUCLG2 PE=1 SV=2 | | 1112 | 46824 |
| sp|P42765|THIM_HUMAN | 3-ketoacyl-CoA thiolase, mitochondrial OS=Homo sapiens GN=ACAA2 PE=1 SV=2 | | 930 | 42354 |
| sp|Q08J23|NSUN2_HUMAN | tRNA (cytosine(34)-C(5))-methyltransferase OS=Homo sapiens GN=NSUN2 PE=1 SV=2 | | 287 | 87214 |
| sp|Q7Z2K6|ERMP1_HUMAN | Endoplasmic reticulum metallopeptidase 1 OS=Homo sapiens GN=ERMP1 PE=1 SV=2 | | 344 | 101023 |
| sp|Q96S52|PIGS_HUMAN | GPI transamidase component PIG-S OS=Homo sapiens GN=PIGS PE=1 SV=3 | | 609 | 61731 |
| sp|O75976|CBPD_HUMAN | Carboxypeptidase D OS=Homo sapiens GN=CPD PE=1 SV=2 | | 168 | 153919 |
| sp|P22061|PIMT_HUMAN | Protein-L-isoaspartate(D-aspartate) O-methyltransferase OS=Homo sapiens GN=PCMT1 PE=1 SV=4 | | 516 | 24792 |
| sp|P04179|SODM_HUMAN | Superoxide dismutase [Mn], mitochondrial OS=Homo sapiens GN=SOD2 PE=1 SV=2 | | 554 | 24878 |
| sp|P29144|TPP2_HUMAN | Tripeptidyl-peptidase 2 OS=Homo sapiens GN=TPP2 PE=1 SV=4 | | 328 | 139745 |
| sp|Q8TDZ2|MICA1_HUMAN | Protein-methionine sulfoxide oxidase MICAL1 OS=Homo sapiens GN=MICAL1 PE=1 SV=2 | | 299 | 118884 |
| sp|Q5JRX3|PREP_HUMAN | Presequence protease, mitochondrial OS=Homo sapiens GN=PITRM1 PE=1 SV=3 | | 205 | 118364 |
| sp|P07741|APT_HUMAN | Adenine phosphoribosyltransferase OS=Homo sapiens GN=APRT PE=1 SV=2 | | 1250 | 19766 |
| sp|Q9Y3E5|PTH2_HUMAN | Peptidyl-tRNA hydrolase 2, mitochondrial OS=Homo sapiens GN=PTRH2 PE=1 SV=1 | | 420 | 19466 |
| sp|Q04760|LGUL_HUMAN | Lactoylglutathione lyase OS=Homo sapiens GN=GLO1 PE=1 SV=4 | | 327 | 20992 |
| sp|Q9BZQ6|EDEM3_HUMAN | ER degradation-enhancing alpha-mannosidase-like protein 3 OS=Homo sapiens GN=EDEM3 PE=1 SV=2 | | 122 | 105225 |
| sp|P23141|EST1_HUMAN | Liver carboxylesterase 1 OS=Homo sapiens GN=CES1 PE=1 SV=2 | | 1940 | 62766 |
| sp|P06733|ENOA_HUMAN | Alpha-enolase OS=Homo sapiens GN=ENO1 PE=1 SV=2 | | 5331 | 47481 |
| sp|P04843|RPN1_HUMAN | Dolichyl-diphosphooligosaccharide--protein glycosyltransferase subunit 1 OS=Homo sapiens GN=RPN1 PE=1 SV=1 | | 6463 | 68641 |
| sp|P04181|OAT_HUMAN | Ornithine aminotransferase, mitochondrial OS=Homo sapiens GN=OAT PE=1 SV=1 | | 1238 | 48846 |
| sp|P36871|PGM1_HUMAN | Phosphoglucomutase-1 OS=Homo sapiens GN=PGM1 PE=1 SV=3 | | 264 | 61696 |
| sp|P40939|ECHA_HUMAN | Trifunctional enzyme subunit alpha, mitochondrial OS=Homo sapiens GN=HADHA PE=1 SV=2 | | 496 | 83688 |
| sp|Q10471|GALT2_HUMAN | Polypeptide N-acetylgalactosaminyltransferase 2 OS=Homo sapiens GN=GALNT2 PE=1 SV=1 | | 454 | 65433 |
| sp|Q9HCC0|MCCB_HUMAN | Methylcrotonoyl-CoA carboxylase beta chain, mitochondrial OS=Homo sapiens GN=MCCC2 PE=1 SV=1 | | 738 | 61808 |
| sp|P29401|TKT_HUMAN | Transketolase OS=Homo sapiens GN=TKT PE=1 SV=3 | | 387 | 68519 |
| sp|P13798|ACPH_HUMAN | Acylamino-acid-releasing enzyme OS=Homo sapiens GN=APEH PE=1 SV=4 | | 490 | 82142 |
| sp|O95865|DDAH2_HUMAN | N(G),N(G)-dimethylarginine dimethylaminohydrolase 2 OS=Homo sapiens GN=DDAH2 PE=1 SV=1 | | 728 | 29911 |
| sp|P08236|BGLR_HUMAN | Beta-glucuronidase OS=Homo sapiens GN=GUSB PE=1 SV=2 | | 301 | 75027 |
| sp|P78417|GSTO1_HUMAN | Glutathione S-transferase omega-1 OS=Homo sapiens GN=GSTO1 PE=1 SV=2 | | 311 | 27833 |
| sp|Q9UIJ7|KAD3_HUMAN | GTP:AMP phosphotransferase AK3, mitochondrial OS=Homo sapiens GN=AK3 PE=1 SV=4 | | 532 | 25550 |
| sp|Q6L8Q7|PDE12_HUMAN | 2~,5~-phosphodiesterase 12 OS=Homo sapiens GN=PDE12 PE=1 SV=2 | | 279 | 68221 |
| sp|P62495|ERF1_HUMAN | Eukaryotic peptide chain release factor subunit 1 OS=Homo sapiens GN=ETF1 PE=1 SV=3 | | 349 | 49228 |
| sp|P00918|CAH2_HUMAN | Carbonic anhydrase 2 OS=Homo sapiens GN=CA2 PE=1 SV=2 | | 515 | 29285 |

| **Accession number** | **Protein name** | **Score** | **MW** |
| --- | --- | --- | --- |
| **(19) others** | |  |  |
| HLA class I histocompatibility antigens | | | |
| sp|P01892|1A02_HUMAN | HLA class I histocompatibility antigen, A-2 alpha chain OS=Homo sapiens GN=HLA-A PE=1 SV=1 | 1988 | 40541 |
| sp|Q04826|1B40_HUMAN | HLA class I histocompatibility antigen, B-40 alpha chain OS=Homo sapiens GN=HLA-B PE=1 SV=1 | 1385 | 40640 |
| sp|P30508|1C12_HUMAN | HLA class I histocompatibility antigen, Cw-12 alpha chain OS=Homo sapiens GN=HLA-C PE=2 SV=2 | 1086 | 40648 |
| sp|P10316|1A69_HUMAN | HLA class I histocompatibility antigen, A-69 alpha chain OS=Homo sapiens GN=HLA-A PE=1 SV=2 | 2659 | 40716 |
| sp|P30508|1C12_HUMAN | HLA class I histocompatibility antigen, Cw-12 alpha chain OS=Homo sapiens GN=HLA-C PE=2 SV=2 | 2172 | 40756 |
| sp|P18465|1B57_HUMAN | HLA class I histocompatibility antigen, B-57 alpha chain OS=Homo sapiens GN=HLA-B PE=1 SV=1 | 2146 | 40822 |
| sp|P30453|1A34_HUMAN | HLA class I histocompatibility antigen, A-34 alpha chain OS=Homo sapiens GN=HLA-A PE=1 SV=1 | 2144 | 40856 |
| sp|P30493|1B55_HUMAN | HLA class I histocompatibility antigen, B-55 alpha chain OS=Homo sapiens GN=HLA-B PE=1 SV=1 | 1995 | 41181 |
| sp|P01892|1A02_HUMAN | HLA class I histocompatibility antigen, A-2 alpha chain OS=Homo sapiens GN=HLA-A PE=1 SV=1 | 1423 | 41234 |
| sp|P18465|1B57_HUMAN | HLA class I histocompatibility antigen, B-57 alpha chain OS=Homo sapiens GN=HLA-B PE=1 SV=1 | 1230 | 41236 |
| sp|P30492|1B54_HUMAN | HLA class I histocompatibility antigen, B-54 alpha chain OS=Homo sapiens GN=HLA-B PE=1 SV=1 | 1057 | 41293 |
| sp|P18465|1B57_HUMAN | HLA class I histocompatibility antigen, B-57 alpha chain OS=Homo sapiens GN=HLA-B PE=1 SV=1 | 1230 | 41314 |
| sp|P30464|1B15_HUMAN | HLA class I histocompatibility antigen, B-15 alpha chain OS=Homo sapiens GN=HLA-B PE=1 SV=2 | 1213 | 41316 |
| Apoptosis-related proteins | | |  |
| sp|P50995|ANX11_HUMAN | Annexin A11 OS=Homo sapiens GN=ANXA11 PE=1 SV=1 | 913 | 54697 |
| sp|P04083|ANXA1_HUMAN | Annexin A1 OS=Homo sapiens GN=ANXA1 PE=1 SV=2 | 4217 | 38918 |
| sp|P07355|ANXA2_HUMAN | Annexin A2 OS=Homo sapiens GN=ANXA2 PE=1 SV=2 | 5036 | 38808 |
| sp|P08133|ANXA6_HUMAN | Annexin A6 OS=Homo sapiens GN=ANXA6 PE=1 SV=3 | 223 | 76168 |
| sp|O95831|AIFM1_HUMAN | Apoptosis-inducing factor 1, mitochondrial OS=Homo sapiens GN=AIFM1 PE=1 SV=1 | 1360 | 67144 |
| sp|Q9ULZ3|ASC_HUMAN | Apoptosis-associated speck-like protein containing a CARD OS=Homo sapiens GN=PYCARD PE=1 SV=2 | 545 | 21670 |
| sp|Q8WUM4|PDC6I_HUMAN | Programmed cell death 6-interacting protein OS=Homo sapiens GN=PDCD6IP PE=1 SV=1 | 393 | 96590 |
| sp|O75340|PDCD6_HUMAN | Programmed cell death protein 6 OS=Homo sapiens GN=PDCD6 PE=1 SV=1 | 653 | 21912 |
| Protein S100 | |  |  |
| sp|P06702|S10A9_HUMAN | Protein S100-A9 OS=Homo sapiens GN=S100A9 PE=1 SV=1 | 777 | 13291 |
| sp|P05109|S10A8_HUMAN | Protein S100-A8 OS=Homo sapiens GN=S100A8 PE=1 SV=1 | 618 | 10885 |
| sp|P60903|S10AA_HUMAN | Protein S100-A10 OS=Homo sapiens GN=S100A10 PE=1 SV=2 | 409 | 11310 |
| DNA replication licensing factor | | | |
| sp|P33993|MCM7_HUMAN | DNA replication licensing factor MCM7 OS=Homo sapiens GN=MCM7 PE=1 SV=4 | 1553 | 81884 |
| sp|P25205|MCM3_HUMAN | DNA replication licensing factor MCM3 OS=Homo sapiens GN=MCM3 PE=1 SV=3 | 297 | 91551 |
| sp|Q14566|MCM6_HUMAN | DNA replication licensing factor MCM6 OS=Homo sapiens GN=MCM6 PE=1 SV=1 | 334 | 93801 |
| Voltage-dependent anion-selective channel protein | | | |
| sp|P21796|VDAC1_HUMAN | Voltage-dependent anion-selective channel protein 1 OS=Homo sapiens GN=VDAC1 PE=1 SV=2 | 5277 | 30868 |
| sp|P45880|VDAC2_HUMAN | Voltage-dependent anion-selective channel protein 2 OS=Homo sapiens GN=VDAC2 PE=1 SV=2 | 1647 | 32060 |
| sp|Q9Y277|VDAC3_HUMAN | Voltage-dependent anion-selective channel protein 3 OS=Homo sapiens GN=VDAC3 PE=1 SV=1 | 2142 | 30981 |
| Chromosomes proteins | | |  |
| sp|A6NHR9|SMHD1_HUMAN | Structural maintenance of chromosomes flexible hinge domain-containing protein 1 OS=Homo sapiens GN=SMCHD1 PE=1 SV=2 | 579 | 227942 |
| sp|Q9NTJ3|SMC4_HUMAN | Structural maintenance of chromosomes protein 4 OS=Homo sapiens GN=SMC4 PE=1 SV=2 | 869 | 147775 |
| sp|Q14683|SMC1A_HUMAN | Structural maintenance of chromosomes protein 1A OS=Homo sapiens GN=SMC1A PE=1 SV=2 | 998 | 143771 |
| sp|Q9UQE7|SMC3_HUMAN | Structural maintenance of chromosomes protein 3 OS=Homo sapiens GN=SMC3 PE=1 SV=2 | 988 | 141853 |
| sp|O95347|SMC2_HUMAN | Structural maintenance of chromosomes protein 2 OS=Homo sapiens GN=SMC2 PE=1 SV=2 | 494 | 136085 |
| Rho-related protein | |  |  |
| sp|O75116|ROCK2_HUMAN | Rho-associated protein kinase 2 OS=Homo sapiens GN=ROCK2 PE=1 SV=4 | 402 | 161939 |
| sp|Q13464|ROCK1_HUMAN | Rho-associated protein kinase 1 OS=Homo sapiens GN=ROCK1 PE=1 SV=1 | 168 | 159102 |
| sp|P84095|RHOG_HUMAN | Rho-related GTP-binding protein RhoG OS=Homo sapiens GN=RHOG PE=1 SV=1 | 423 | 21751 |
| sp|P52566|GDIR2_HUMAN | Rho GDP-dissociation inhibitor 2 OS=Homo sapiens GN=ARHGDIB PE=1 SV=3 | 927 | 23031 |
| sp|P52565|GDIR1_HUMAN | Rho GDP-dissociation inhibitor 1 OS=Homo sapiens GN=ARHGDIA PE=1 SV=3 | 1330 | 23250 |
| sp|Q92974|ARHG2_HUMAN | Rho guanine nucleotide exchange factor 2 OS=Homo sapiens GN=ARHGEF2 PE=1 SV=4 | 848 | 112386 |
| sp|Q92888|ARHG1_HUMAN | Rho guanine nucleotide exchange factor 1 OS=Homo sapiens GN=ARHGEF1 PE=1 SV=2 | 277 | 103056 |
| sp|Q15052|ARHG6_HUMAN | Rho guanine nucleotide exchange factor 6 OS=Homo sapiens GN=ARHGEF6 PE=1 SV=2 | 287 | 88698 |
|  |  |  |  |
| sp|Q09028|RBBP4_HUMAN | Histone-binding protein RBBP4 OS=Homo sapiens GN=RBBP4 PE=1 SV=3 | 550 | 47911 |
| sp|Q16576|RBBP7_HUMAN | Histone-binding protein RBBP7 OS=Homo sapiens GN=RBBP7 PE=1 SV=1 | 317 | 48132 |
| sp|P48960|CD97_HUMAN | CD97 antigen OS=Homo sapiens GN=CD97 PE=1 SV=4 | 598 | 94603 |
| sp|Q9BTV4|TMM43_HUMAN | Transmembrane protein 43 OS=Homo sapiens GN=TMEM43 PE=1 SV=1 | 237 | 44904 |
| sp|Q9H3N1|TMX1_HUMAN | Thioredoxin-related transmembrane protein 1 OS=Homo sapiens GN=TMX1 PE=1 SV=1 | 387 | 32170 |
| sp|Q96SK2|TM209_HUMAN | Transmembrane protein 209 OS=Homo sapiens GN=TMEM209 PE=1 SV=2 | 458 | 63281 |
| sp|P42224|STAT1_HUMAN | Signal transducer and activator of transcription 1-alpha/beta OS=Homo sapiens GN=STAT1 PE=1 SV=2 | 1943 | 87850 |
| sp|P40763|STAT3_HUMAN | Signal transducer and activator of transcription 3 OS=Homo sapiens GN=STAT3 PE=1 SV=2 | 565 | 88810 |
| sp|Q12905|ILF2_HUMAN | Interleukin enhancer-binding factor 2 OS=Homo sapiens GN=ILF2 PE=1 SV=2 | 603 | 43263 |
| sp|Q12906|ILF3_HUMAN | Interleukin enhancer-binding factor 3 OS=Homo sapiens GN=ILF3 PE=1 SV=3 | 522 | 95678 |
| sp|P80303|NUCB2_HUMAN | Nucleobindin-2 OS=Homo sapiens GN=NUCB2 PE=1 SV=2 | 1003 | 50278 |
| sp|Q02818|NUCB1_HUMAN | Nucleobindin-1 OS=Homo sapiens GN=NUCB1 PE=1 SV=4 | 310 | 53846 |
| sp|P06396|GELS_HUMAN | Gelsolin OS=Homo sapiens GN=GSN PE=1 SV=1 | 4473 | 86043 |
| sp|Q9UKD2|MRT4_HUMAN | mRNA turnover protein 4 homolog OS=Homo sapiens GN=MRTO4 PE=1 SV=2 | 307 | 27657 |
| sp|Q9Y6M1|IF2B2_HUMAN | Insulin-like growth factor 2 mRNA-binding protein 2 OS=Homo sapiens GN=IGF2BP2 PE=1 SV=2 | 714 | 66195 |
| sp|P13010|XRCC5_HUMAN | X-ray repair cross-complementing protein 5 OS=Homo sapiens GN=XRCC5 PE=1 SV=3 | 7010 | 83222 |
| sp|P12956|XRCC6_HUMAN | X-ray repair cross-complementing protein 6 OS=Homo sapiens GN=XRCC6 PE=1 SV=2 | 310 | 70084 |
| sp|P61981|1433G_HUMAN | 14-3-3 protein gamma OS=Homo sapiens GN=YWHAG PE=1 SV=2 | 1019 | 27899 |
| sp|P62258|1433E_HUMAN | 14-3-3 protein epsilon OS=Homo sapiens GN=YWHAE PE=1 SV=1 | 2259 | 28032 |
| sp|P62258|1433E_HUMAN | 14-3-3 protein epsilon OS=Homo sapiens GN=YWHAE PE=1 SV=1 | 391 | 28179 |
| sp|P63104|1433Z_HUMAN | 14-3-3 protein zeta/delta OS=Homo sapiens GN=YWHAZ PE=1 SV=1 | 865 | 28372 |
| sp|Q04917|1433F_HUMAN | 14-3-3 protein eta OS=Homo sapiens GN=YWHAH PE=1 SV=4 | 248 | 28456 |
| sp|P27348|1433T_HUMAN | 14-3-3 protein theta OS=Homo sapiens GN=YWHAQ PE=1 SV=1 | 526 | 29326 |
| sp|P53618|COPB_HUMAN | Coatomer subunit beta OS=Homo sapiens GN=COPB1 PE=1 SV=3 | 955 | 98700 |
| sp|Q9Y678|COPG1_HUMAN | Coatomer subunit gamma-1 OS=Homo sapiens GN=COPG1 PE=1 SV=1 | 854 | 98967 |
| sp|Q9Y678|COPG1_HUMAN | Coatomer subunit gamma-1 OS=Homo sapiens GN=COPG1 PE=1 SV=1 | 406 | 103278 |
| sp|Q9UBF2|COPG2_HUMAN | Coatomer subunit gamma-2 OS=Homo sapiens GN=COPG2 PE=1 SV=1 | 325 | 108214 |
| sp|Q06830|PRDX1_HUMAN | Peroxiredoxin-1 OS=Homo sapiens GN=PRDX1 PE=1 SV=1 | 2176 | 22049 |
| sp|Q06830|PRDX1_HUMAN | Peroxiredoxin-1 OS=Homo sapiens GN=PRDX1 PE=1 SV=1 | 422 | 22324 |
| sp|P30041|PRDX6_HUMAN | Peroxiredoxin-6 OS=Homo sapiens GN=PRDX6 PE=1 SV=3 | 855 | 25133 |
| sp|P11215|ITAM_HUMAN | Integrin alpha-M OS=Homo sapiens GN=ITGAM PE=1 SV=2 | 1426 | 128410 |
| sp|P05107|ITB2_HUMAN | Integrin beta-2 OS=Homo sapiens GN=ITGB2 PE=1 SV=2 | 3004 | 87976 |
| sp|O14672|ADA10_HUMAN | Disintegrin and metalloproteinase domain-containing protein 10 OS=Homo sapiens GN=ADAM10 PE=1 SV=1 | 833 | 86140 |
| sp|P37802|TAGL2_HUMAN | Transgelin-2 OS=Homo sapiens GN=TAGLN2 PE=1 SV=3 | 587 | 22548 |
| sp|O00299|CLIC1_HUMAN | Chloride intracellular channel protein 1 OS=Homo sapiens GN=CLIC1 PE=1 SV=4 | 3340 | 27248 |
| sp|Q9Y696|CLIC4_HUMAN | Chloride intracellular channel protein 4 OS=Homo sapiens GN=CLIC4 PE=1 SV=4 | 356 | 28982 |
| sp|O75131|CPNE3_HUMAN | Copine-3 OS=Homo sapiens GN=CPNE3 PE=1 SV=1 | 259 | 60947 |
| sp|Q99829|CPNE1_HUMAN | Copine-1 OS=Homo sapiens GN=CPNE1 PE=1 SV=1 | 550 | 59649 |
| sp|Q86YQ8|CPNE8_HUMAN | Copine-8 OS=Homo sapiens GN=CPNE8 PE=1 SV=2 | 248 | 63638 |
| sp|P14625|ENPL_HUMAN | Endoplasmin OS=Homo sapiens GN=HSP90B1 PE=1 SV=1 | 14310 | 92696 |
| sp|Q9Y4L1|HYOU1_HUMAN | Hypoxia up-regulated protein 1 OS=Homo sapiens GN=HYOU1 PE=1 SV=1 | 1004 | 111494 |
| sp|O15031|PLXB2_HUMAN | Plexin-B2 OS=Homo sapiens GN=PLXNB2 PE=1 SV=3 | 1233 | 207734 |
| sp|O00264|PGRC1_HUMAN | Membrane-associated progesterone receptor component 1 OS=Homo sapiens GN=PGRMC1 PE=1 SV=3 | 560 | 21772 |
| sp|O15173|PGRC2_HUMAN | Membrane-associated progesterone receptor component 2 OS=Homo sapiens GN=PGRMC2 PE=1 SV=1 | 763 | 23861 |
| sp|Q14254|FLOT2_HUMAN | Flotillin-2 OS=Homo sapiens GN=FLOT2 PE=1 SV=2 | 1024 | 47434 |
| sp|O75955|FLOT1_HUMAN | Flotillin-1 OS=Homo sapiens GN=FLOT1 PE=1 SV=3 | 982 | 47554 |
| sp|P15311|EZRI_HUMAN | Ezrin OS=Homo sapiens GN=EZR PE=1 SV=4 | 732 | 69484 |
| sp|O94905|ERLN2_HUMAN | Erlin-2 OS=Homo sapiens GN=ERLIN2 PE=1 SV=1 | 1524 | 38044 |
| sp|O75477|ERLN1_HUMAN | Erlin-1 OS=Homo sapiens GN=ERLIN1 PE=1 SV=1 | 786 | 39072 |
| sp|Q70UQ0|IKIP_HUMAN | Inhibitor of nuclear factor kappa-B kinase-interacting protein OS=Homo sapiens GN=IKBIP PE=1 SV=1 | 364 | 39399 |
| sp|O43169|CYB5B_HUMAN | Cytochrome b5 type B OS=Homo sapiens GN=CYB5B PE=1 SV=2 | 383 | 13511 |
| sp|P00167|CYB5_HUMAN | Cytochrome b5 OS=Homo sapiens GN=CYB5A PE=1 SV=2 | 650 | 13522 |
| sp|P22695|QCR2_HUMAN | Cytochrome b-c1 complex subunit 2, mitochondrial OS=Homo sapiens GN=UQCRC2 PE=1 SV=3 | 1519 | 13915 |
| sp|P14927|QCR7_HUMAN | Cytochrome b-c1 complex subunit 7 OS=Homo sapiens GN=UQCRB PE=1 SV=2 | 1482 | 15321 |
| sp|Q5RI15|COX20_HUMAN | Cytochrome c oxidase protein 20 homolog OS=Homo sapiens GN=COX20 PE=1 SV=2 | 483 | 16436 |
| sp|P10606|COX5B_HUMAN | Cytochrome c oxidase subunit 5B, mitochondrial OS=Homo sapiens GN=COX5B PE=1 SV=2 | 964 | 48584 |
| sp|O60603|TLR2_HUMAN | Toll-like receptor 2 OS=Homo sapiens GN=TLR2 PE=1 SV=1 | 527 | 90920 |
| sp|Q8NF50|DOCK8_HUMAN | Dedicator of cytokinesis protein 8 OS=Homo sapiens GN=DOCK8 PE=1 SV=3 | 719 | 240886 |
| sp|Q96BY6|DOC10_HUMAN | Dedicator of cytokinesis protein 10 OS=Homo sapiens GN=DOCK10 PE=1 SV=3 | 604 | 251825 |
| sp|Q5JPE7|NOMO2_HUMAN | Nodal modulator 2 OS=Homo sapiens GN=NOMO2 PE=1 SV=1 | 1532 | 140435 |
| sp|Q15155|NOMO1_HUMAN | Nodal modulator 1 OS=Homo sapiens GN=NOMO1 PE=1 SV=5 | 1518 | 135209 |
| sp|Q8N766|EMC1_HUMAN | ER membrane protein complex subunit 1 OS=Homo sapiens GN=EMC1 PE=1 SV=1 | 633 | 112145 |
| sp|O60493|SNX3_HUMAN | Sorting nexin-3 OS=Homo sapiens GN=SNX3 PE=1 SV=3 | 674 | 18808 |
| sp|Q9Y5X3|SNX5_HUMAN | Sorting nexin-5 OS=Homo sapiens GN=SNX5 PE=1 SV=1 | 263 | 47072 |
| sp|O95881|TXD12_HUMAN | Thioredoxin domain-containing protein 12 OS=Homo sapiens GN=TXNDC12 PE=1 SV=1 | 1480 | 19365 |
| sp|Q8NBS9|TXND5_HUMAN | Thioredoxin domain-containing protein 5 OS=Homo sapiens GN=TXNDC5 PE=1 SV=2 | 562 | 48283 |
| sp|O76094|SRP72_HUMAN | Signal recognition particle subunit SRP72 OS=Homo sapiens GN=SRP72 PE=1 SV=3 | 300 | 75130 |
| sp|Q9Y5M8|SRPRB_HUMAN | Signal recognition particle receptor subunit beta OS=Homo sapiens GN=SRPRB PE=1 SV=3 | 1708 | 29912 |
| sp|Q9Y512|SAM50_HUMAN | Sorting and assembly machinery component 50 homolog OS=Homo sapiens GN=SAMM50 PE=1 SV=3 | 999 | 52342 |
| sp|Q9NR12|PDLI7_HUMAN | PDZ and LIM domain protein 7 OS=Homo sapiens GN=PDLIM7 PE=1 SV=1 | 438 | 50896 |
| sp|O00151|PDLI1_HUMAN | PDZ and LIM domain protein 1 OS=Homo sapiens GN=PDLIM1 PE=1 SV=4 | 528 | 36505 |
| sp|O94826|TOM70_HUMAN | Mitochondrial import receptor subunit TOM70 OS=Homo sapiens GN=TOMM70A PE=1 SV=1 | 789 | 68096 |
| sp|O96008|TOM40_HUMAN | Mitochondrial import receptor subunit TOM40 homolog OS=Homo sapiens GN=TOMM40 PE=1 SV=1 | 915 | 38211 |
| sp|Q9NRG9|AAAS_HUMAN | Aladin OS=Homo sapiens GN=AAAS PE=1 SV=1 | 803 | 60392 |
| sp|P40121|CAPG_HUMAN | Macrophage-capping protein OS=Homo sapiens GN=CAPG PE=1 SV=2 | 1142 | 38760 |
| sp|O43491|E41L2_HUMAN | Band 4.1-like protein 2 OS=Homo sapiens GN=EPB41L2 PE=1 SV=1 | 642 | 113032 |
| sp|Q9Y2J2|E41L3_HUMAN | Band 4.1-like protein 3 OS=Homo sapiens GN=EPB41L3 PE=1 SV=2 | 551 | 121458 |
| sp|P02786|TFR1_HUMAN | Transferrin receptor protein 1 OS=Homo sapiens GN=TFRC PE=1 SV=2 | 2041 | 85274 |
| sp|O95782|AP2A1_HUMAN | AP-2 complex subunit alpha-1 OS=Homo sapiens GN=AP2A1 PE=1 SV=3 | 1661 | 108561 |
| sp|P63010|AP2B1_HUMAN | AP-2 complex subunit beta OS=Homo sapiens GN=AP2B1 PE=1 SV=1 | 1637 | 105398 |
| sp|O94973|AP2A2_HUMAN | AP-2 complex subunit alpha-2 OS=Homo sapiens GN=AP2A2 PE=1 SV=2 | 924 | 104807 |
| sp|P26038|MOES_HUMAN | Moesin OS=Homo sapiens GN=MSN PE=1 SV=3 | 12426 | 67892 |
| sp|P27797|CALR_HUMAN | Calreticulin OS=Homo sapiens GN=CALR PE=1 SV=1 | 2161 | 48283 |
| sp|Q9BUH6|CI142_HUMAN | Uncharacterized protein C9orf142 OS=Homo sapiens GN=C9orf142 PE=1 SV=2 | 473 | 21968 |
| sp|Q8IYS2|K2013_HUMAN | Uncharacterized protein KIAA2013 OS=Homo sapiens GN=KIAA2013 PE=2 SV=1 | 302 | 69684 |
| sp|P51571|SSRD_HUMAN | Translocon-associated protein subunit delta OS=Homo sapiens GN=SSR4 PE=1 SV=1 | 677 | 19158 |
| sp|Q9P0L0|VAPA_HUMAN | Vesicle-associated membrane protein-associated protein A OS=Homo sapiens GN=VAPA PE=1 SV=3 | 996 | 28103 |
| sp|O95292|VAPB_HUMAN | Vesicle-associated membrane protein-associated protein B/C OS=Homo sapiens GN=VAPB PE=1 SV=3 | 711 | 27439 |
| sp|Q96C19|EFHD2_HUMAN | EF-hand domain-containing protein D2 OS=Homo sapiens GN=EFHD2 PE=1 SV=1 | 1572 | 26794 |
| sp|O95202|LETM1_HUMAN | LETM1 and EF-hand domain-containing protein 1, mitochondrial OS=Homo sapiens GN=LETM1 PE=1 SV=1 | 4572 | 83986 |
| sp|Q16891|IMMT_HUMAN | Mitochondrial inner membrane protein OS=Homo sapiens GN=IMMT PE=1 SV=1 | 4880 | 84026 |
| sp|P30040|ERP29_HUMAN | Endoplasmic reticulum resident protein 29 OS=Homo sapiens GN=ERP29 PE=1 SV=4 | 598 | 29032 |
| sp|Q9BS26|ERP44_HUMAN | Endoplasmic reticulum resident protein 44 OS=Homo sapiens GN=ERP44 PE=1 SV=1 | 709 | 47341 |
| sp|Q03519|TAP2_HUMAN | Antigen peptide transporter 2 OS=Homo sapiens GN=TAP2 PE=1 SV=1 | 562 | 76186 |
| sp|P04899|GNAI2_HUMAN | Guanine nucleotide-binding protein G(i) subunit alpha-2 OS=Homo sapiens GN=GNAI2 PE=1 SV=3 | 2835 | 40995 |
| sp|P08754|GNAI3_HUMAN | Guanine nucleotide-binding protein G(k) subunit alpha OS=Homo sapiens GN=GNAI3 PE=1 SV=3 | 1859 | 41076 |
| sp|P63096|GNAI1_HUMAN | Guanine nucleotide-binding protein G(i) subunit alpha-1 OS=Homo sapiens GN=GNAI1 PE=1 SV=2 | 961 | 40905 |
| sp|Q15833|STXB2_HUMAN | Syntaxin-binding protein 2 OS=Homo sapiens GN=STXBP2 PE=1 SV=2 | 448 | 66867 |
| sp|O75558|STX11_HUMAN | Syntaxin-11 OS=Homo sapiens GN=STX11 PE=2 SV=1 | 451 | 33631 |
| sp|Q12846|STX4_HUMAN | Syntaxin-4 OS=Homo sapiens GN=STX4 PE=1 SV=2 | 976 | 34273 |
| sp|P16284|PECA1_HUMAN | Platelet endothelial cell adhesion molecule OS=Homo sapiens GN=PECAM1 PE=1 SV=1 | 657 | 83396 |
| sp|P18754|RCC1_HUMAN | Regulator of chromosome condensation OS=Homo sapiens GN=RCC1 PE=1 SV=1 | 753 | 45397 |
| sp|P06748|NPM_HUMAN | Nucleophosmin OS=Homo sapiens GN=NPM1 PE=1 SV=2 | 1433 | 32726 |
| sp|Q14156|EFR3A_HUMAN | Protein EFR3 homolog A OS=Homo sapiens GN=EFR3A PE=1 SV=2 | 608 | 93777 |
| sp|O75718|CRTAP_HUMAN | Cartilage-associated protein OS=Homo sapiens GN=CRTAP PE=1 SV=1 | 622 | 47159 |
| sp|O43264|ZW10_HUMAN | Centromere/kinetochore protein zw10 homolog OS=Homo sapiens GN=ZW10 PE=1 SV=3 | 492 | 89628 |
| sp|Q8TCG1|CIP2A_HUMAN | Protein CIP2A OS=Homo sapiens GN=KIAA1524 PE=1 SV=2 | 472 | 103318 |
| sp|Q9H3U1|UN45A_HUMAN | Protein unc-45 homolog A OS=Homo sapiens GN=UNC45A PE=1 SV=1 | 404 | 104266 |
| sp|P08567|PLEK_HUMAN | Pleckstrin OS=Homo sapiens GN=PLEK PE=1 SV=3 | 364 | 40499 |
| sp|P22234|PUR6_HUMAN | Multifunctional protein ADE2 OS=Homo sapiens GN=PAICS PE=1 SV=3 | 536 | 47790 |
| sp|Q86Y56|HEAT2_HUMAN | HEAT repeat-containing protein 2 OS=Homo sapiens GN=HEATR2 PE=1 SV=4 | 417 | 94774 |
| sp|Q9UBV2|SE1L1_HUMAN | Protein sel-1 homolog 1 OS=Homo sapiens GN=SEL1L PE=1 SV=3 | 355 | 89212 |
| sp|P35241|RADI_HUMAN | Radixin OS=Homo sapiens GN=RDX PE=1 SV=1 | 3071 | 68635 |
| sp|P13796|PLSL_HUMAN | Plastin-2 OS=Homo sapiens GN=LCP1 PE=1 SV=6 | 459 | 70814 |
| sp|Q9Y240|CLC11_HUMAN | C-type lectin domain family 11 member A OS=Homo sapiens GN=CLEC11A PE=1 SV=1 | 701 | 36015 |
| sp|Q96EY7|PTCD3_HUMAN | Pentatricopeptide repeat domain-containing protein 3, mitochondrial OS=Homo sapiens GN=PTCD3 PE=1 SV=3 | 1670 | 79184 |
| sp|Q9Y4W6|AFG32_HUMAN | AFG3-like protein 2 OS=Homo sapiens GN=AFG3L2 PE=1 SV=2 | 731 | 88984 |
| sp|Q86UX7|URP2_HUMAN | Fermitin family homolog 3 OS=Homo sapiens GN=FERMT3 PE=1 SV=1 | 1828 | 76475 |
| sp|Q99439|CNN2_HUMAN | Calponin-2 OS=Homo sapiens GN=CNN2 PE=1 SV=4 | 726 | 34074 |
| sp|P54920|SNAA_HUMAN | Alpha-soluble NSF attachment protein OS=Homo sapiens GN=NAPA PE=1 SV=3 | 429 | 33667 |
| sp|P49257|LMAN1_HUMAN | Protein ERGIC-53 OS=Homo sapiens GN=LMAN1 PE=1 SV=2 | 2327 | 57798 |
| sp|P11021|GRP78_HUMAN | 78 kDa glucose-regulated protein OS=Homo sapiens GN=HSPA5 PE=1 SV=2 | 20205 | 72402 |
| sp|Q9HDC9|APMAP_HUMAN | Adipocyte plasma membrane-associated protein OS=Homo sapiens GN=APMAP PE=1 SV=2 | 851 | 46622 |
| sp|Q96A33|CCD47_HUMAN | Coiled-coil domain-containing protein 47 OS=Homo sapiens GN=CCDC47 PE=1 SV=1 | 848 | 56123 |
| sp|Q00013|EM55_HUMAN | 55 kDa erythrocyte membrane protein OS=Homo sapiens GN=MPP1 PE=1 SV=2 | 560 | 52492 |
| sp|Q12788|TBL3_HUMAN | Transducin beta-like protein 3 OS=Homo sapiens GN=TBL3 PE=1 SV=2 | 1289 | 90347 |
| sp|P17661|DESM_HUMAN | Desmin OS=Homo sapiens GN=DES PE=1 SV=3 | 392 | 53560 |
| sp|Q9H173|SIL1_HUMAN | Nucleotide exchange factor SIL1 OS=Homo sapiens GN=SIL1 PE=1 SV=1 | 228 | 52337 |
| sp|P26599|PTBP1_HUMAN | Polypyrimidine tract-binding protein 1 OS=Homo sapiens GN=PTBP1 PE=1 SV=1 | 219 | 57357 |
| sp|P22102|PUR2_HUMAN | Trifunctional purine biosynthetic protein adenosine-3 OS=Homo sapiens GN=GART PE=1 SV=1 | 524 | 108953 |
| sp|Q96A65|EXOC4_HUMAN | Exocyst complex component 4 OS=Homo sapiens GN=EXOC4 PE=1 SV=1 | 401 | 111170 |
| sp|Q9UQ80|PA2G4_HUMAN | Proliferation-associated protein 2G4 OS=Homo sapiens GN=PA2G4 PE=1 SV=3 | 513 | 44101 |
| sp|Q7Z2W4|ZCCHV_HUMAN | Zinc finger CCCH-type antiviral protein 1 OS=Homo sapiens GN=ZC3HAV1 PE=1 SV=3 | 363 | 103135 |
| sp|P15498|VAV_HUMAN | Proto-oncogene vav OS=Homo sapiens GN=VAV1 PE=1 SV=4 | 358 | 99677 |
| sp|Q86UT6|NLRX1_HUMAN | NLR family member X1 OS=Homo sapiens GN=NLRX1 PE=1 SV=1 | 348 | 108574 |
| sp|P55160|NCKPL_HUMAN | Nck-associated protein 1-like OS=Homo sapiens GN=NCKAP1L PE=1 SV=3 | 342 | 129554 |
| sp|Q9UGP8|SEC63_HUMAN | Translocation protein SEC63 homolog OS=Homo sapiens GN=SEC63 PE=1 SV=2 | 294 | 88341 |
| sp|P35613|BASI_HUMAN | Basigin OS=Homo sapiens GN=BSG PE=1 SV=2 | 360 | 42573 |
| sp|Q01826|SATB1_HUMAN | DNA-binding protein SATB1 OS=Homo sapiens GN=SATB1 PE=1 SV=1 | 322 | 86246 |
| sp|Q96SB3|NEB2_HUMAN | Neurabin-2 OS=Homo sapiens GN=PPP1R9B PE=1 SV=2 | 315 | 89309 |
| sp|Q15645|PCH2_HUMAN | Pachytene checkpoint protein 2 homolog OS=Homo sapiens GN=TRIP13 PE=1 SV=2 | 268 | 48863 |
| sp|P30533|AMRP_HUMAN | Alpha-2-macroglobulin receptor-associated protein OS=Homo sapiens GN=LRPAP1 PE=1 SV=1 | 1098 | 41441 |
| sp|Q9UJZ1|STML2_HUMAN | Stomatin-like protein 2, mitochondrial OS=Homo sapiens GN=STOML2 PE=1 SV=1 | 2372 | 38624 |
| sp|O43396|TXNL1_HUMAN | Thioredoxin-like protein 1 OS=Homo sapiens GN=TXNL1 PE=1 SV=3 | 386 | 32630 |
| sp|Q9H9B4|SFXN1_HUMAN | Sideroflexin-1 OS=Homo sapiens GN=SFXN1 PE=1 SV=4 | 729 | 35881 |
| sp|Q9BT09|CNPY3_HUMAN | Protein canopy homolog 3 OS=Homo sapiens GN=CNPY3 PE=1 SV=1 | 799 | 31128 |
| sp|P67809|YBOX1_HUMAN | Nuclease-sensitive element-binding protein 1 OS=Homo sapiens GN=YBX1 PE=1 SV=3 | 1026 | 35903 |
| sp|Q99747|SNAG_HUMAN | Gamma-soluble NSF attachment protein OS=Homo sapiens GN=NAPG PE=1 SV=1 | 317 | 35066 |
| sp|P02545|LMNA_HUMAN | Prelamin-A/C OS=Homo sapiens GN=LMNA PE=1 SV=1 | 568 | 74380 |
| sp|Q6IBS0|TWF2_HUMAN | Twinfilin-2 OS=Homo sapiens GN=TWF2 PE=1 SV=2 | 781 | 39751 |
| sp|Q96I24|FUBP3_HUMAN | Far upstream element-binding protein 3 OS=Homo sapiens GN=FUBP3 PE=1 SV=2 | 266 | 61944 |
| sp|Q9NZM1|MYOF_HUMAN | Myoferlin OS=Homo sapiens GN=MYOF PE=1 SV=1 | 665 | 236100 |
| sp|P27708|PYR1_HUMAN | CAD protein OS=Homo sapiens GN=CAD PE=1 SV=3 | 442 | 245167 |
| sp|Q14008|CKAP5_HUMAN | Cytoskeleton-associated protein 5 OS=Homo sapiens GN=CKAP5 PE=1 SV=3 | 102 | 227062 |
| sp|Q14980|NUMA1_HUMAN | Nuclear mitotic apparatus protein 1 OS=Homo sapiens GN=NUMA1 PE=1 SV=2 | 274 | 239199 |
| sp|O60449|LY75_HUMAN | Lymphocyte antigen 75 OS=Homo sapiens GN=LY75 PE=1 SV=3 | 129 | 201833 |
| sp|Q92538|GBF1_HUMAN | Golgi-specific brefeldin A-resistance guanine nucleotide exchange factor 1 OS=Homo sapiens GN=GBF1 PE=1 SV=2 | 80 | 208367 |
| sp|Q00610|CLH1_HUMAN | Clathrin heavy chain 1 OS=Homo sapiens GN=CLTC PE=1 SV=5 | 6542 | 193260 |
| sp|Q92896|GSLG1_HUMAN | Golgi apparatus protein 1 OS=Homo sapiens GN=GLG1 PE=1 SV=2 | 991 | 138341 |
| sp|O60610|DIAP1_HUMAN | Protein diaphanous homolog 1 OS=Homo sapiens GN=DIAPH1 PE=1 SV=2 | 822 | 141942 |
| sp|O60306|AQR_HUMAN | Intron-binding protein aquarius OS=Homo sapiens GN=AQR PE=1 SV=4 | 256 | 172270 |
| sp|P18206|VINC_HUMAN | Vinculin OS=Homo sapiens GN=VCL PE=1 SV=4 | 535 | 124292 |
| sp|Q16531|DDB1_HUMAN | DNA damage-binding protein 1 OS=Homo sapiens GN=DDB1 PE=1 SV=1 | 503 | 128142 |
| sp|Q9H6R4|NOL6_HUMAN | Nucleolar protein 6 OS=Homo sapiens GN=NOL6 PE=1 SV=2 | 400 | 128368 |
| sp|P33176|KINH_HUMAN | Kinesin-1 heavy chain OS=Homo sapiens GN=KIF5B PE=1 SV=1 | 311 | 110358 |
| sp|Q08379|GOGA2_HUMAN | Golgin subfamily A member 2 OS=Homo sapiens GN=GOLGA2 PE=1 SV=3 | 283 | 113644 |
| sp|Q7L576|CYFP1_HUMAN | Cytoplasmic FMR1-interacting protein 1 OS=Homo sapiens GN=CYFIP1 PE=1 SV=1 | 270 | 146742 |
| sp|O60763|USO1_HUMAN | General vesicular transport factor p115 OS=Homo sapiens GN=USO1 PE=1 SV=2 | 968 | 108740 |
| sp|Q99497|PARK7_HUMAN | Protein DJ-1 OS=Homo sapiens GN=PARK7 PE=1 SV=2 | 546 | 20050 |
| sp|Q9UBV2|SE1L1_HUMAN | Protein sel-1 homolog 1 OS=Homo sapiens GN=SEL1L PE=1 SV=3 | 966 | 89212 |
| sp|Q9BSJ8|ESYT1_HUMAN | Extended synaptotagmin-1 OS=Homo sapiens GN=ESYT1 PE=1 SV=1 | 1675 | 123293 |
| sp|Q9BXX0|EMIL2_HUMAN | EMILIN-2 OS=Homo sapiens GN=EMILIN2 PE=1 SV=3 | 1012 | 116869 |
| sp|O75396|SC22B_HUMAN | Vesicle-trafficking protein SEC22b OS=Homo sapiens GN=SEC22B PE=1 SV=4 | 1740 | 24806 |
| sp|Q9HAV7|GRPE1_HUMAN | GrpE protein homolog 1, mitochondrial OS=Homo sapiens GN=GRPEL1 PE=1 SV=2 | 1274 | 24492 |
| sp|Q99653|CHP1_HUMAN | Calcineurin B homologous protein 1 OS=Homo sapiens GN=CHP1 PE=1 SV=3 | 1051 | 22442 |
| sp|P55769|NH2L1_HUMAN | NHP2-like protein 1 OS=Homo sapiens GN=NHP2L1 PE=1 SV=3 | 508 | 14393 |
| sp|Q9GZT3|SLIRP_HUMAN | SRA stem-loop-interacting RNA-binding protein, mitochondrial OS=Homo sapiens GN=SLIRP PE=1 SV=1 | 911 | 12398 |
| sp|Q14764|MVP_HUMAN | Major vault protein OS=Homo sapiens GN=MVP PE=1 SV=4 | 1154 | 99551 |
| sp|P30086|PEBP1_HUMAN | Phosphatidylethanolamine-binding protein 1 OS=Homo sapiens GN=PEBP1 PE=1 SV=3 | 874 | 21158 |
| sp|Q9H910|HN1L_HUMAN | Hematological and neurological expressed 1-like protein OS=Homo sapiens GN=HN1L PE=1 SV=1 | 227 | 20108 |
| sp|P02792|FRIL_HUMAN | Ferritin light chain OS=Homo sapiens GN=FTL PE=1 SV=2 | 682 | 20064 |
| sp|Q9UDX5|MTFP1_HUMAN | Mitochondrial fission process protein 1 OS=Homo sapiens GN=MTFP1 PE=1 SV=1 | 257 | 18056 |
| sp|O15525|MAFG_HUMAN | Transcription factor MafG OS=Homo sapiens GN=MAFG PE=1 SV=1 | 396 | 17896 |
| sp|O60234|GMFG_HUMAN | Glia maturation factor gamma OS=Homo sapiens GN=GMFG PE=1 SV=1 | 683 | 16961 |
| sp|P62158|CALM_HUMAN | Calmodulin OS=Homo sapiens GN=CALM1 PE=1 SV=2 | 664 | 16827 |
| sp|Q9Y6C9|MTCH2_HUMAN | Mitochondrial carrier homolog 2 OS=Homo sapiens GN=MTCH2 PE=1 SV=1 | 1403 | 33936 |
| sp|Q14165|MLEC_HUMAN | Malectin OS=Homo sapiens GN=MLEC PE=1 SV=1 | 849 | 32385 |
| sp|P27105|STOM_HUMAN | Erythrocyte band 7 integral membrane protein OS=Homo sapiens GN=STOM PE=1 SV=3 | 540 | 31882 |
| sp|O00541|PESC_HUMAN | Pescadillo homolog OS=Homo sapiens GN=PES1 PE=1 SV=1 | 186 | 68359 |
| sp|Q96CN7|ISOC1_HUMAN | Isochorismatase domain-containing protein 1 OS=Homo sapiens GN=ISOC1 PE=1 SV=3 | 346 | 32501 |
| sp|P50454|SERPH_HUMAN | Serpin H1 OS=Homo sapiens GN=SERPINH1 PE=1 SV=2 | 1115 | 46525 |
| sp|O60664|PLIN3_HUMAN | Perilipin-3 OS=Homo sapiens GN=PLIN3 PE=1 SV=3 | 1053 | 47217 |
| sp|Q99536|VAT1_HUMAN | Synaptic vesicle membrane protein VAT-1 homolog OS=Homo sapiens GN=VAT1 PE=1 SV=2 | 799 | 42122 |
| sp|Q10567|AP1B1_HUMAN | AP-1 complex subunit beta-1 OS=Homo sapiens GN=AP1B1 PE=1 SV=2 | 780 | 105482 |
| sp|O00165|HAX1_HUMAN | HCLS1-associated protein X-1 OS=Homo sapiens GN=HAX1 PE=1 SV=2 | 270 | 31601 |
| sp|Q07021|C1QBP_HUMAN | Complement component 1 Q subcomponent-binding protein, mitochondrial OS=Homo sapiens GN=C1QBP PE=1 SV=1 | 1423 | 31742 |
| sp|P19338|NUCL_HUMAN | Nucleolin OS=Homo sapiens GN=NCL PE=1 SV=3 | 201 | 76625 |
| sp|Q15691|MARE1_HUMAN | Microtubule-associated protein RP/EB family member 1 OS=Homo sapiens GN=MAPRE1 PE=1 SV=3 | 581 | 30151 |
| sp|O00560|SDCB1_HUMAN | Syntenin-1 OS=Homo sapiens GN=SDCBP PE=1 SV=1 | 475 | 32595 |
| sp|Q9H223|EHD4_HUMAN | EH domain-containing protein 4 OS=Homo sapiens GN=EHD4 PE=1 SV=1 | 396 | 61365 |
| sp|P07384|CAN1_HUMAN | Calpain-1 catalytic subunit OS=Homo sapiens GN=CAPN1 PE=1 SV=1 | 480 | 82465 |
| sp|Q15437|SC23B_HUMAN | Protein transport protein Sec23B OS=Homo sapiens GN=SEC23B PE=1 SV=2 | 443 | 87393 |
| sp|Q96T51|RUFY1_HUMAN | RUN and FYVE domain-containing protein 1 OS=Homo sapiens GN=RUFY1 PE=1 SV=2 | 405 | 80851 |
| sp|Q9Y3L3|3BP1_HUMAN | SH3 domain-binding protein 1 OS=Homo sapiens GN=SH3BP1 PE=1 SV=3 | 315 | 76008 |
| sp|Q9UH99|SUN2_HUMAN | SUN domain-containing protein 2 OS=Homo sapiens GN=SUN2 PE=1 SV=3 | 390 | 80490 |
| sp|P33897|ABCD1_HUMAN | ATP-binding cassette sub-family D member 1 OS=Homo sapiens GN=ABCD1 PE=1 SV=2 | 379 | 83398 |
| sp|Q13438|OS9_HUMAN | Protein OS-9 OS=Homo sapiens GN=OS9 PE=1 SV=1 | 295 | 75971 |
| sp|P08311|CATG_HUMAN | Cathepsin G OS=Homo sapiens GN=CTSG PE=1 SV=2 | 327 | 29161 |
| sp|Q969V3|NCLN_HUMAN | Nicalin OS=Homo sapiens GN=NCLN PE=1 SV=2 | 1835 | 63106 |
| sp|P38117|ETFB_HUMAN | Electron transfer flavoprotein subunit beta OS=Homo sapiens GN=ETFB PE=1 SV=3 | 273 | 28054 |
| sp|O00161|SNP23_HUMAN | Synaptosomal-associated protein 23 OS=Homo sapiens GN=SNAP23 PE=1 SV=1 | 678 | 23682 |
| sp|Q15233|NONO_HUMAN | Non-POU domain-containing octamer-binding protein OS=Homo sapiens GN=NONO PE=1 SV=4 | 719 | 54311 |
| sp|Q92882|OSTF1_HUMAN | Osteoclast-stimulating factor 1 OS=Homo sapiens GN=OSTF1 PE=1 SV=2 | 541 | 23943 |
| sp|O43399|TPD54_HUMAN | Tumor protein D54 OS=Homo sapiens GN=TPD52L2 PE=1 SV=2 | 469 | 22281 |
| sp|P62993|GRB2_HUMAN | Growth factor receptor-bound protein 2 OS=Homo sapiens GN=GRB2 PE=1 SV=1 | 312 | 25304 |
| sp|Q9Y5Z4|HEBP2_HUMAN | Heme-binding protein 2 OS=Homo sapiens GN=HEBP2 PE=1 SV=1 | 294 | 22861 |
